# Anticancer Diiron Vinyliminium Complexes: A Structure–Activity Relationship Study

**DOI:** 10.3390/pharmaceutics13081158

**Published:** 2021-07-27

**Authors:** Simona Braccini, Giorgia Rizzi, Lorenzo Biancalana, Alessandro Pratesi, Stefano Zacchini, Guido Pampaloni, Federica Chiellini, Fabio Marchetti

**Affiliations:** 1 Department of Chemistry and Industrial Chemistry, University of Pisa, Via G. Moruzzi 13, I-56124 Pisa, Italy; simona.braccini@phd.unipi.it (S.B.); giorgia.rizzi17@gmail.com (G.R.); lorenzo.biancalana@unipi.it (L.B.); alessandro.pratesi@unipi.it (A.P.); guido.pampaloni@unipi.it (G.P.); 2 Department of Industrial Chemistry “Toso Montanari”, University of Bologna, Viale Risorgimento 4, I-40136 Bologna, Italy; stefano.zacchini@unibo.it

**Keywords:** metal-based drugs, diiron complexes, cytotoxicity, ROS production, thioredoxin reductase inhibition

## Abstract

A series of 16 novel diiron complexes of general formula [Fe_2_Cp_2_(CO)(μ-CO){μ-η^1^:η^3^-C(R′)C(R″)CN(R)(Y)}]CF_3_SO_3_ (**2–7**), bearing different substituents on the bridging vinyliminium ligand, was synthesized in 69–95% yields from the reactions of diiron μ-aminocarbyne precursors with various alkynes. The products were characterized by elemental analysis, IR, ^1^H and ^13^C NMR spectroscopy; moreover the X-ray structures of **2c** (R = Y = CH_2_Ph, R′ = R″ = Me) and **3a** (R = CH_2_CH=CH_2_, Y = R′ = Me, R″ = H) were ascertained by single-crystal X-ray diffraction studies. NMR and UV–Vis methods were used to assess the D_2_O solubility, the stability in aqueous solution at 37 °C and the octanol–water partition coefficients of the complexes. A screening study evidenced a potent cytotoxicity of **2–7** against the A2780 cancer cell line, with a remarkable selectivity compared to the nontumoral Balb/3T3 cell line; complex **4c** (R = Cy, Y = R′ = R″ = Me) revealed as the most performant of the series. The antiproliferative activity of a selection of complexes was also assessed on the cisplatin-resistant A2780cisR cancer cell line, and these complexes were capable of inducing a significant ROS production. Moreover, ESI-MS experiments indicated the absence of interaction of selected complexes with cytochrome c and the potentiality to inhibit the thioredoxin reductase enzyme (TrxR).

## 1. Introduction

**Figure 1:**
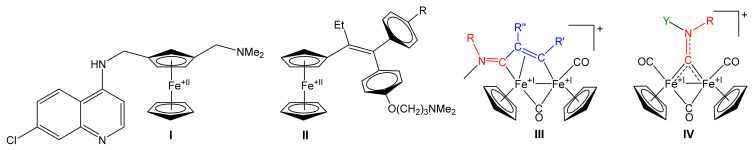
Structures of selected iron complexes with reported biological (anticancer) activity. a) Ferrocene derivatives: (**I**) ferroquine, antimalarial drug investigated in clinical trials; (**II**) ferrocifens (R=H, OH), derived from conjugation of ferrocene with the anticancer drug tamoxifen; (**III**) Diiron μ-vinyliminium complexes with structural variability from combination of isocyanide (CNR, red) and alkyne (R′C≡CR″, blue) reagents (R=Me, 2,6-C_6_H_3_Me_2_); (**IV**) Diiron μ-aminocarbyne complexes able to transfer Y^+^ (green) to bio-substrates.

There is an urgent demand for the development of new, effective and targeted anticancer drugs, and in this regard transition metal complexes are at the forefront of research [1,2,3,4,5,6]. Indeed, this category of compounds offers peculiar properties associated with the presence of one or more transition elements, i.e., the availability of a variety of oxidation states, coordination environments and geometries, and the possibility of replacement and/or activation of ligands under suitable conditions [7,8,9]. Such arsenal of tools may provide an increased added pharmaceutical value with respect to common organic molecules, and in this light few platinum complexes have been successfully employed worldwide in clinical treatments against several types of tumors, in combination with other drugs [10,11]. On the other hand, the toxicity of the metal center may represent a serious issue, and this is certainly the case of platinum, a “heavy metal” belonging to the 5d series [12,13]. The severe side effects arising from this toxicity, along with a limited selectivity of action, and the tendency of the tumor cells to progressively acquire resistance to the treatment represent major inconveniences in the administration of platinum chemotherapics, besides their unquestionable efficacy [14,15,16]. Therefore, a huge effort has been devoted to evaluating potential drugs based on other transition metals [2]. Iron is an attractive element in this respect due to its bioavailability, which substantially limits the toxic effects of its compounds, and the feasible redox chemistry in physiological media, usually involving the +II and +III oxidation states [17,18]. A variety of monoiron complexes have been assessed for the anticancer potential both in vitro and in vivo [19,20,21,22]. Following the successful experience with ferroquine, a conjugate between ferrocene and the drug chloroquine, which entered phase II clinical trials as an antimalarial agent (Figure 1, structure **I**) [4,23,24], the anticancer properties of related ferrocene derivatives have been intensively investigated (an example in Figure 1, structure **II**) [25,26,27,28]. This family of compounds exhibits a substantial robustness supplied by the ferrocene skeleton and exerts a cytotoxic activity essentially by unbalancing cellular redox homeostasis via iron(II) to iron(III) oxidation [25,26,27,28,29]. Such a mode of action strikingly differs from that of platinum compounds, which instead induce cell death through DNA binding [10,30]. On the wave of our longtime experience with organo-iron synthetic chemistry [31,32,33,34,35], in the very last years we have contributed to unveil the anticancer potential of diiron complexes, which was almost unexplored [36,37,38,39,40]. This is surprising, in that diiron complexes hold a major advantage, compared to related monoiron species, due to the presence of adjacent metal atoms “working in concert”, thus allowing the easy construction and the stabilization of uncommon bridging ligands and the co-presence of other ligands [31,41,42,43]. In this context, commercial [Fe_2_Cp_2_(CO)_4_] (Cp = η^5^-C_5_H_5_) is a convenient starting material based on a [Fe^+I^-Fe^+I^] core, with the complementary electronic properties of the CO and Cp ligands providing robustness. Cationic complexes of type **III** (Figure 1), containing a tightly coordinated bridging vinyliminium ligand, originate from the stepwise coupling of one isocyanide with one alkyne [44]. Complexes **III** are rather stable in aqueous media and display a variable cytotoxicity, ranging from the micromolar range to inactivity; they appear to exert their action following the general behavior shown by cytotoxic iron compounds, i.e., through the interference with redox processes [36,37,38,39,40,45]. Notwithstanding, other ways that are not accessible to ferrocene derivatives might be viable, including binding to biological targets [36,37,38,39] and the auxiliary effect of slow carbon monoxide release [37,40,45]. The structural diversity offered by the choice of the isocyanide (R substituent) and alkyne (R′, R″) reagents enables to tune important physico-chemical properties of the complexes (e.g., water solubility, amphiphilicity), correlated to their activity. A wide range of alkynes has been explored in this regard, including the incorporation of specific bioactive fragments [46], whereas the variation of the R group has been quite restricted (compounds with R = Me or R = 2,6-C_6_H_3_Me_2_ = Xyl have been preferentially studied). This point is not trivial, on considering that related diiron complexes with a bridging aminocarbyne (iminium) ligand (structure **IV**, Figure 1) possess the ability to interact with bio-substrates, which seems strongly affected by both N-substituents, Y and R [37]. Herein, we report the synthesis and the full characterization of novel diiron vinyliminium complexes, containing unprecedented combinations of iminium substituents, and related studies aimed to assess the antiproliferative activity and to clarify the mechanism of action.

## 2. Experimental

### 2.1. Materials and Methods

**Scheme 1:**
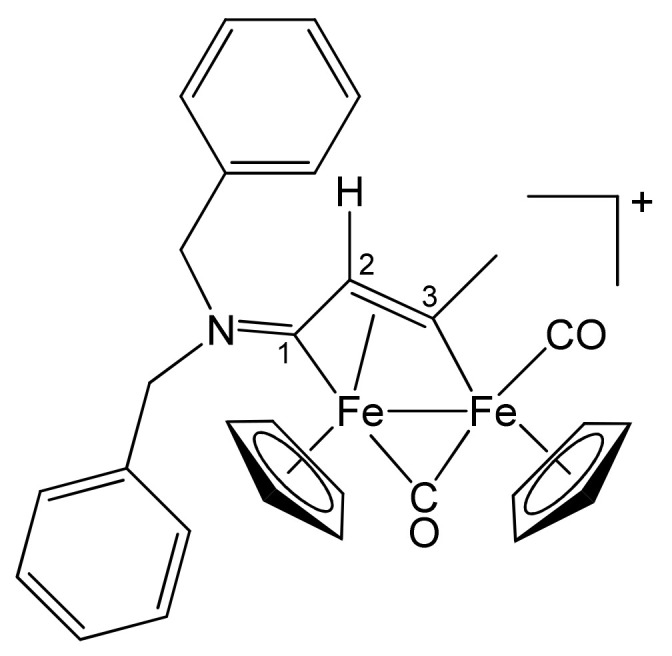
Structure of the cation of **2a**.

**Scheme 2:**
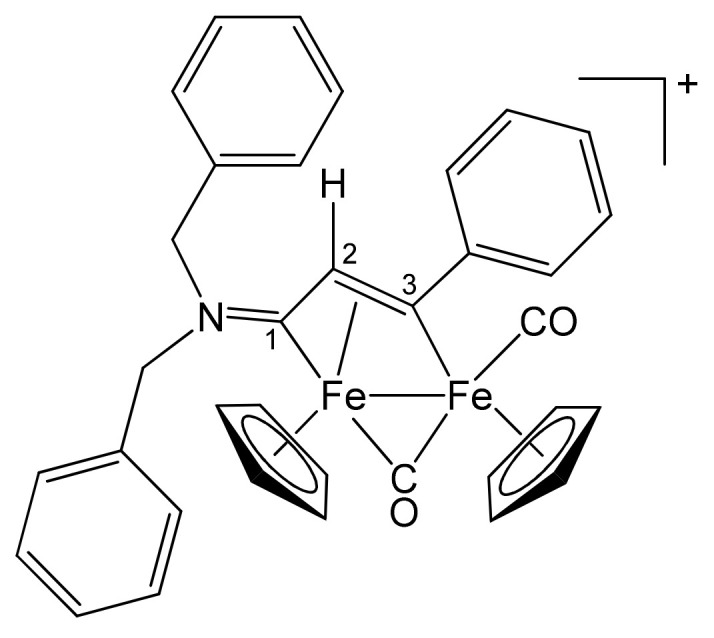
Structure of the cation of **2b**.

**Scheme 3:**
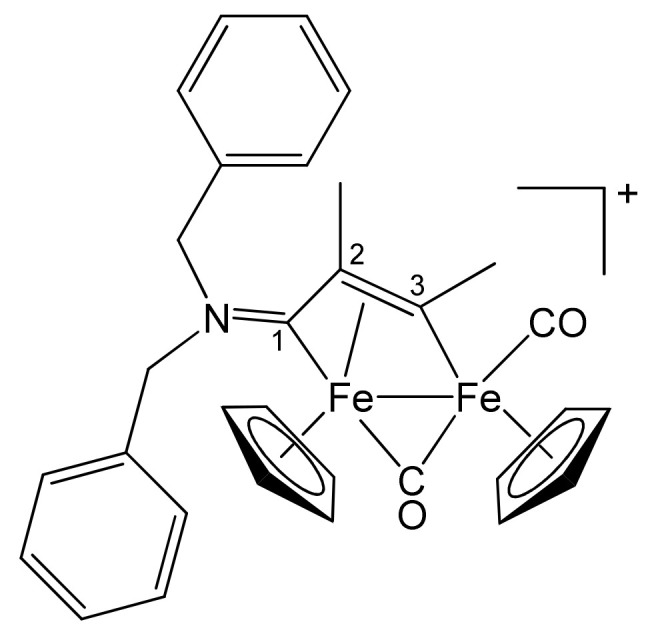
Structure of the cation of **2c**.

**Scheme 4:**
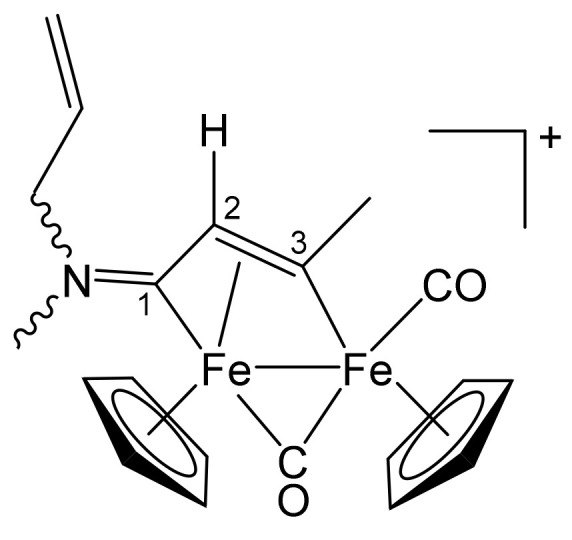
Structure of the cation of **3a**.

**Scheme 5:**
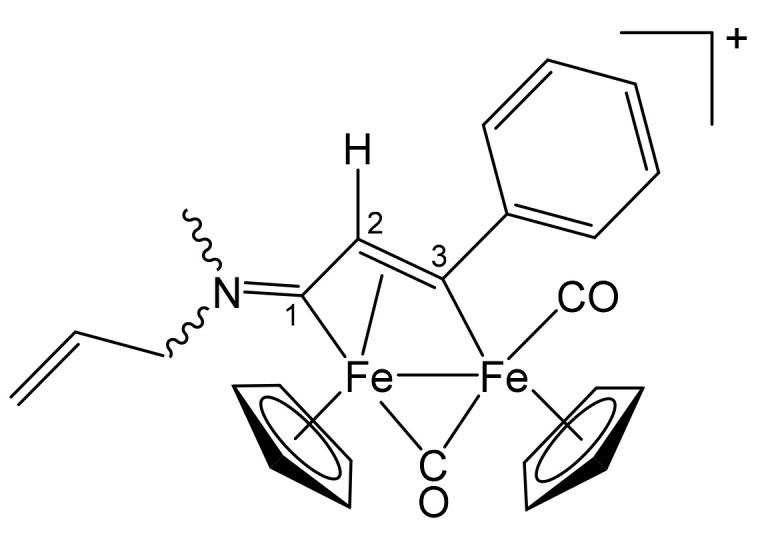
Structure of the cation of **3b**.

**Scheme 6:**
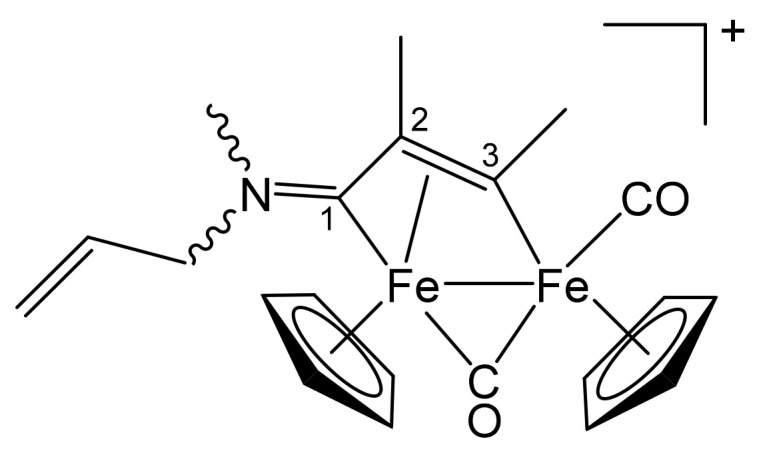
Structure of the cation of **3c**.

**Scheme 7:**
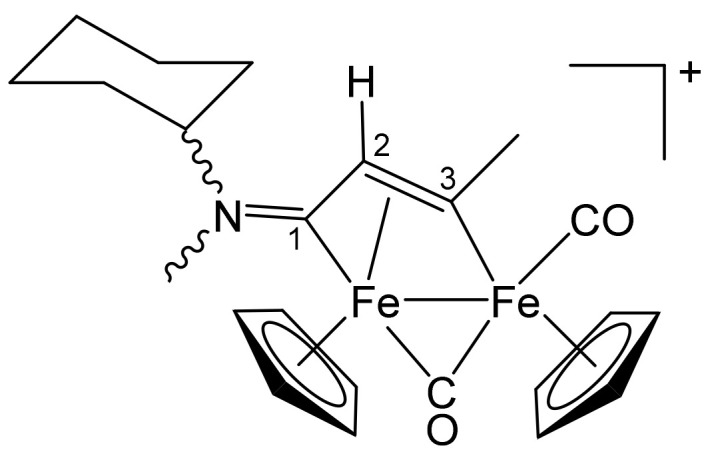
Structure of the cation of **4a**.

**Scheme 8:**
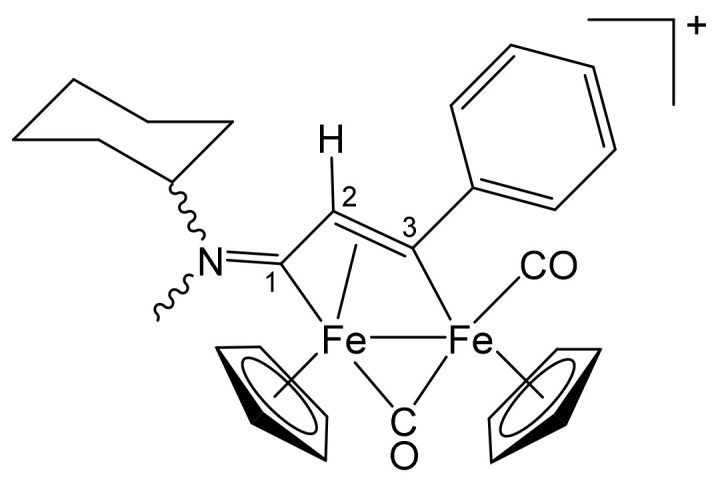
Structure of the cation of **4b**.

**Scheme 9:**
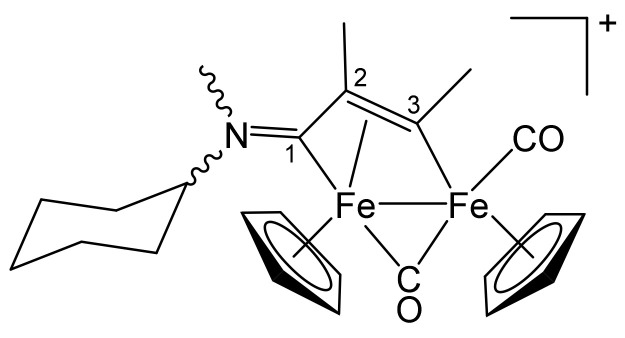
Structure of the cation of **4c**.

**Scheme 10:**
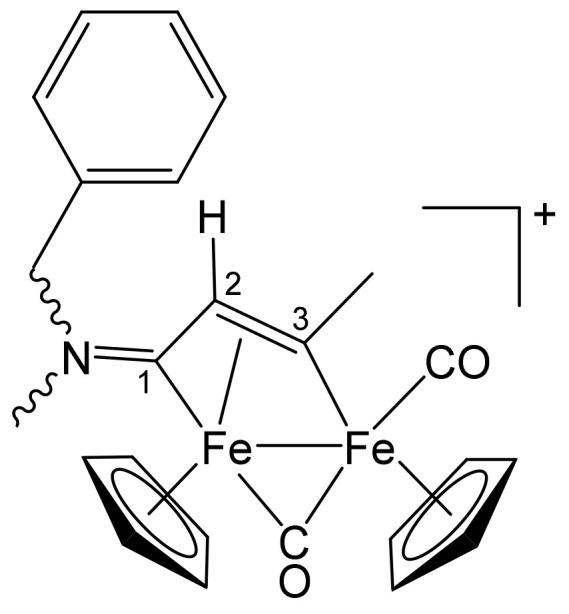
Structure of the cation of **5a**.

**Scheme 11:**
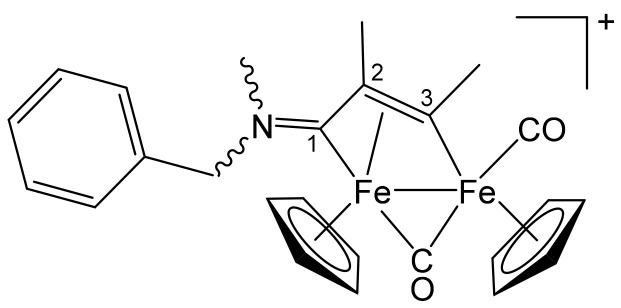
Structure of the cation of **5b**.

**Scheme 12:**
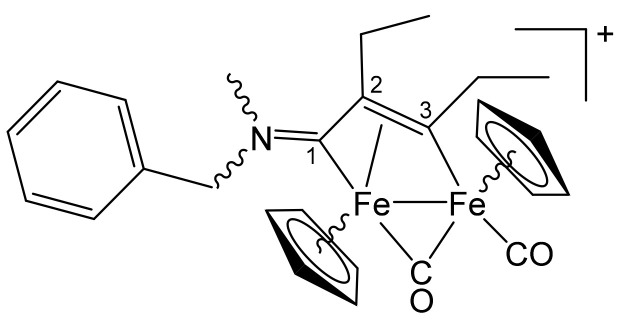
Structure of the cation of **5c**.

**Scheme 13:**
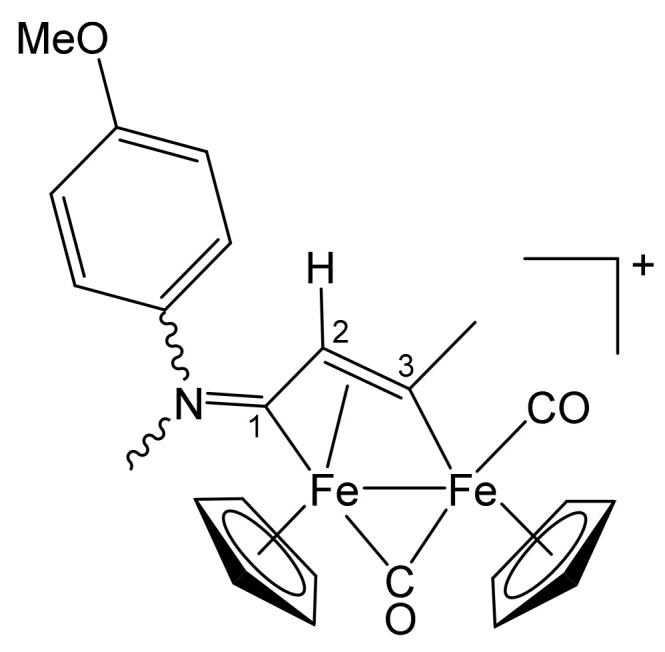
Structure of the cation of **6a**.

**Scheme 14:**
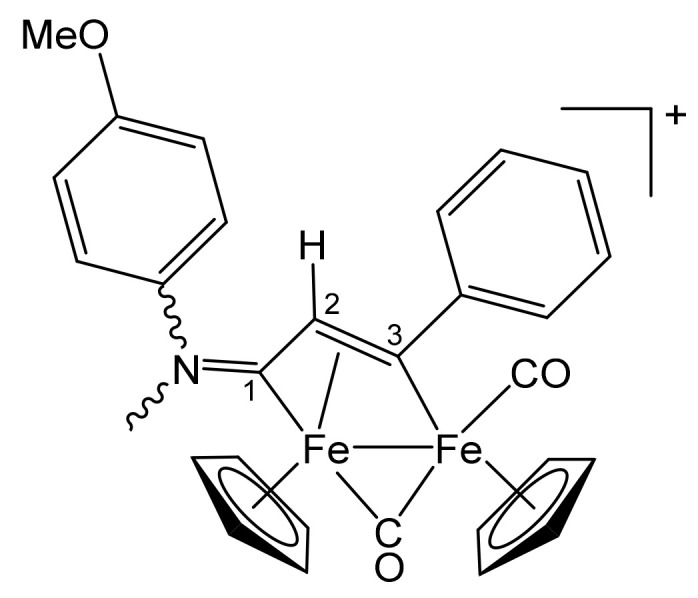
Structure of the cation of **6b**.

**Scheme 15:**
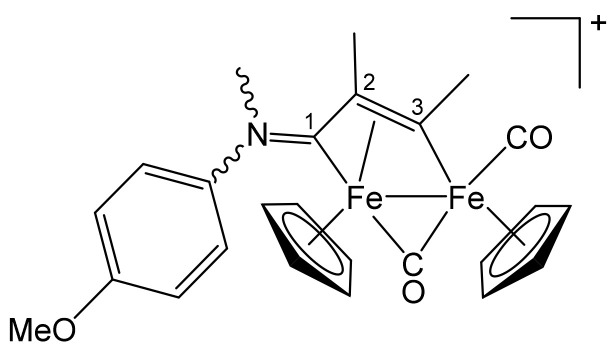
Structure of the cation of **6c**.

**Scheme 16:**
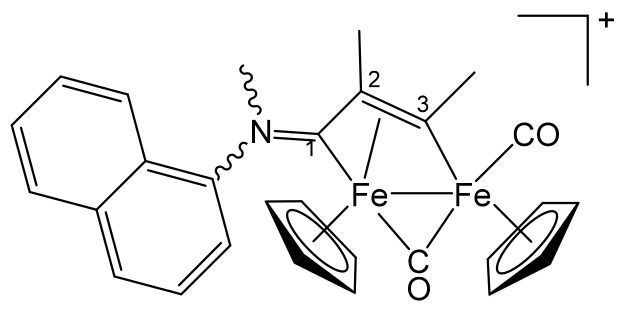
Structure of the cation of **7**.

The preparation and purification of complexes were carried out in air, and isolated products were stored in air. Solvents and organic reactants were purchased from Merck or TCI Europe. Compounds **1a–c,e** [37] and **1d,f** [47] were prepared according to the respective published procedures. Chromatography separations were carried out on columns of deactivated alumina (Merck, 4% *w*/*w* water). Infrared spectra of solutions were recorded on a Perkin Elmer Spectrum 100 FT-IR spectrometer with a CaF_2_ liquid transmission cell (2300–1500 cm^−1^ range). IR spectra were processed with Spectragryph software [48]. NMR spectra were recorded at 298 K on a Bruker Avance II DRX400 instrument equipped with a BBFO broadband probe. Chemical shifts (expressed in parts per million) are referenced to the residual solvent peaks (^1^H, ^13^C) [49] or to external standard (H_3_PO_4_, ^31^P). ^1^H and ^13^C NMR spectra were assigned with the assistance of ^1^H-^13^C (*gs*-HSQC and *gs*-HMBC) correlation experiments [50]. NMR signals due to a second isomeric form (where it has been possible to detect them) are italicized; integration values refer to the main isomer. Scheme 1, Scheme 2, Scheme 3, Scheme 4, Scheme 5, Scheme 6, Scheme 7, Scheme 8, Scheme 9, Scheme 10, Scheme 11, Scheme 12, Scheme 13, Scheme 14, Scheme 15 and Scheme 16 show the prevalent isomeric form detected by NMR in each case. Elemental analyses were performed on a Vario MICRO cube instrument.

### 2.2. Synthesis and Characterization of Diiron Complexes

General procedure. A solution of **1a–f** (ca. 0.5 mmol) in acetonitrile (ca. 10 mL) was treated with Me_3_NO (1.2 eq.). The resulting mixture was stirred for 1 h, during which time progressive color darkening was noticed. The complete conversion of the starting material into the corresponding CO/NCMe substitution product was clearly checked by IR spectroscopy [51,52,53]. The volatiles were removed under vacuum to afford a dark-brown residue, which was dissolved into dichloromethane (ca. 20 mL) and treated with the appropriate alkyne (1.5–2.0 eq.). The mixture was stirred at room temperature for 48 h, then it was charged on an alumina column. Elution with CH_2_Cl_2_ and then CH_2_Cl_2_/THF (1:1 *v*/*v*) allowed to remove the excess of alkyne and impurities. The fraction corresponding to the product was separated using the appropriate eluent (vide infra), then the solvent was evaporated under reduced pressure. The residue was dissolved in the minimum volume of CH_2_Cl_2_, and subsequent addition of hexane (20–30 mL) gave a powder which was dried under vacuum.

**[Fe_2_Cp_2_(CO)(μ-CO){μ-η^1^:η^3^-C^3^(Me)C^2^HC^1^N(CH_2_Ph)_2_}]CF_3_SO_3_, 2a (** Scheme 1 **)**

From **1a** and propyne (THF solution, ca. 1 mol/L). Brownish-red solid, yield 81%. Eluent for chromatography: MeCN. Anal. calcd. for C_31_H_28_F_3_Fe_2_NO_5_S: C, 53.55; H, 4.06; N, 2.01; S, 4.61. Found: C, 53.41; H, 4.12; N, 1.97; S, 4.55. IR (CH_2_Cl_2_): ῦ/cm^−1^ = 1991vs (CO), 1808s (μ-CO), 1644m (C^1^ = N). ^1^H NMR (acetone-d_6_): δ/ppm = 7.44, 7.21, 7.10 (m, 10 H, Ph); 5.86, 5.22 (d, 2 H, ^2^J_HH_ = 14.7 Hz, CH_2_); 5.66, 5.32 (s, 10 H, Cp); 4.90 (s, 1 H, C^2^H); 4.65, 4.33 (d, 2 H, ^2^J_HH_ = 14.7 Hz, CH_2_); 4.04 (s, 3 H, C^3^Me). ^13^C{^1^H} NMR (acetone-d_6_): δ/ppm = 257.1 (μ-CO); 229.4 (C^1^); 210.7 (CO); 210.2 (C^3^); 132.7, 132.3, 129.1, 129.0, 128.9, 128.8 (Ph); 91.2, 88.0 (Cp); 64.2, 59.4 (CH_2_); 52.9 (C^2^), 41.4 (C^3^*Me*).

**[Fe_2_Cp_2_(CO)(μ-CO){μ-η^1^:η^3^-C^3^(Ph)C^2^HC^1^N(CH_2_Ph)_2_}]CF_3_SO_3_, 2b (** Scheme 2 **)**

From **1a** and phenylacetylene. Brown solid, yield 81%. Eluent for chromatography: MeCN. Anal. calcd. for C_36_H_30_F_3_Fe_2_NO_5_S: C, 57.09; H, 3.99; N, 1.85; S, 4.23. Found: C, 56.88; H, 4.03; N, 1.88; S, 4.30. IR (CH_2_Cl_2_): ῦ/cm^−1^ = 1992vs (CO), 1810s (μ-CO), 1642m (C^1^ = N). ^1^H NMR (acetone-d_6_): δ/ppm = 7.79, 7.57, 7.46, 7.40, 7.31, 7.10 (m, 15 H, Ph); 5.94, 4.48 (d, 2 H, ^2^J_HH_ = 14.67 Hz, CH_2_); 5.50, 5.34 (s, 10 H, Cp); 5.43, 4.72 (d, 2 H, ^2^J_HH_ = 13.69 Hz, CH_2_); 4.75 (s, 1 H, C^2^H). ^13^C{^1^H} NMR (acetone-d_6_): δ/ppm = 256.6 (μ-CO); 227.8 (C^1^); 210.0 (CO); 156.4 (*ipso*-Ph); 132.8, 132.2, 129.2, 129.1, 128.4, 127.3, 127.0 (Ph); 92.2, 88.2 (Cp); 63.9, 60.0 (CH_2_); 53.5 (C^2^). C^3^ signal overlapped with solvent.

**[Fe_2_Cp_2_(CO)(μ-CO){μ-η^1^:η^3^-C^3^(Me)C^2^(Me)C^1^N(CH_2_Ph)_2_}]CF_3_SO_3_, 2c (** Scheme 3 **)**

From **1a** and 2-butyne. Brown solid, yield 84%. Eluent for chromatography: MeCN. Anal. calcd. for C_32_H_30_F_3_Fe_2_NO_5_S: C, 54.18; H, 4.26; N, 1.97; S, 4.52. Found: C, 54.08; H, 4.32; N, 1.90; S, 4.57. IR (CH_2_Cl_2_): ῦ/cm^−1^ = 1987vs (CO), 1805s (μ-CO), 1628m (C^1^ = N). ^1^H NMR (acetone-d_6_): δ/ppm = 7.38, 7.05 (m, 10 H, Ph); 5.70, 5.45 (d, 2 H, ^2^J_HH_ = 14.7 Hz, CH_2_); 4.78, 4.27 (d, 2 H, ^2^J_HH_ = 15.3 Hz, CH_2_); 5.64, 5.29 (s, 10 H, Cp); 4.00 (s, 3 H, C^3^Me); 2.19 (s, 3 H, C^2^Me). ^13^C{^1^H} NMR (acetone-d_6_): δ/ppm = 258.2 (μ-CO); 230.4 (C^1^); 210.9 (CO); 203.7 (C^3^); 132.6, 131.6, 128.9, 128.3 (Ph); 91.6, 88.6 (Cp); 65.7 (C^2^); 63.1, 58.4 (CH_2_); 36.8 (C^3^*Me*); 15.9 (C^2^*Me*). Crystals suitable for X-ray analysis were obtained by slow evaporation of the solvent from an acetone solution of **2c**.

**[Fe_2_Cp_2_(CO)(μ-CO){μ-η^1^:η^3^-C^3^(Me)C^2^HC^1^NMe(CH_2_CH = CH_2_)}]CF_3_SO_3_, 3a (** Scheme 4 **)**

From **1b** and propyne (THF solution, ca. 1 mol/L). Brown solid, yield 76%. Eluent for chromatography: THF/MeCN 1:1. Anal. calcd. for C_21_H_22_F_3_Fe_2_NO_5_S: C, 44.32; H, 3.90; N, 2.46; S, 5.63. Found: C, 44.21; H, 3.96; N, 2.37; S, 5.70. IR (CH_2_Cl_2_): ῦ/cm^−1^ = 1990vs (CO), 1807s (μ-CO), 1669m (C^1^ = N), 1643w (C = C). ^1^H NMR (acetone-d_6_): δ/ppm = *6.04*, 5.75 (m, 1 H, C*H* = CH_2_); 5.61, 5.51, 5.49, 5.39 (m, 2 H, CH = C*H*_2_); 5.55, *5.22*, 5.21 (s, 10 H, Cp); 4.98, 4.90, *4.17* (m, ^3^J_HH_ = 6.4 Hz, NCH_2_); 4.70, *4.67* (s, 1 H, C^2^H); 3.99 (s, 3 H, C^3^Me); *3.92*, 3.27 (s, 3 H, NMe). Isomer ratio (E/Z) = 1.2. ^13^C{^1^H} NMR (acetone-d_6_): δ/ppm = 257.3, *256.6* (μ-CO); *227.3*, 226.9 (C^1^); 210.7, 208.4 (CO); 205.5 (C^3^); 130.7, *129.7* (*C*H = CH_2_); *121.5*, 121.3 (CH = *C*H_2_); 90.8, *87.8*, 87.7 (Cp); *66.7*, 60.4 (NCH_2_); 52.4, *52.1* (C^2^); *47.3*, 41.8 (NMe); 41.2 (C^3^*Me*). Crystals suitable for X-ray analysis were obtained by slow diffusion of diethyl ether into a solution of **3a** in CH_2_Cl_2_, at −30 °C.

**[Fe_2_Cp_2_(CO)(μ-CO){μ-η^1^:η^3^-C^3^(Ph)C^2^HC^1^NMe(CH_2_CH = CH_2_)}]CF_3_SO_3_, 3b (** Scheme 5 **)**

From **1b** and phenylacetylene. Brown solid, yield 79%. Eluent for chromatography: MeCN. Anal. calcd. for C_26_H_24_F_3_Fe_2_NO_5_S: C, 49.47; H, 3.83; N, 2.22; S, 5.08. Found: C, 49.55; H, 3.81; N, 2.29; S, 5.13. IR (CH_2_Cl_2_): ῦ/cm^−1^ = 1993vs (CO), 1809s (μ-CO), 1666m (C^1^ = N), 1643w (C = C). ^1^H NMR (CDCl_3_): δ/ppm = 7.56, 7.41 (m, 5 H, Ph); 5.85 (br, 1 H, C*H* = CH_2_); 5.64, 5.54, 5.35, 5.31 (m, 2 H, CH = C*H*_2_); 5.23, *5.21*, 4.97 (s, 10 H, Cp); 4.34, 4.17 (m, 2 H, NCH_2_); *4.76*, 4.69 (s, 1 H, C^2^H); 3.89, *3.30* (s, 3 H, NMe). Isomer ratio (Z/E) = 1.4. ^13^C{^1^H} NMR (acetone-d_6_): δ/ppm = *256.3*, 255.6 (μ-CO); 226.1, *225.1* (C^1^); 210.1, *210.0* (CO); *204.4*, 204.3 (C^3^); 156.4, *156.3* (*ipso*-Ph); *130.7*, 129.8 (*C*H = CH_2_); *128.4*, 128.3, *127.4*, 127.3, *127.0*, 126.9 (Ph); 121.6, *121.4* (CH = *C*H_2_); 91.7, 87.9, *87.8* (Cp); 67.0, *60.3* (NCH_2_); *53.0*, 52.8 (C^2^); 47.7, *41.8* (NMe).

**[Fe_2_Cp_2_(CO)(μ-CO){μ-η^1^:η^3^-C^3^(Me)C^2^(Me)C^1^NMe(CH_2_CH = CH_2_)}]CF_3_SO_3_, 3c (** Scheme 6 **)**

From **1b** and 2-butyne. Brown solid, yield 94%. Eluent for chromatography: MeCN. Anal. calcd. for C_22_H_24_F_3_Fe_2_NO_5_S: C, 45.31; H, 4.15; N, 2.40; S, 5.50. Found: C, 45.17; H, 4.21; N, 2.31; S, 5.55. IR (CH_2_Cl_2_): ῦ/cm^−1^ = 1986vs (CO), 1805s (μ-CO), 1656m (C^1^ = N), 1639w (C = C). ^1^H NMR (acetone-d_6_): δ/ppm = 6.05, 5.80 (m, 1 H, C*H* = CH_2_); 5.62, 5.55, 5.41, 5.38 (m, 2 H, CH = C*H*_2_); 5.52, 5.51, 5.20, 5.19 (s, 10 H, Cp); 5.05–4.87, 4.20, 4.03 (m, 2 H, NCH_2_); 3.91 (s, 3 H, C^3^Me); 3.89, *3.21* (s, 3 H, NMe); 2.04 (s, 3 H, C^2^Me). Isomer ratio (Z/E) = 1.2. ^13^C{^1^H} NMR (acetone-d_6_): δ/ppm = 258.1, 257.4 (μ-CO); 227.3, 226.5 (C^1^); *210.8*, 210.6 (CO); 202.1, 201.9 (C^3^); *130.8*, 129.8 (*C*H = CH_2_); 121.7, *121.*5 (CH = *C*H_2_); 91.0, 88.2, *88.1* (Cp); 65.5 (C^2^); 65.4, *60.5* (NCH_2_); 45.3, 41.3 (NMe); 36.2 (C^3^*Me*); 15.4, *14.8* (C^2^*Me*).

**[Fe_2_Cp_2_(CO)(μ-CO){μ-η^1^:η^3^-C^3^(Me)C^2^HC^1^NMe(Cy)}]CF_3_SO_3_, 4a (** Scheme 7 **)**

From **1c** and propyne (THF solution, ca. 1 mol/L). Brown solid, yield 90%. Eluent for chromatography: MeCN. Anal. calcd. for C_24_H_28_F_3_Fe_2_NO_5_S: C, 47.16; H, 4.62; N, 2.29; S, 5.25. Found: C, 47.02; H, 4.70; N, 2.23; S, 5.31. IR (CH_2_Cl_2_): ῦ/cm^−1^ = 1989vs (CO), 1805s (μ-CO), 1661m (C^1^ = N). ^1^H NMR (acetone-d_6_): δ/ppm = *5.53*, 5.52, *5.20*, 5.17 (s, 10 H, Cp); 4.81, *3.60* (m, 1 H, CH^Cy^); *4.74*, 4.69 (s, 1 H, C^2^H); *3.98*, 3.96 (s, 3 H, C^3^Me); *3.90*, 3.20 (s, 3 H, NMe); 2.26, 2.03–1.51, 1.43–1.06 (m, 10 H, CH_2_^Cy^). Isomer ratio (E/Z) = 1.2. ^13^C{^1^H} NMR (acetone-d_6_): δ/ppm = 257.3, *256.4* (μ-CO); *225.5*, 224.6 (C^1^); *210.9*, 210.7 (CO); *208.2*, 207.9 (CO); 204.9 (C^3^); 90.7, *87.7*, 87.5 (Cp); *75.2*, 68.2 (CH^Cy^); 52.0, *51.3* (C^2^); 42.9, 38.2 (NMe); *41.2*, 41.1 (C^3^*Me*); 29.9, 29.8, 29.7, 25.1, 24.7, 24.6, 24.5 (CH_2_^Cy^).

**[Fe_2_Cp_2_(CO)(μ-CO){μ-η^1^:η^3^-C^3^(Ph)C^2^HC^1^NMe(Cy)}]CF_3_SO_3_, 4b (** Scheme 8 **)**

From **1c** and phenylacetylene. Brownish-green solid, yield 93%. Eluent for chromatography: MeCN. Anal. calcd. for C_29_H_30_F_3_Fe_2_NO_5_S: C, 51.73; H, 4.49; N, 2.08; S, 4.76. Found: C, 51.68; H, 4.53; N, 2.12; S, 4.69. IR (CH_2_Cl_2_): ῦ/cm^−1^ = 1992vs (CO), 1809s (μ-CO), 1658m (C^1^ = N). ^1^H NMR (acetone-d_6_): δ/ppm = 7.83, 7.57, 7.44 (m, 5 H, Ph); *5.44*, 5.40, *5.28*, 5.27 (s, 10 H, Cp); 4.95, *3.83* (m, 1 H, CH^Cy^); *4.77*, 4.73 (s, 1 H, C^2^H); *4.02*, 3.35 (s, 3 H, NMe); 2.3–1.1 (m, 10 H, CH_2_^Cy^). Isomer ratio (E/Z) = 1.2. ^13^C{^1^H} NMR (acetone-d_6_): δ/ppm = 256.7, *255.7* (μ-CO); *224.3*, 223.6 (C^1^); *210.3*, 210.1 (CO); *204.5*, 204.1 (C^3^); 156.4 (*ipso*-Ph); 128.3, 127.4, 127.0 (Ph); 91.7, *87.9*, 87.7 (Cp); *75.5*, 68.0 (CH^Cy^); 52.4, *51.8* (C^2^); 43.2, *38.1* (NMe); 29.9, 29.8, 25.2, 24.7–24.5 (CH_2_^Cy^).

**[Fe_2_Cp_2_(CO)(μ-CO){μ-η^1^:η^3^-C^3^(Me)C^2^(Me)C^1^NMe(Cy)}]CF_3_SO_3_, 4c (** Scheme 9 **)**

From **1c** and 2-butyne. Brownish-green solid, yield 95%. Eluent for chromatography: MeCN. Anal. calcd. for C_25_H_30_F_3_Fe_2_NO_5_S: C, 48.02; H, 4.84; N, 2.24; S, 5.13. Found: C, 47.89; H, 4.90; N, 2.18; S, 5.19. IR (CH_2_Cl_2_): ῦ/cm^−1^ = 1986vs (CO), 1805s (μ-CO), 1646m (C^1^ = N). ^1^H NMR (acetone-d_6_): δ/ppm = 5.51, 5.20, *5.15* (s, 10 H, Cp); *4.86*, 3.51 (br, 1 H, CH^Cy^); 3.93, *3.16* (s, 3 H, NMe); 3.91 (s, 3 H, C^3^Me); 2.2, 1.9–1.1 (m, 10 H, CH_2_^Cy^); 2.04, *2.02* (s, 3 H, C^2^Me). Isomer ratio (Z/E) = 2. ^13^C{^1^H} NMR (acetone-d_6_): δ/ppm = *258.3*, 257.5 (μ-CO); 226.3, *224.4* (C^1^); 211.3, *210.7* (CO); 202.2, *201.9* (C^3^); 91.1, 88.2, *88.0* (Cp); 73.9, *68.0* (CH^Cy^); *64.8*, 64.2 (C^2^); 40.9, *36.4* (NMe); 37.3, *36.2* (C^3^*Me*); 30.4, 30.0, 25.3, 24.7, 24.6, 24.5 (CH_2_^Cy^); 15.7, *15.0* (C^2^*Me*).

**[Fe_2_Cp_2_(CO)(μ-CO){μ-η^1^:η^3^-C^3^(Me)C^2^HC^1^NMe(CH_2_Ph)}]CF_3_SO_3_, 5a (** Scheme 10 **)**

From **1d** and propyne (THF solution, ca. 1 mol/L). Brown solid, yield 88%. Eluent for chromatography: MeCN. Anal. calcd. for C_25_H_24_F_3_Fe_2_NO_5_S: C, 48.49; H, 3.91; N, 2.26; S, 5.18. Found: C, 48.58; H, 3.85; N, 2.21; S, 5.11. IR (CH_2_Cl_2_): ῦ/cm^−1^ = 1991vs (CO), 1808s (μ-CO), 1664m (C^1^ = N). ^1^H NMR (acetone-d_6_): δ/ppm = 7.39–7.27, 7.07 (m, 5 H, Ph); *5.49*, *5.31*, 4.80, 4.42 (d, 2 H, ^2^J_HH_ = 14.2 Hz, CH_2_); 5.25, 5.01 (s, 10 H, Cp); *4.74*, 4.65 (s, 1 H, C^2^H); 3.88 (s, 3 H, C^3^Me); 3.62, 3.00 (s, 3 H, NMe). Isomer ratio (E/Z) = 1.2. ^13^C{^1^H} NMR (acetone-d_6_): δ/ppm = 256.8 (μ-CO); 227.5, 226.7 (C^1^); *210.2*, 210.0 (CO); 209.1, *208.8* (C^3^); 132.4, 131.7, 129.4, 129.3, 129.2, 129.1 (Ph); 90.5, *90.4*, 87.9, *87.8* (Cp); *68.4*, 61.9 (CH_2_); *52.1*, 52.6 (C^2^); 47.9, 42.1 (NMe); 42.4, *42.3* (C^3^*Me*).

**[Fe_2_Cp_2_(CO)(μ-CO){μ-η^1^:η^3^-C^3^(Me)C^2^(Me)C^1^NMe(CH_2_Ph)}]CF_3_SO_3_, 5b (** Scheme 11 **)**

From **1d** and 2-butyne. Brown solid, yield 70%. Eluent for chromatography: MeCN. Anal. calcd. for C_26_H_26_F_3_Fe_2_NO_5_S: C, 49.31; H, 4.14; N, 2.21; S, 5.06. Found: C, 49.20; H, 4.19; N, 2.16; S, 5.09. IR (CH_2_Cl_2_): ῦ/cm^−1^ = 1986vs (CO), 1805s (μ-CO), 1650m (C^1^ = N). ^1^H NMR (acetone-d_6_): δ/ppm = 7.49–7.44, 7.32 (m, 5 H, Ph); *5.57*, 5.55, 5.25, *5.23* (s, 10 H, Cp); 4.87, 4.57 (d, ^2^J_HH_ = 14.7 Hz, 2 H, CH_2_); 3.95 (s, 3 H, C^3^Me); 3.81, *3.06* (s, 3 H, NMe); 2.14 (s, 3 H, C^2^Me). Isomer ratio (Z/E) = 1.5. ^13^C{^1^H} NMR (acetone-d_6_): δ/ppm = 258.3 (μ-CO); 228.5, 226.9 (C^1^); 211.0, *210.7* (CO); *202.6*, 202.2 (C^3^); 133.4, 132.4 (*ipso*-Ph); 129.2, 129.1, 129.0, 128.9, 128.8, 128.7 (Ph); 91.2, 88.3, *88.2* (Cp); 65.9, *61.3* (C^2^); *65.6*, 65.5 (CH_2_); 45.4, 41.6 (NMe); 36.4, *36.3* (C^3^*Me*); 15.5, *15.0* (C^2^*Me*).

**[Fe_2_Cp_2_(CO)(μ-CO){μ-η^1^:η^3^-C^3^(Et)C^2^(Et)C^1^NMe(CH_2_Ph)}]CF_3_SO_3_, 5c (** Scheme 12 **)**

From **1d** and 3-hexyne. Red-brown solid, yield 69%. Eluent for chromatography: MeCN. Anal. calcd. for C_28_H_30_F_3_Fe_2_NO_5_S: C, 50.85; H, 4.57; N, 2.12; S, 4.85. Found: C, 50.78; H, 4.64; N, 2.09; S, 4.94. IR (CH_2_Cl_2_): ῦ/cm^−1^ = 1986vs (CO), 1804s (μ-CO), 1649m (C^1^ = N). ^1^H NMR (CDCl_3_): δ/ppm = 7.41–7.29, 7.08 (m, 5 H, Ph); *5.59*, *5.40*, 4.85, 4.35 (d, ^2^J_HH_ ≈ 14 Hz, 2 H, NCH_2_); 5.28, 5.02 (s, 10 H, Cp); 4.19, 4.09, 2.53, 2.28, 2.07 (br, 4 H, C*H*_2_CH_3_); 3.69, *2.90* (s, 3 H, NMe); 1.95, 1.72, 1.44, 1.28, 1.14 (br, 6 H, CH_2_C*H*_3_). Isomer ratio (trans-Z/cis-E) = 1.8. ^13^C{^1^H} NMR (CDCl_3_): δ/ppm = *258.9*, 257.8 (μ-CO); 228.3, *226.7* (C^1^); 211.9, *211.4* (CO); 210.9, *210.3* (C^3^); 132.4, 131.3, 129.4, 129.3, 129.2, 129.1, 128.9 (Ph); *90.7*, 90.5, 88.0, *87.8* (Cp); 68.5, 61.8 (NCH_2_); 66.9 (C^2^); 46.4, 42.1 (NMe); *43.2*, 42.9 (C^3^-*C*H_2_); 24.1, *23.0* (C^2^-*C*H_2_); 20.2, *19.3*, 14.9, *13.4* (CH_2_*C*H_3_).

**[Fe_2_Cp_2_(CO)(μ-CO){μ-η^1^:η^3^-C^3^(Me)C^2^HC^1^NMe(4-C_6_H_4_OMe)}]CF_3_SO_3_, 6a (**Scheme 13**)** [52]

From **1e** and propyne (THF solution, ca. 1 mol/L). Brown solid, yield 73%. Eluent for chromatography: MeCN. Anal. calcd. for C_25_H_24_F_3_Fe_2_NO_6_S: C, 47.27; H, 3.81; N, 2.21; S, 5.05. Found: C, 47.13; H, 3.91; N, 2.13; S, 5.09. IR (CH_2_Cl_2_): ῦ/cm^−1^ = 1991vs (CO), 1811s (μ-CO), 1642m (C^1^ = N). ^1^H NMR (acetone-d_6_): δ/ppm = 7.64, 7.24, 7.20, 6.90 (m, 4 H, C_6_H_4_); 5.60, *5.55*, 5.32, *4.99* (s, 10 H, Cp); *4.85*, 4.72 (s, 1 H, C^2^H); *4.41*, 3.69 (s, 3 H, NMe); 4.02, 3.95 (s, 3 H, C^3^Me); 3.81 (s, 3 H, OMe). Isomer ratio (E/Z) = 2. ^13^C{^1^H} NMR (acetone-d_6_): δ/ppm = 255.6, *254.8* (μ-CO); 230.4, *229.0* (C^1^); *210.7*, 210.4 (CO); *209.5*, 208.8 (C^3^); *160.3*, 159.8 (*ipso*-C_6_H_4_); 139.4, *136.5*, 126.1, 122.8, *114.6*, 114.3 (C_6_H_4_); 91.0, 88.1, *87.8* (Cp); *55.2*, 55.1 (OMe); 53.7, *53.4* (C^2^); *53.6*, 46.2 (NMe); 41.4, *41.3* (C^3^*Me*).

**[Fe_2_Cp_2_(CO)(μ-CO){μ-η^1^:η^3^-C^3^(Ph)C^2^HC^1^NMe(4-C_6_H_4_OMe)}]CF_3_SO_3_, 6b (** Scheme 14 **)**

From **1e** and phenylacetylene. Brown solid, yield 71%. Eluent for chromatography: MeCN. Anal. calcd. for C_30_H_26_F_3_Fe_2_NO_6_S: C, 51.68; H, 3.76; N, 2.01; S, 4.60. Found: C, 51.55; H, 3.88; N, 2.06; S, 4.71. IR (CH_2_Cl_2_): ῦ/cm^−1^ = 1994vs (CO), 1814s (μ-CO), 1639m (C^1^ = N). ^1^H NMR (acetone-d_6_): δ/ppm = 7.84, 7.68, 7.56, 7.43, 7.28, 6.98 (m, 9 H, Ph + C_6_H_4_); 5.57, 5.33, *5.27*, *5.18* (s, 10 H, Cp); *4.93*, 4.72 (s, 1 H, C^2^H); *4.51*, 3.80 (s, 3 H, NMe); *3.99,* 3.83 (s, 3 H, OMe). Isomer ratio (E/Z) = 4. ^13^C{^1^H} NMR (acetone-d_6_): δ/ppm = 254.7 (μ-CO); 229.2 (C^1^); 210.0 (CO); 204.9 (C^3^); 159.9 (*ipso*-C_6_H_4_); 156.4 (*ipso*-Ph); *128.8*, 128.4, 127.2, 127.0, 126.9, 126.5, 122.7, *114.7*, 114.4 (Ph + C_6_H_4_); 92.0, *91.9*, 88.3, *87.9* (Cp); *55.3*, 55.2 (OMe); 54.2, *54.0* (C^2^); 54.3, 46.0 (NMe).

**[Fe_2_Cp_2_(CO)(μ-CO){μ-η^1^:η^3^-C^3^(Me)C^2^(Me)C^1^NMe(4-C_6_H_4_OMe)}]CF_3_SO_3_, 6c (** Scheme 15 **)**

From **1e** and 2-butyne. Brown solid, yield 83%. Eluent for chromatography: MeCN. Anal. calcd. for C_30_H_26_F_3_Fe_2_NO_6_S: C, 51.68; H, 3.76; N, 2.01; S, 4.60. Found: C, 51.55; H, 3.88; N, 2.06; S, 4.71. IR (CH_2_Cl_2_): ῦ/cm^−1^ = 1986vs (CO), 1810s (μ-CO), 1626m (C^1^ = N). ^1^H NMR (acetone-d_6_): δ/ppm = 7.59, 7.20, 7.10, 6.92 (m, 4 H, C_6_H_4_); 5.57, *5.45*, 5.25, *4.96* (s, 10 H, Cp); 4.44, *3.56* (s, 3 H, NMe); *3.93*, 3.78 (s, 3 H, OMe); *3.89*, 3.82 (s, 3 H, C^3^Me); *2.26*, 1.50 (s, 3 H, C^2^Me). Isomer ratio (Z/E) = 2.2. ^13^C{^1^H} NMR (acetone-d_6_): δ/ppm = 257.3, 255.4 (μ-CO); 229.9, *229.1* (C^1^); 212.3, *211.8* (CO); 204.2, *203.6* (C^3^); *161.3*, 160.7 (*ipso*-C_6_H_4_); 138.4, *138.2*; *127.7*, 123.0, *115.4*, 115.1 (C_6_H_4_); 92.5, *92.2*, 89.6, *89.2* (Cp); 67.8, *67.2* (C^2^); *56.2*, 56.1 (OMe); 52.7, 46.5 (NMe); 37.9, *37.2* (C^3^*Me*); *15.8*, 15.3 (C^2^*Me*).

**[Fe_2_Cp_2_(CO)(μ-CO){μ-η^1^:η^3^-C^3^(Me)C^2^(Me)C^1^NMe(2-naphthyl)}]CF_3_SO_3_, 7 (** Scheme 16 **)**

From **1f** and 2-butyne. Brown-red solid, yield 90%. Eluent for chromatography: MeCN. Anal. calcd. for C_29_H_26_F_3_Fe_2_NO_5_S: C, 52.04; H, 3.92; N, 2.09; S, 4.79. Found: C, 51.89; H, 3.99; N, 2.01; S, 4.94. IR (CH_2_Cl_2_): ῦ/cm^−1^ = 1987vs (CO), 1811s (μ-CO), 1637m (C^1^ = N), 1616w-m (arom C = C). ^1^H NMR (acetone-d_6_): δ/ppm = 8.32–7.61, 7.28 (m, 7 H, arom CH); 5.67, *5.51*, 5.36, *5.01* (s, 10 H, Cp); 4.67, *3.75* (s, 3 H, NMe); *3.97*, 3.93 (s, 3 H, C^3^Me); *2.26*, 1.55 (s, 3 H, C^2^Me). Isomer ratio (Z/E) = 1.5. ^13^C{^1^H} NMR (acetone-d_6_): δ/ppm = 256.0, *254.5* (μ-CO); 230.9 (C^1^); 211.4 (CO); 203.9 (C^3^); 141.0 (*ipso*-Naph); 133.4, 132.7, 132.6, 129.7, 129.4, 128.5, 128.4, 128.1, 127.7, 127.5, 124.6, 122.8, 118.9 (arom CH); 91.7, *91.3*, 88.9, *88.3* (Cp); 67.1 (C^2^); 51.4, 45.6 (NMe); 37.1, *36.4* (C^3^*Me*); *15.0*, 14.5 (C^2^*Me*).

### 2.3. X-ray Crystallography

Crystal data and collection details for **2c** and **3a** are reported in Table 1. The data were recorded on a Bruker APEX II diffractometer equipped with a PHOTON100 detector using Mo–Kα radiation. The data were corrected for Lorentz polarization and absorption effects (empirical absorption correction SADABS) [54]. The structures were solved by direct methods and refined by full-matrix least-squares based on all data using *F*^2^ [55]. Hydrogen atoms were fixed at calculated positions and refined by a riding model. All non-hydrogen atoms were refined with anisotropic displacement parameters. The crystals of **2c** appeared to be non-merohedrally twinned. The TwinRotMat routine of PLATON [56] was used to determine the twinning matrix and to write the reflection data file (.hkl) containing the twin components. Refinement was performed using the instruction HKLF 5 in SHELXL and one BASF parameter, which refined as 0.213 (5). Moreover, the whole molecule **2c** is disordered and, therefore, it has been split into two positions and refined anisotropically employing one occupancy factor per disordered group. Because of this twinning and high disorder, several restraints were used during the refinement. The details are included in the CIF file. Therefore, even if the connectivity and overall geometry of **2c** are certain, bonding distances and angles must be taken with care.

### 2.4. Solubility and Stability in Water and Determination of Octanol–Water Partition Coefficients

(a) Solubility in D_2_O. A suspension of the selected diiron compound (3–5 mg) in a D_2_O solution (ca. 1 mL) containing Me_2_SO_2_ (3.36 × 10^−3^ M) as internal standard [57] was vigorously stirred at 21 °C for 1.5 h. The resulting saturated solution was filtered over celite, transferred into an NMR tube and then analyzed by ^1^H NMR spectroscopy (delay time = 3 s; number of scans = 20). The concentration (solubility) was calculated by the relative integral (related to Cp and/or NMe signals) with respect to Me_2_SO_2_ (δ = 3.14 ppm).
(b) Stability in DMSO-d_6_/D_2_O solution. The selected diiron compound (*ca*. 4 mg) was added of 1 mL of D_2_O (**3a**,**c**) or DMSO-d_6_/D_2_O (2:1 *v/v*) mixture (**2a,c**, **3b**, **4a–c**, **5a–c**, **6a–c**, **7**), containing Me_2_SO_2_ (3.36 × 10^−3^ M). The resulting mixture was stirred for 30 min and then filtered over celite; the filtrated solution was transferred into an NMR tube. The sample was analyzed by ^1^H NMR (time = 0) and then heated at 37 °C for 72 h. After cooling to room temperature, the solution was separated from a small amount of solid by filtration over celite, and the new ^1^H NMR spectrum was recorded (delay time = 3 s; number of scans = 20). In every case, no newly formed organometallic species was detected. The amount of starting material in solution (% with respect to the initial spectrum) was calculated by the relative integral (vs. Cp and/or NMe signals) with respect to Me_2_SO_2_ as internal standard. Data related to the spectra recorded at time = 0 are reported in the following.

**2a.**^1^H NMR (D_2_O/DMSO-d_6_ 2:1): δ/ppm = 7.43, 7.15, 6.99 (m, 10 H, Ph); 5.83, 5.14 (d, 2 H, CH_2_); 5.48, 5.08 (s, 10 H, Cp); 4.54, 4.27 (d, 2 H, CH_2_); 3.87 (s, 3 H, C^3^Me).

**2c.**^1^H NMR (D_2_O/DMSO-d_6_ 2:1): δ/ppm = 7.4–6.9 (m, 10 H, Ph); 4.73 (d, 1 H, CH_2_); 5.42, 5.01 (s, 10 H, Cp); 3.82 (s, 3 H, C^3^Me); 2.17 (s, 3 H, C^2^Me).

**3a.**^1^H NMR (D_2_O): δ/ppm = 5.92–5.82, 5.62–5.54, 5.38–5.33 (m, CH = CH_2_); 5.29, *5.29*, 4.92, *4.90* (s, Cp); *3.68*, 3.67 (s, C^3^Me); 3.65, *3.01* (NMe). Isomer ratio = 1.1.

**3b.**^1^H NMR (D_2_O/DMSO-d_6_ 2:1): δ/ppm = 7.73, 7.59, 7.47 (m, 5 H, Ph); 5.97 (br, C*H* = CH_2_); 5.65, 5.54, 5.35, 5.31 (m, CH = C*H*_2_); 5.27, *5.25*, 5.15 (s, 10 H, Cp); 4.12 (m, NCH_2_); 3.85, *3.23* (s, 3 H, NMe). Isomer ratio = 1.5.

**3c.**^1^H NMR (D_2_O): δ/ppm = 5.99, 5.62, 5.52, 5.36 (m, CH = CH_2_); 5.26, *5.24*, 4.92, 4.90 (s, Cp); 3.68, 3.65, 3.05 (s, C^3^Me + NMe); 2.04 (s, C^2^Me).

**4a.**^1^H NMR (D_2_O/DMSO-d_6_ 2:1): δ/ppm = 5.35, *5.34*, 5.17, *4.97* (s, 10 H, Cp); 3.77, 3.75 (s, 3 H, C^3^Me); *3.73*, 3.00 (s, 3 H, NMe); *3.43* (m, 1 H, CH^Cy^); 2.08–0.85 (m, 10 H, CH_2_^Cy^). Isomer ratio = 1.9.

**4c.**^1^H NMR (D_2_O/DMSO-d_6_ 2:1): δ/ppm = 5.35, *5.34*, 5.01, *4.97* (s, 10 H, Cp); 3.77, 3.75, 3.73 (s, NMe + C^3^Me); 2.1, 1.8–1.1 (m, 10 H, CH_2_^Cy^); 1.87, *1.85* (s, 3 H, C^2^Me). Isomer ratio = 1.8.

**5a.**^1^H NMR (D_2_O/DMSO-d_6_ 2:1): δ/ppm = 7.48, 7.36, 7.21 (m, 5 H, Ph); *5.45*, 5.43, 5.07 (s, 10 H, Cp); 3.88 (s, 3 H, C^3^Me); 3.61, *3.03* (s, 3 H, NMe). Isomer ratio = 1.2.

**5b.**^1^H NMR (D_2_O/DMSO-d_6_ 2:1): δ/ppm = 7.49–7.36, 7.19 (m, 5 H, Ph); *5.40*, 5.39, 5.06, *5.01* (s, 10 H, Cp); 4.50 (d, 1 H, CH_2_); 3.79 (s, 3 H, C^3^Me); 3.65, *3.06* (s, 3 H, NMe); 1.94, *1.91* (C^2^Me). Isomer ratio = 1.7.

**5c.**^1^H NMR (D_2_O/DMSO-d_6_ 2:1): δ/ppm = 7.51, 7.43, 7.14 (m, 5 H, Ph); *5.53*, *5.30*, 4.51 (d, 2 H, NCH_2_); 5.42, *5.40*, 5.12, *5.09* (s, 10 H, Cp); 4.18, 4.13, 2.46, 2.13 (br, 4 H, *CH*_2_CH_3_); 3.72, *2.94* (s, NMe); 2.11, 1.69, 1.13, 0.98 (br, CH_2_*CH*_3_). Isomer ratio = 1.7.

**6a.**^1^H NMR (D_2_O/DMSO-d_6_ 2:1): δ/ppm = 7.52, 7.21, 7.10, 6.93 (m, 4 H, C_6_H_4_); 5.43, *5.37*, 5.14, *4.85* (s, 10 H, Cp); 4.63 (s, 1 H, C^2^H); 4.41, *3.69* (s, 3 H, NMe); 4.23, *3.93* (s, 3 H, C^3^Me); *3.87*, 3.79 (s, 3 H, OMe). Isomer ratio = 2.

**6b.**^1^H NMR (D_2_O/DMSO-d_6_ 2:1): δ/ppm = 7.73, 7.58, 7.43, 7.23, 7.16, 6.98 (m, 9 H, Ph + C_6_H_4_); 5.37, 5.19, *5.12*, *5.03* (s, 10 H, Cp); 4.32, *3.96* (s, 3 H, NMe); 3.79, *3.64* (s, 3 H, OMe). Isomer ratio = 3.

**6c.**^1^H NMR (D_2_O/DMSO-d_6_ 2:1): δ/ppm = 7.94, 7.21, 7.04, 6.94 (m, 4 H, C_6_H_4_); 5.45, *5.32*, 5.10, *4.84* (s, 10 H, Cp); 4.30, *3.46* (s, 3 H, NMe); *3.93*, 3.78 (s, 3 H, OMe); 3.74 (s, 3 H, C^3^Me); *2.00*, 1.44 (s, 3 H, C^2^Me). Isomer ratio = 2.3.

**7.**^1^H NMR (D_2_O/DMSO-d_6_ 2:1): δ/ppm = 8.23–7.21 (m, 7 H, arom CH); 5.51, *5.34*, 5.16, *4.86* (s, 10 H, Cp); 4.47, *3.61* (s, 3 H, NMe); *3.82*, 3.78 (s, 3 H, C^3^Me); *2.09*, 1.44 (s, 3 H, C^2^Me). Isomer ratio = 2.

(c) Octanol–water partition coefficients (Log *P*_ow_). Partition coefficients (*P*_ow_; IUPAC: *K*_D_ partition constant [58]), defined as *P*_ow_ = c_org_/c_aq_, where c_org_ and c_aq_ are molar concentrations of the selected compound in the organic and aqueous phase, respectively, were determined by the shake-flask method and UV–Vis measurements [36,59,60]. Deionized water and 1-octanol were vigorously stirred for 24 h to enable saturation of both phases, then separated by centrifugation. A stock solution of the selected diiron compound (*ca.* 2 mg; **2a–c**, **3b**, **4a–c**, **5a**, **5c**, **6b,c**, **7**) was prepared by first adding acetone (50 μL, to help solubilization), followed by water-saturated octanol (2.5 mL). The solution was diluted with water-saturated octanol (*ca.* 1:3 *v*/*v* ratio, c_Fe2_ ≈ 10^−4^ M, so that 1.5 ≤ A ≤ 2.0 at λ_max_) and the UV–Vis spectrum was recorded (A^0^_org_). An aliquot of the solution (V_org_ = 1.2 mL) was transferred into a test tube and octanol-saturated water (V_org_ = V_aq_ = 1.2 mL) was added. The mixture was vigorously stirred for 15 min at 21 °C and then centrifuged (5000 rpm, 10 min). The UV–Vis spectrum of the organic phase was recorded (A^f^_org_) and the partition coefficient was calculated as *P*_ow_ = A^f^_org_/(A^0^_org_ − A^f^_org_), where A^0^_org_ and A^f^_org_ are the absorbance in the organic phase before and after partition with the aqueous phase, respectively [59]. An inverse procedure was followed for **3a**, **3c**, **5b** and **6a**, starting from a solution of the compound in octanol-saturated water. The partition coefficient was calculated as *P*_ow_ = (A^0^_aq_ − A^f^_aq_)/A^f^_aq_ where A^0^_aq_ and A^f^_aq_ are the absorbance in the aqueous phase before and after partition with the organic phase, respectively. The wavelength of the maximum absorption of each compound (ca. 300 nm) was used for UV–Vis quantitation. The procedure was repeated three times for each sample (from the same stock solution); results are given as mean ± standard deviation. Naphthoquinone was used as a reference compound (Log *P* = 1.8 ± 0.2; literature [61]: 1.71).

### 2.5. Cell Culture and Cytotoxicity Studies

In vitro cytotoxicity investigations were carried out by using human ovarian carcinoma cisplatin-sensitive A2780 (ECACC93112519), human ovarian carcinoma cisplatin-resistant A2780cisR (ECACC 93112517) and mouse embryo fibroblasts Balb/3T3 clone A31 (ATCC CCL-163) cell lines. A2780 and A2780cisR were purchased from the European Collection of Authenticated Cell Cultures (ECACC), and Balb/3T3 clone A31 cell line from the American Type Culture Collection (ATCC).

A2780 and A2780cisR cells were routinely cultured in RPMI 1640 (Merck) containing 2 mM of L-glutamine (Merck), 1% of penicillin/streptomycin solution (Merck—10 000 U mL^−1^: 10 mg mL^−1^), 10% of fetal bovine serum (Merck—FBS) and antimycotic (InvivoGen, USA), and Balb/3T3 clone A31 in Dulbecco’s modified Eagle medium (Merck—DMEM) supplemented with 4 mM of L-glutamine, 1% of penicillin/streptomycin solution, 10% of calf serum (Merck) and antimycotic. The acquired resistance of A2780cisR cells was maintained by routine supplementation of media with 1 μM of cisplatin (Merck). The cultures were maintained at 37 °C and in a 5% CO_2_-enriched atmosphere.

A2780, A2780cisR and Balb/3T3 clone A31 cells were seeded in 96-well tissue culture polystyrene plates at a concentration of 3 × 10^3^, 6 × 10^3^ and 1 × 10^3^ cells per well, respectively. After overnight incubation, the cells were treated with different concentrations (0–100 µM) of the selected compounds for 72 h at 37 °C in a 5% atmosphere of CO_2_. Stock solutions of compounds were prepared in DMSO and sequentially diluted in medium (final DMSO concentration of 0.5%). Cells incubated with cisplatin (0−100 μM) were used as positive control. At the end of the incubation time, cell viability was assessed by means of WST-1 tetrazolium salt reagent (Roche). Briefly, cells were incubated for 4 h with the tetrazolium salt reagent diluted 1:10, at 37 °C and 5% CO_2_. Measurements of formazan dye absorbance, which directly correlates with the number of viable cells, were carried out with a micro-plate reader (Biorad) at 450 nm, using 655 nm as reference wavelength. The 50% inhibitory concentration (IC_50_) refers to compound concentration at which 50% of cell death is observed with respect to the control. For each tested compound, assay was performed on triplicate. The concentration effect curves were generated by nonlinear regression curves (GraphPad Prism Software, **2021**) and the data reported as mean ± standard deviation.

### 2.6. ROS Determination

The intracellular production of reactive oxygen species (ROS) upon treatment of the compounds **3b**, **4a**, **4c**, **5b** and **6c** was measured by using the DCFH-DA (2′,7′-dichlorodihydrofluorescein diacetate, Merck) assay, based on cellular uptake of the non-fluorescent diacetate following deacetylation by esterases (2′,7′-dichlorodihydrofluorescein, DCFH) and oxidation to the fluorescent dichlorofluorescein (2′,7′-dichlorofluorescein, DCF) [62]. A2780 cells were seeded at concentration of 4 × 10^4^ cells/well/90 µL of complete growth medium into 96-well plates. After overnight incubation, the cells were treated following manufacturer protocol. 100 µL of a solution containing the fluorogenic probe were added to the culture medium and, after 1 h of incubation with 5% CO_2_ at 37 °C, the cells were exposed to a final concentration of 20 µM of the tested complexes; H_2_O_2_ 100 µM was used as a positive control. Stock solutions of compounds were prepared as described above; cells incubated with equal amounts of DMSO in supplemented RPMI were used as control. The fluorescence was measured up to 24 h with an excitation wavelength of 485 nm and with a 535 nm emission filter by Multilabel Counter (PerkinElmer). For each tested compound, assay was performed on triplicate. The data were reported as mean ± standard deviation, statistical differences were analyzed using one-way analysis of variance (ANOVA) and a Tukey test was used for post hoc analysis. A *p*-value *<* 0.05 is considered statistically significant.

### 2.7. Biomolecules Binding Studies

(a) Sample preparation. Cytochrome c (Cyt c) was commercially available and used as received; the TrxR dodecapeptide (**TrxR-pept**) was synthesized as reported in the literature [63,64]. The stock solutions of the selected iron-based complexes were prepared in DMSO up to a final concentration of 10^−2^ M. The stock solution of **TrxR-pept** was prepared in LC-MS grade water by dissolving the required amount of lyophilized peptide to reach a final concentration of 10^−3^ M. The stock solution of Cyt c 10^−3^ M was prepared by dissolving the required amount of protein in 2 mM ammonium acetate solution at pH 6.8. In the interaction tests with **TrxR-pept**, opportune aliquots of each complex and **TrxR-pept** stock solutions were mixed and diluted with LC-MS grade water to 10^−4^ M final concentration and a **TrxR-pept**/complex ratio of 1:1. For each iron-based complex/Cyt c pair, appropriate aliquots of the respective stock solutions were mixed and subsequently diluted with 2 mM ammonium acetate solution (pH 6.8) to a final protein concentration of 10^−4^ M and a protein-to-metal molar ratio of 1:2. All the sample mixtures were incubated for 24 h at 37 °C. Subsequently, opportune dilutions were performed as detailed:
  – the **TrxR-pept** containing solutions were further diluted with LC-MS grade water to a final **TrxR-pept** concentration of 10^−5^ M and added with 0.1% *v*/*v* of formic acid just before infusion in the mass spectrometer;
  – the protein-containing solutions were diluted with 2 mM ammonium acetate solution (pH 6.8) to a final protein concentration of 10^−6^ M and added with 0.1% *v*/*v* of formic acid just before infusion.
(b) ESI-MS instrumental parameters. The ESI mass spectra were acquired using a TripleTOF^®^ 5600^+^ high-resolution mass spectrometer (Sciex, Framingham, MA, USA), with a DuoSpray^®^ interface operating with an ESI probe. Respective ESI mass spectra were acquired through direct infusion at 7 μL min^−1^ flow rate. The ESI source parameters were optimized for each biomolecule and were as follows: for TrxR dodecapeptide positive polarity, ionspray voltage floating 5500 V, temperature 0, ion source gas 1 (GS1) 35 L min^−1^; ion source gas 2 (GS2) 0; curtain gas (CUR) 20 L min^−1^, declustering potential (DP) 300 V, collision energy (CE) 10 V, range 1070–1600 *m/z*; for Cyt c positive polarity, ionspray voltage floating 5500 V, temperature 0, ion source gas 1 (GS1) 35 L min^−1^; ion source gas 2 (GS2) 0; curtain gas (CUR) 20 L min^−1^, declustering potential (DP) 180 V, collision energy (CE) 10 V, range 500–1800 *m/z*. For acquisition, Analyst TF software 1.7.1 (Sciex, Framingham, MA, USA) was used and deconvoluted spectra were obtained by using the Bio Tool Kit micro-application v.2.2 embedded in PeakView^TM^ software v.2.2 (Sciex, Framingham, MA, USA).

## 3. Results and Discussion

### 3.1. Synthesis and Structural Characterization of Diiron Complexes

**Scheme 17:**
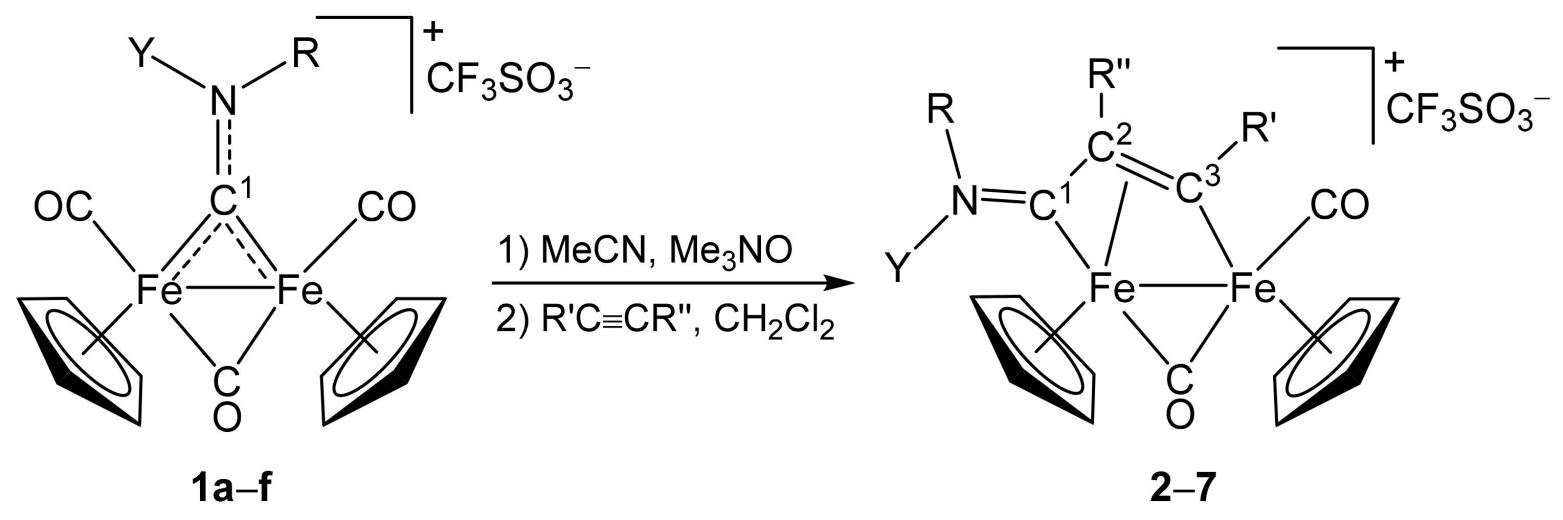
General synthetic pathway to diiron vinyliminium complexes (see Table 2 for details).

A series of diiron complexes with novel vinyliminium ligands, **2–7**, was synthesized from the respective aminocarbyne precursors, **1a–f**, by means of a two-step procedure (see Scheme 17 and Table 2). This consists of the preliminary CO-NCMe substitution promoted by trimethylamine-N-oxide, followed by replacement of the labile acetonitrile ligand with the appropriate alkyne in dichloromethane solution at room temperature. Three main alkynes were selected, i.e., propyne, phenylacetylene and 2-butyne, which previously demonstrated to provide enhanced cytotoxicity to other vinyliminium complexes (Figure 1) [39]. In addition, 3-hexyne was employed to build **5c**, in order to outline the possible effect of the length of alkyl chains (upon comparison with **5b**) [39]. Note that **1a–f** are available from [Fe_2_Cp_2_(CO)_4_] in high yields via multigram scale synthesis [36,47].

The products **2–7** were purified by alumina chromatography and finally isolated as air-stable triflate salts in 69–95% yields. They were fully characterized by elemental analysis, IR and NMR spectroscopy (Figures S1–S32). The IR spectra (in CH_2_Cl_2_ solution) share a common pattern with three main bands; two of them are ascribable to the terminal and bridging carbonyl stretching vibrations and fall in the ranges 1986–1992 cm^−1^ and 1804–1814 cm^−1^, respectively. The third band is related to the iminium (C^1^ = N) function and reveals a double bond character; however, its wavenumber is rather sensitive to the electronic properties of all vinyliminium substituents (i.e., R, Y, R′ and R″). For instance, it occurs at 1644 and 1664 cm^−1^ in **2a** and **5a**, respectively, the latter complex differing from the former for a CH_3_ group instead of CH_2_Ph on nitrogen. The substitution of the C^2^ carbon determines a significant decrease of the C^1^ = N wavenumber, as well as a minor decrease for the CO signal [e.g., for **4a**: ῦ/cm^−1^ = 1989 (CO), 1805 (μ-CO), 1661 (C^1^ = N); for **4c**: ῦ/cm^−1^ = 1986 (CO), 1805 (μ-CO), 1646 (C^1^ = N)]. The allyl moiety in **3a–c** manifests itself with a weak infrared absorption at ca. 1640 cm^−1^ (double carbon–carbon bond). The NMR spectra of **2a–c** (in acetone-d_6_) show a single set of resonances, and in particular the singlets due to the Cp rings, in the ^1^H spectra, have been detected at ca. 5.6 and 5.3 ppm, pointing out their mutual cis arrangement. In fact, cis/trans isomerism was previously recognized in some analogous complexes, the trans form being associated to a diagnostic upfield shift of one Cp resonance (δ ≤ 4.5 ppm) [65]. Consistently, the Cp signals of the remaining complexes **3–7** (apart from **5c**, vide infra) fall within the range 4.97–5.67 ppm, thus indicating cis configuration (acetone-d_6_ or CDCl_3_ solutions). However, two sets of signals are generally observed in the spectra of **3–7** with a ratio variable between 1.2 and 2, related to E-Z isomerism generated by the two possible orientations of the iminium substituents (R ≠ Y). The E isomer is prevalent in **3a**, **4b**, **6a** and **6b**, exhibiting high-field signals for the N-methyl [e.g., at 3.27 (^1^H) and 41.8 (^13^C) ppm in the case of **3a**]; the opposite Z isomer prevails in **3b**, **3c**, **4c**, **5b**, **5c**, **6c** and **7** [e.g., for **3b**: δ(NMe) = 3.89 (^1^H) and 47.7 (^13^C) ppm]. Analogous features were generally recognized on related vinyliminium complexes with R = CH_2_Ph or Xyl and Y = Me (see Introduction and Figure 1) [39,66]. In the case of **5c**, a comparison of the NMR data with the library of data available in the literature points out the existence in CDCl_3_ solution of trans-Z and cis-E isomers, in ca. 2:1 ratio; in detail, the Cp and NMe groups resonate at 4.85, 4.35 and 3.69 ppm in the former isomer, and at 5.59, 5.40 and 2.90 in the latter (^1^H spectra).

Salient ^13^C NMR features of **2–7** concern the carbon nuclei constituting the C_3_ bridging chain, which have been recognized at 223.6–230.4 ppm (C^1^), 52.0–67.1 ppm (C^2^) and 201.9–210.9 ppm (C^3^). The typical low-field values for C^1^ and C^3^ reflect the (amino)alkylidene and bridging alkylidene nature of these centers, respectively [31]; on the other hand, the interval found for C^2^ indicates a partial alkenic nature, and the presence of an alkyl substituent causes a downfield shift by 10–15 ppm (e.g., δ = 52.9 ppm for **2a** and 65.7 ppm for **2c**).

**Figure 2:**
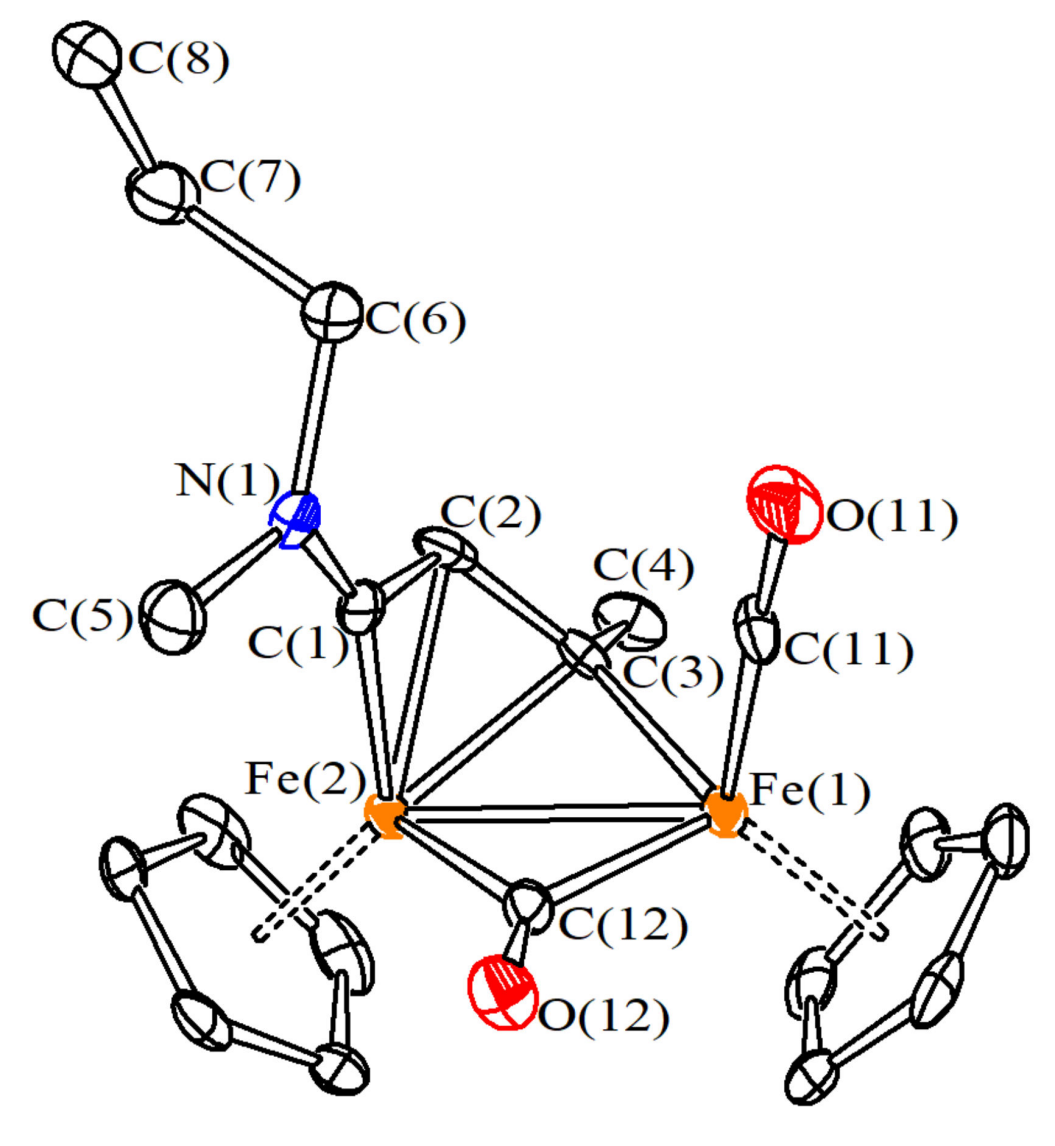
View of the cation of **3a**. H atoms have been omitted for clarity. Displacement ellipsoids are at the 50% probability level. Selected bond lengths (Å) and angles (°): Fe(1)-Fe(2) 2.5426(7), Fe(1)-C(11) 1.763(3), Fe(1)-C(12) 1.911(4), Fe(2)-C(12) 1.934(4), Fe(2)-C(1) 1.851(3), Fe(2)-C(2) 2.055(3), Fe(2)-C(3) 2.036(4), Fe(1)-C(3) 1.940(4), C(1)-N(1) 1.285(4), C(1)-C(2) 1.419(5), C(2)-C(3) 1.409(5), N(1)-C(5) 1.466(5), N(1)-C(6) 1.481(4), C(6)-C(7) 1.487(5), C(7)-C(8) 1.319(5), C(11)-O(11) 1.144(4), C(12)-O(12) 1.172(4), Fe(1)-C(11)-O(11) 178.2(4), Fe(1)-C(12)-Fe(2) 82.79(15), Fe(1)-C(3)-Fe(2) 79.47(14), Fe(1)-C(3)-C(2) 120.7(3), C(3)-C(2)-C(1) 116.2(3), C(2)-C(1)-Fe(2) 76.6(2), C(1)-N(1)-C(5) 121.7(3), C(1)-N(1)-C(6) 121.3(3), C(5)-N(1)-C(6) 117.0(3), N(1)-C(6)-C(7) 112.7(3), C(6)-C(7)-C(8) 122.5(4).

The structures of **2c** and **3a** were confirmed by single-crystal X-ray diffraction studies: a view of the cation of **3a** is shown in Figure 2, with relevant bonding parameters in the caption, while a view of the cation of **2c** is supplied as Supplementary Materials (Figure S33). In both structures, the Cp ligands are cis oriented, while the N-substituents in **3a** are arranged according to the E configuration. In **3a**, the bridging C^3^ carbon is slightly asymmetric between the two irons [Fe(2)-C(3) 2.036(4) and Fe(1)-C(3) 1.940(4) Å], while the bridging carbonyl exhibits a lower asymmetry [Fe(2)-C(12) 1.934(4) and Fe(1)-C(12) 1.911(4) Å]. The N(1)-C(1) [1.285(4) Å], C(1)-C(2) [1.419(5) Å] and C(2)-C(3) [1.409(5) Å] distances evidence an extensive charge delocalization, as already found in similar complexes [39,44,66], suggesting that the representation of the bridging ligand as vinyliminium is appropriate but not univocal. In particular, a comparison between the C(2)-C(3) distance and C(6)-C(7) [1.487(5) Å] and C(7)-C(8) [1.319(5) Å] outlines that the former is intermediate between a single and a double C*sp^2^*-C*sp^2^* bond.

### 3.2. Solubility and Stability in Water and Determination of Octanol–Water Partition Coefficients

The water solubility of complexes was measured by means of ^1^H NMR spectroscopy (Table 3). In D_2_O, most of the complexes showed an appreciable solubility, falling in the millimolar range, and an isomer ratio slightly different with respect to what is found in acetone-d_6_ or CDCl_3_. The solubilities of **3a–c**, **4a–c** and **6a** (ranging from 1.02 to 4.02 g∙L^−1^) are comparable to that of cisplatin (ca. 3 g∙L^−1^) and other common platinum drugs [67,68,69,70]. On the other hand, **2b,c**, containing more than one phenyl group, and **7**, containing a naphthyl, are almost insoluble in D_2_O.

Next, the stability of the complexes was assessed by ^1^H NMR spectroscopy in DMSO-d_6_/D_2_O solutions, stored at 37 °C for 72 h (Table 3). In general, the complexes exhibited a considerable stability, with an average of 83% of starting material detected at the end of the experiment. Especially those complexes with R′ = Me and R″ = H appear robust (mean percentage = 90%). The slow decomposition of the complexes is featured by the formation of a minor amount of precipitate suggesting extensive rupture of the diiron scaffold [71], while new organometallic species were not detected in solution. The octanol–water partition coefficients were measured by means of a UV–Vis technique (Table 3, see Experimental for details). Most complexes display a substantial *amphiphilic* character, with Log *P*_ow_ values ranging from −0.77 to 1.30. In general, the presence of the phenyl group on the C^3^ carbon leads to a significant increase in lipophilicity, while the allyl group on the iminium moiety determines the opposite effect. Complex **2b**, containing three phenyl units, displays the highest Log *P*_ow_ value (1.4) and was excluded from the biological tests due to insufficient solubility.

### 3.3. Cytotoxicity

Firstly, the antiproliferative activity of the diiron complexes was assessed on human ovarian carcinoma A2780 cancer cells and, to estimate the selectivity, also on nontumoral Balb/3T3 clone A31 cells (Table 4 and Figure S34). Cisplatin was employed as a reference. In general, diiron complexes exhibit strong cytotoxicity against the considered cancer cell line, with IC_50_ values falling in the low micromolar range. Furthermore, an impressive selectivity was recognized with respect to the Balb/3T3 cell line: the selectivity index (S.I.) ranges from 7 to 52, while the value for cisplatin is only 2 (Table 4).

The cytotoxicity of the complexes against the A2780 cancer cell line approximately correlates with the lipophilicity, and actually the most hydrophilic compounds **3a** and **3c** (Log *P*_ow_ < −0.7), bearing R = CH_2_CH = CH_2_, display a relatively lower potency (IC_50_ ≈ 17 μM). On the other hand, **4a**, **4c**, **5a**, **5b**, **6a** and **6c**, bearing R = cyclohexyl, benzyl or 4-methoxyphenyl, show a potent activity despite the negative Log *P*_ow_ values.

The analogous complexes **5b** and **5c**, differing to each other for the alkyl chain on C^2^ and C^3^, display comparable activity. The activity of **3a** (IC_50_ = 17.7 ± 0.8 μM, Log *P*_ow_ = −0.75) is enhanced compared to that previously determined on the same cell line for the analogous complex [Fe_2_Cp_2_(CO)(μ-CO){μ-η^1^:η^3^-C^3^(Me)C^2^HC^1^NMe_2_}]CF_3_SO_3_ (IC_50_ = 35 ± 3 μM, Log *P*_ow_ = −0.3) [39], indicating that the replacement of one methyl *N*-substituent with the allyl may improve the anticancer activity. Conversely, IC_50_ values of 0.50 ± 0.06 μM and 0.90 ± 0.06 μM were previously found for, respectively, [Fe_2_Cp_2_(CO)(μ-CO){μ-η^1^:η^3^-C^3^(Me)C^2^(Me)C^1^NMe(Xyl)}]CF_3_SO_3_ (Log *P*_ow_ = 0.0) and [Fe_2_Cp_2_(CO)(μ-CO){μ-η^1^:η^3^-C^3^(Ph)C^2^HC^1^NMe(Xyl)}]CF_3_SO_3_ (Log *P*_ow_ = 0.4) [39], thus outlining the beneficial effect provided by a xylyl ring among possible aryl groups (see data related to **7** and **6b**).

Note that here R = cyclohexyl, benzyl and 4-methoxyphenyl supply the highest selectivity indexes, and especially the combination with R′ = R″ = Me or Et is convenient (average S.I. ≥ 30). It is worth mentioning that R = cyclohexyl and R = 4-methoxyphenyl contribute to the best cytotoxicity profiles also concerning the series of aminocarbyne complexes **1a–f** [37]. Complex **4c**, derived from the assembly of cyclohexylisocyanide and 2-butyne (Table 2), appears as the most performant within the series of vinyliminium complexes **2–7**. The favorable effect on the anticancer activity provided by the decoration of metal complexes with the cyclohexyl (Cy) moiety has been documented in the literature, attributed to the compact and hydrophobic structure of Cy facilitating the entrance of the drug into the tumor cell [72,73,74].

A selection of the most promising compounds was further investigated. First, the cytotoxicity of **3b**, **4a**, **4c**, **5b** and **6c** was measured on the cisplatin-resistant A2780cisR cell line. Overall, a loss of activity was observed with respect to the cisplatin-sensitive cell line (A2780), nevertheless **4a**, **4c** and **5b** maintain a higher level of activity than that of cisplatin under the same conditions.

### 3.4. ROS Production

**Figure 3:**
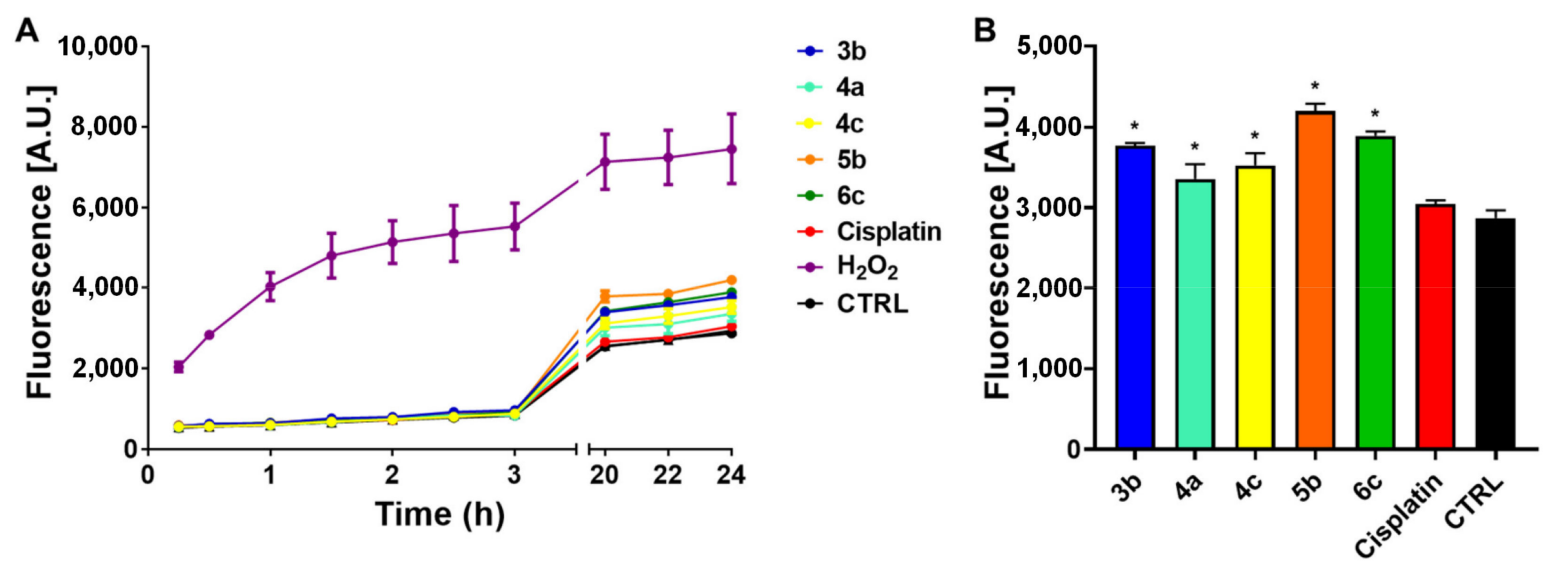
Fluorescence kinetic measurements of intracellular reactive oxygen species (ROS). A2780 cells incubated (**A**) up to 24 h and (**B**) for 24 h with 20 μM of diiron complexes at 37 °C and 5% CO_2_. Results are expressed as the mean of three analyses with standard deviation. Values marked with * are significantly different from the control (* *p* < 0.05).

Previous findings pointed out that diiron vinyliminium complexes exert their anticancer activity mainly by unbalancing cell redox homeostasis, and this phenomenon may be ascribable to several routes possibly taking place inside the cells: (1) complex reduction by neutralization of the net cationic charge; (2) fragmentation into monoiron derivatives; and (3) disassembly of the diiron core releasing the two Fe^+I^ centers [36,39,40,45]. According to this premise, we considered of interest to evaluate the ability of selected complexes, **3b**, **4a**, **4c**, **5b** and **6c**, to induce intracellular ROS production. Thus, fluorescence measurements were carried out with the DCFH-DA assay, by exposing A2780 cells to, respectively, the diiron complexes, the reference drug cisplatin and H_2_O_2_ (positive control). All the diiron complexes elicited an appreciable ROS production, significantly higher than that of cisplatin, especially after ca. 20 h and progressively increasing up to 24 h (Figure 3). The benzyl complex **5b** revealed the most effective of the series in inducing the ROS production, and this outcome may be associated with the high cytotoxicity exhibited by this complex against the cancer cells, despite its relatively low hydrophobicity.

### 3.5. Protein Binding Studies

Recent results on the anticancer activity of the aminocarbyne complex **1e** highlighted a possible mechanism involving the inhibition of the selenoenzyme thioredoxin reductase (TrxR) [36]; more specifically, substantial inhibition of this enzyme was detected in human pancreatic PSN-1 cancer cells. Former studies by other authors demonstrated a similar mode of action by ferrocenyl drug candidates [75]. Therefore, we became interested in elucidating the potentiality of the new vinyliminium complexes to inhibit TrxR.

During the past years, high-resolution electrospray ionization mass spectrometry (HR-ESI-MS) has emerged as a valuable and powerful tool to study the formation of adducts derived from the interaction of metallodrugs with biomolecules [76,77,78]. Moreover, this technique allows the identification of the precise nature of the fragments attached to protein side chains and, thus, to infer some relevant mechanistic information [79,80,81].

Since the amount of native TrxR required for the ESI-MS experiment, albeit low, can hardly be found commercially available at a reasonable cost, for the present study we opted for a model system that has been already successfully employed [82]. The model is a synthetic dodecapeptide (**TrxR-pept**) mimicking the C-terminal tryptic portion of the TrxR enzyme, consisting in the amino acidic sequence {Ac-SGGDILQSG[CU]G-NH_2_} and featured by the presence of the peculiar {-Cys-Sec-} motif (Cys = cysteine; Sec = selenocysteine).

**Figure 4:**
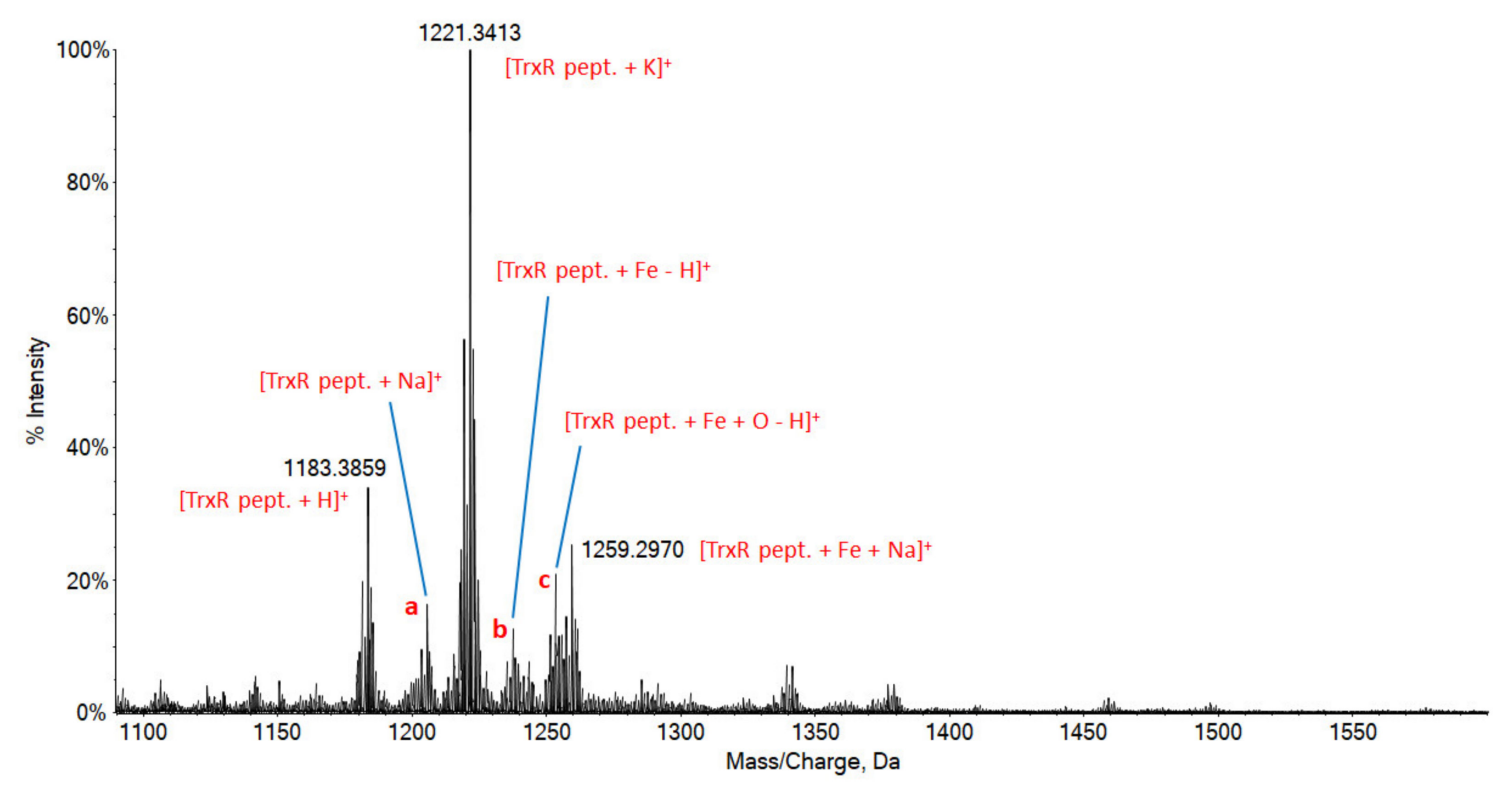
High-resolution ESI mass spectrum of **1e** incubated for 24h at 37 °C with 10^−5^ M **TrxR-pept** solution in water; 1:1 peptide to complex ratio. 0.1% *v*/*v* of formic acid was added just before infusion. Unlabeled peaks: **a**, *m*/*z* 1205.3771; **b**, *m*/*z* 1237.3218; **c**, *m*/*z* 1253.3236.

First, we verified the suitability of the model system by analyzing its interaction with complex **1e**, for which TrxR inhibition was previously recognized by enzymatic assay (vide infra). The resulting ESI mass spectrum is shown in Figure 4. It contains three main peaks at *m/z* 1183.3859, due to unreacted **TrxR-pept**, and at *m/z* 1205.3771 and 1221.3413, corresponding to the adducts of **TrxR-pept** with sodium and potassium, respectively. In addition, three more peaks of lower intensity are ascribable to the metalated dodecapeptide. Notably, the signal at *m/z* 1237.3218 is in good accordance with the presence of a [**TrxR-pept** + Fe − H]^+^ species. Moreover, the signals at *m/z* 1253.3236 and 1259.2970 were assigned to the iron-bound **TrxR-pept** with the additional presence of one oxygen atom and one sodium ion, respectively. Intriguingly, the degradation of diiron bis-cyclopentadienyl carbonyl complexes inside the tumor cell, releasing the iron atoms, has been hypothesized to represent a major mechanism for the antiproliferative activity [36,37,39,45].

At this stage of the study, we were not able to ascertain the oxidation state of the iron center nor its coordinative surrounding; however, it is reasonable to assume that the binding of iron is made possible by the coordination of sulfur and selenium atoms belonging to the {-Cys-Sec-} unit. This hypothesis is in alignment with the significant TrxR inhibitory activity manifested by **1e** [37], which suggests a direct involvement of the TrxR catalytic site.

Following the coherent results obtained for **1e**, we extended the same method to test the TrxR inhibition ability of selected vinyliminium complexes. Thus, **2c**, **3b**, **4a**, **4c**, **5b**, **6b**, **6c** and **7** were incubated at 37 °C for 24 h with **TrxR-pept** in 1:1 complex-to-peptide ratio.

The recorded ESI mass spectra share a common pattern resembling that obtained for **1e** (see Figure S35 for a representative spectrum). More in detail, in addition to the signals of unreacted **TrxR-pept** and its adducts with sodium and potassium, one relevant signal at *m/z* 1237.3172 was recognized, attributed to [**TrxR-pept** + Fe − H]^+^. This outcome suggests that diiron vinyliminium complexes may actually act as inhibitors of the thioredoxin reductase enzyme, analogously to diiron aminocarbyne complexes such as **1e**.

Then, we analyzed the interaction of the vinyliminium complexes **2c**, **3b**, **4a**, **4c**, **5b**, **6b**, **6c** and **7** with a small model protein, i.e., cytochrome c (Cyt c). The resulting ESI mass spectra indicated the substantial chemical inertness of the cationic part of the complexes towards Cyt c. In fact, the spectra only show the signal due to the unreacted protein (12358.451 Da) and two other peaks at 12508.414 and 12659.366 Da, corresponding to the adducts of Cyt c with one and two CF_3_SO_3_^−^ anions, respectively (Figure S36). This absence of reactivity confirms the strong robustness of **2–7**, and represents a partial indication that the anticancer activity of these complexes is selectively directed to the TrxR inhibition, the possible side-reactions with other cellular or plasma proteins being unlikely. Accordingly, previous fluorescence experiments on the interaction of analogous diiron complexes with bovine serum albumin (BSA) pointed out the occurrence of a reversible binding event [39,46], potentially adequate to BSA-mediated transport and diffusion of the drug [83,84]. Note that the establishment of unselective, strong covalent interactions between a metal drug and proteins is, in principle, one of the routes for off-target reactions causing undesired side effects.

## 4. Conclusions

Diiron complexes based on the {Fe_2_Cp_2_(CO)_2_} scaffold and containing a bridging vinyliminium ligand display some notable properties for a potential drug, i.e., the presence of a biocompatible metal element, straightforward synthesis from inexpensive precursors, appreciable water solubility and/or amphiphilicity, and remarkable stability in aqueous media. The wide structural variability is guaranteed by the cooperativity provided by the bimetallic system. Following preliminary studies on other similar compounds, here we have synthesized a series of new diiron vinyliminium complexes, which have been investigated for their in vitro anticancer activity. In particular, a variety of alkyl and aryl groups (R, Y) on the iminium function has been screened for the first time. Overall, the results point out a significant and fine influence of all vinyliminium substituents (R, Y, R′ and R″) on the physico-chemical properties of the complexes and their cytotoxicity, and especially the choice of R and Y is crucial to provide an optimal profile, with both electronic and steric factors presumably playing a role. Complex **4c**, bearing a cyclohexyl moiety on the nitrogen, emerges for its high antiproliferative activity (associated with a slightly negative Log *P_ow_* value) towards A2780 and A2780cisR cancer cells and impressive selectivity compared to Balb/3T3 healthy cells (selectivity indexes are 52 and 8, respectively). In accordance with previous findings, targeted studies reveal the probable interference of the complexes with the cellular redox balance, triggering the production of reactive oxygen species. Moreover, mass spectrometry experiments suggest that, although the complexes seem sufficiently robust to resist degradation by common proteins, the peculiar structure of the catalytic site of TrxR might accelerate the intracellular disassembly of the diiron scaffold, resulting in the enzyme inhibition via incorporation of iron. Advanced studies are in course to develop diiron vinyliminium complexes as a suitable class of organometallic anticancer candidates.

## Figures and Tables

**Table 1:** Crystal data and measurement details for **2c** and **3a**.

	2c	3a
Formula	C_32_H_30_F_3_Fe_2_NO_5_S	C_21_H_22_F_3_Fe_2_NO_5_S
FW	709.33	569.15
T, K	100(2)	100(2)
*λ*, Å	0.71073	0.71073
Crystal system	Monoclinic	Monoclinic
Space group	*P2*_1_/*c*	*P2*_1_/*c*
*a*, Å	17.2348(14)	9.9306(6)
*b,* Å	8.9679(7)	12.5791(7)
*c*, Å	19.8255(14)	17.5345(10)
*β*,°	104.380(3)	90.604(2)
Cell volume, Å^3^	2968.2(4)	2190.3(2)
Z	4	4
*D_c_*, g∙cm^−3^	1.587	1.726
μ, mm^−1^	1.110	1.480
F(000)	1456	1160
Crystal size, mm	0.18 × 0.15 × 0.11	0.21 × 0.18 × 0.13
θ limits,°	2.121–25.081	1.993–25.994
Reflections collected	28,217	39,294
Independent reflections	5184 [*R_int_* = 0.0817]	4267 [*R_int_* = 0.1414]
Data/restraints/parameters	5184/1041/513	4267/0/300
Goodness on fit on F^2^	1.283	1.142
*R*_1_ (*I* > 2σ(*I*))	0.1207	0.0480
*wR*_2_ (all data)	0.2512	0.0964
Largest diff. peak and hole, e Å^−3^	0.679/−1.156	0.607/−0.504

**Table 2:** Diiron vinyliminium complexes (**2–7**) obtained by aminocarbyne precursors (**1a–f**) according to Scheme 17.

Precursor	R	Y	R′	R″	Product
**1a**	CH_2_Ph	CH_2_Ph	Me	H	**2a**
**1a**	CH_2_Ph	CH_2_Ph	Ph	H	**2b**
**1a**	CH_2_Ph	CH_2_Ph	Me	Me	**2c**
**1b**	CH_2_CH = CH_2_	Me	Me	H	**3a**
**1b**	CH_2_CH = CH_2_	Me	Ph	H	**3b**
**1b**	CH_2_CH = CH_2_	Me	Me	Me	**3c**
**1c**	C_6_H_11_ = Cy	Me	Me	H	**4a**
**1c**	Cy	Me	Ph	H	**4b**
**1c**	Cy	Me	Me	Me	**4c**
**1d**	CH_2_Ph	Me	Me	H	**5a**
**1d**	CH_2_Ph	Me	Me	Me	**5b**
**1d**	CH_2_Ph	Me	Et	Et	**5c**
**1e**	4-C_6_H_4_OMe	Me	Me	H	**6a**
**1e**	4-C_6_H_4_OMe	Me	Ph	H	**6b**
**1e**	4-C_6_H_4_OMe	Me	Me	Me	**6c**
**1f**	2-naphthyl	Me	Me	Me	**7**

**Table 3:** Solubility of diiron complexes in D_2_O at 21 °C (^1^H NMR, Me_2_SO_2_ internal standard), residual % in DMSO-d_6_/D_2_O (2:1 *v*/*v*) after 72 h at 37 °C (^1^H NMR, Me_2_SO_2_ internal standard) and octanol–water partition coefficients at 21 °C (Log *P*_ow_, UV–Vis). ^a^ Stability evaluated in neat D_2_O.

Compound	Solubility/mol·L^−1^	Solubility/g·L^−1^	Stability %	Log *P*_ow_
**2a**	4.33 × 10^−4^	0.30	81	0.45 ± 0.04
**2b**	<1 × 10^−4^	<0.08	=	1.4 ± 0.2
**2c**	<1 × 10^−4^	<0.07	87	0.56 ± 0.08
**3a**	6.43 × 10^−3^	3.66	93 ^a^	−0.75 ± 0.01
**3b**	2.34 × 10^−3^	1.48	68	−0.09 ± 0.01
**3c**	6.89 × 10^−3^	4.02	85 ^a^	−0.77 ± 0.01
**4a**	3.49 × 10^−3^	2.13	95	−0.29 ± 0.03
**4b**	1.52 × 10^−3^	1.02	65	0.41 ± 0.02
**4c**	2.63 × 10^−3^	1.64	95	−0.17 ± 0.01
**5a**	5.41 × 10^−4^	0.34	85	−0.26 ± 0.01
**5b**	1.52 × 10^−3^	0.96	90	−0.43 ± 0.01
**5c**	1.10 × 10^−3^	0.73	84	0.13 ± 0.01
**6a**	2.22 × 10^−3^	1.41	94	−0.46 ± 0.01
**6b**	4.98 × 10^−4^	0.35	76	0.37 ± 0.04
**6c**	1.06 × 10^−3^	0.69	86	−0.21 ± 0.01
**7**	<1 × 10^−4^	<0.07	66	0.38 ± 0.02

**Table 4:** IC_50_ values (μM) determined for diiron complexes and cisplatin on human ovarian carcinoma (A2780), human ovarian carcinoma cisplatin-resistant (A2780cisR) and murine embryonic fibroblast (Balb/3T3 clone A31) cell lines after 72 h exposure. Values are given as mean ± standard deviation. Selectivity index (S.I.) calculated as the ratio between IC_50_ for Balb/3T3 and IC_50_ for A2780.

Compound	A2780	A2780cisR	Balb/3T3	S.I.
**2a**	2.0 ± 0.7	=	14 ± 4	7
**2c**	1.91 ± 0.15	=	18 ± 3	9
**3a**	17.7 ± 0.8	=	>100	>5.6
**3b**	6.0 ± 0.8	29 ± 3	>100	>16
**3c**	17 ± 3	=	>100	>5.9
**4a**	3.3 ± 0.4	11.6 ± 1.3	>100	>30
**4b**	2.6 ± 0.3	=	35 ± 3	13
**4c**	1.8 ± 0.2	11.4 ± 0.9	94 ± 16	52
**5a**	7.1 ± 0.4	=	>100	>14
**5b**	5.1 ± 0.3	12 ± 4	>100	>19
**5c**	3.75 ± 0.14	=	90 ± 8	24
**6a**	3.5 ± 0.5	=	94 ± 4	27
**6b**	3.8 ± 0.4	=	39 ± 3	10
**6c**	3.8 ± 0.4	17 ± 3	>100	>26
**7**	2.2 ± 0.2	=	48 ± 3	22
cisplatin	0.40 ± 0.07	26 ± 3	0.8 ± 0.2	2

## Data Availability

Data are contained within the article or Supplementary Materials.

## Supplementary Materials

The following are available online at https://www.mdpi.com/article/10.3390/pharmaceutics13081158/s1: Figures S1–S32: NMR spectra of complexes; Figure S33: X-ray structure of **2c**; Figure S34: Dose–response cell viability curves; Figures S35–S36: Representative high-resolution ESI mass spectra for the interaction of **3b** with the TrxR dodecapeptide (**TrxR-pept**) and Cyt c. CCDC reference numbers 2087828 (**2c**) and 2087829 (**3a**) contain the supplementary crystallographic data for the X-ray studies reported in this paper. These data can be obtained free of charge at www.ccdc.cam.ac.uk/conts/retrieving.html (or from the Cambridge Crystallographic Data Centre, 12, Union Road, Cambridge CB2 1EZ, UK; e-mail: deposit@ccdc.cam.ac.uk).
