# Supplementary material for: Anticancer Diiron Vinyliminium Complexes: A Structure–Activity Relationship Study
Source: Pharmaceutics. 2021 Jul 27;13(8):1158. doi: 10.3390/pharmaceutics13081158 (PMC8398472; doi:10.3390/pharmaceutics13081158)
Supplement: Supplementary file 1 [file pharmaceutics-13-01158-s001.zip › pharmaceutics-1295660-supplementary.pdf]

pharmaceutics

MDPI

Supplementary Materials: Anticancer Diiron Vinyliminium Complexes: A Structure–Activity Relationship Study

Simona Braccini, Giorgia Rizzi, Lorenzo Biancalana, Alessandro Pratesi, Stefano Zacchini, Guido Pampaloni, Federica Chiellini and Fabio Marchetti

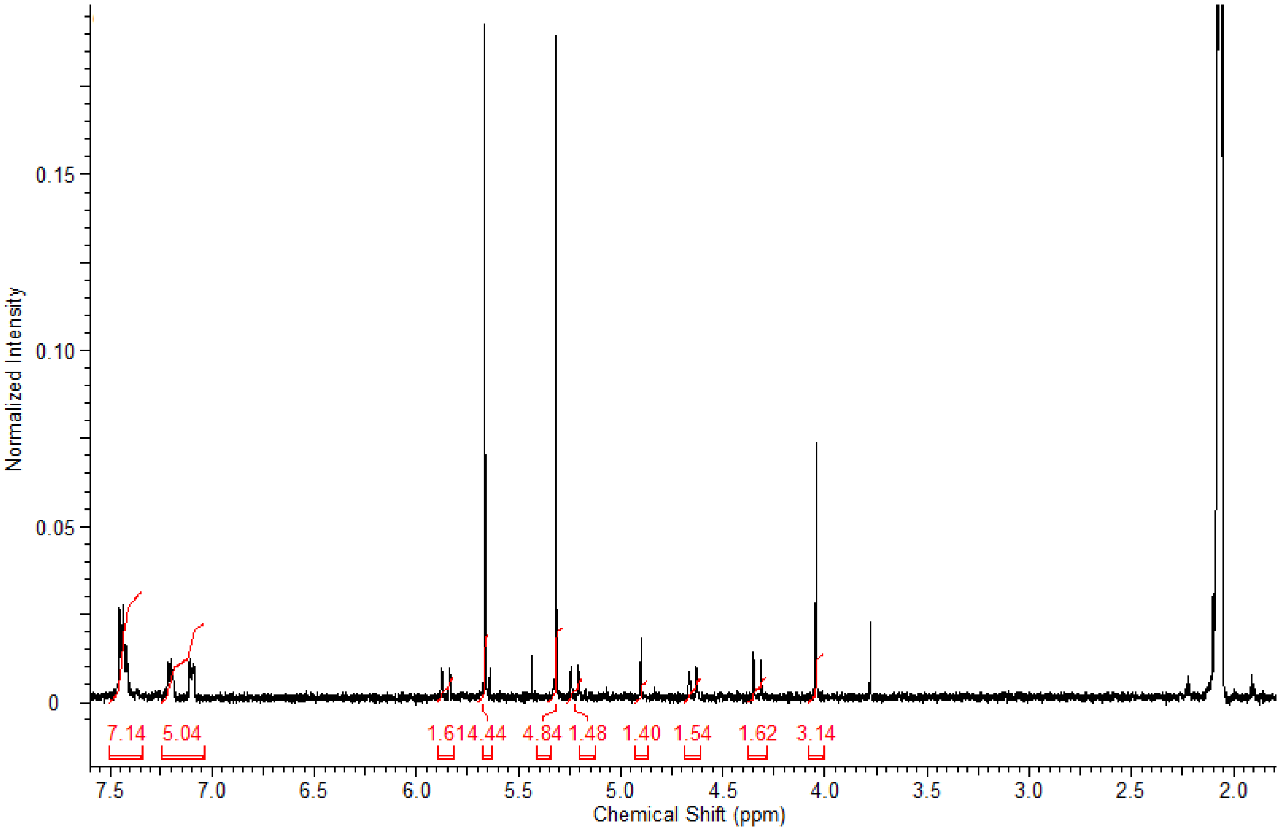

Figure S1. ^{1}H NMR spectrum (401 MHz, acetone-d_6) of **2a**.

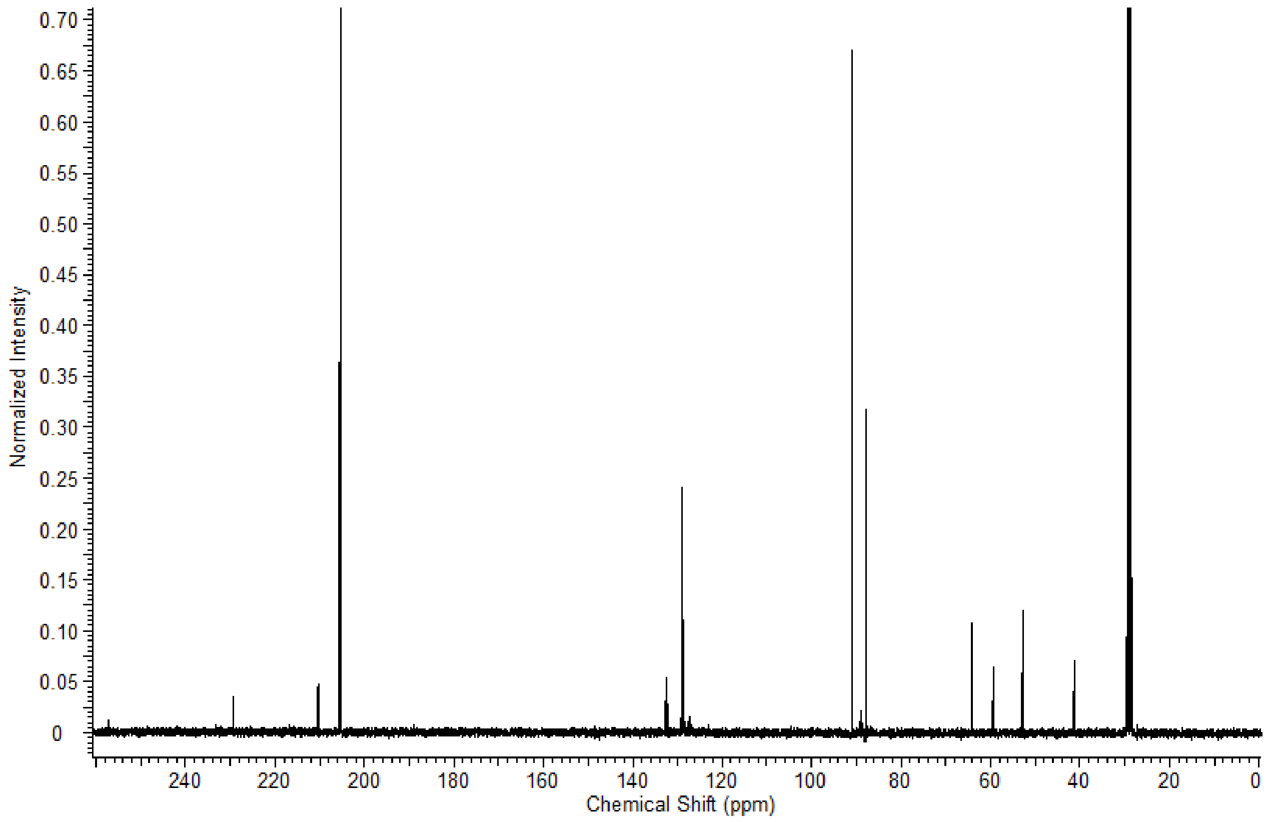

Figure S2. $^{13}C\{^{1}H\}$ NMR spectrum (101 MHz, acetone-d_6) of **2a**.

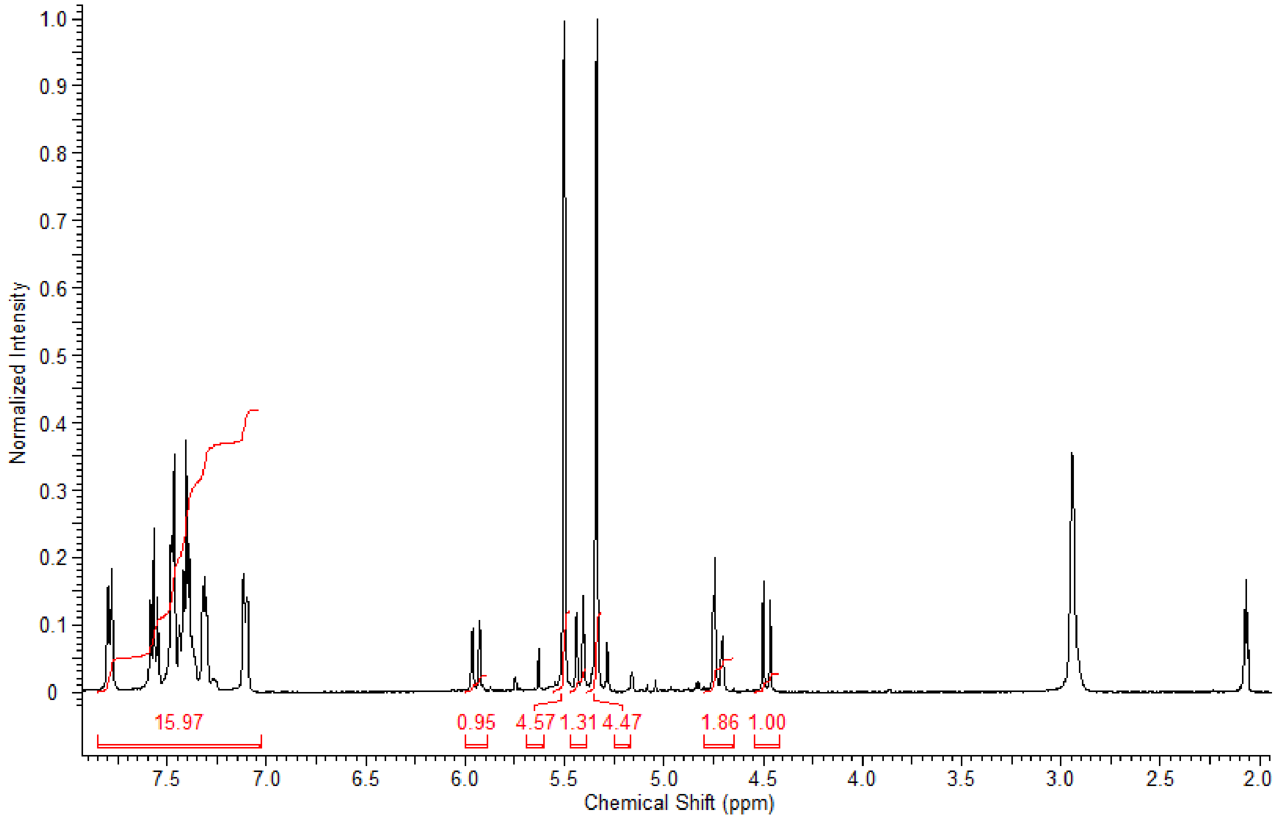

Figure S3. ^{1}H NMR spectrum (401 MHz, acetone-d_6) of **2b**.

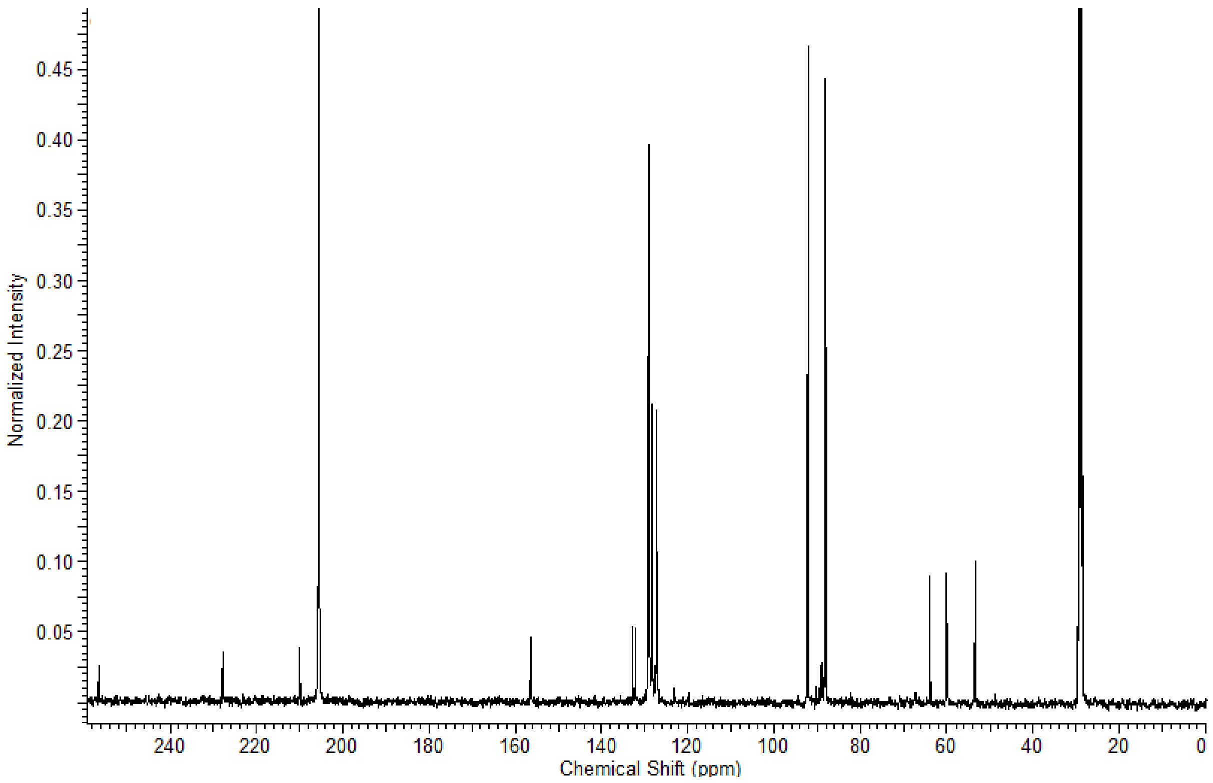

Figure S4. $^{13}C\{^{1}H\}$ NMR spectrum (101 MHz, acetone-d_6) of **2b**.

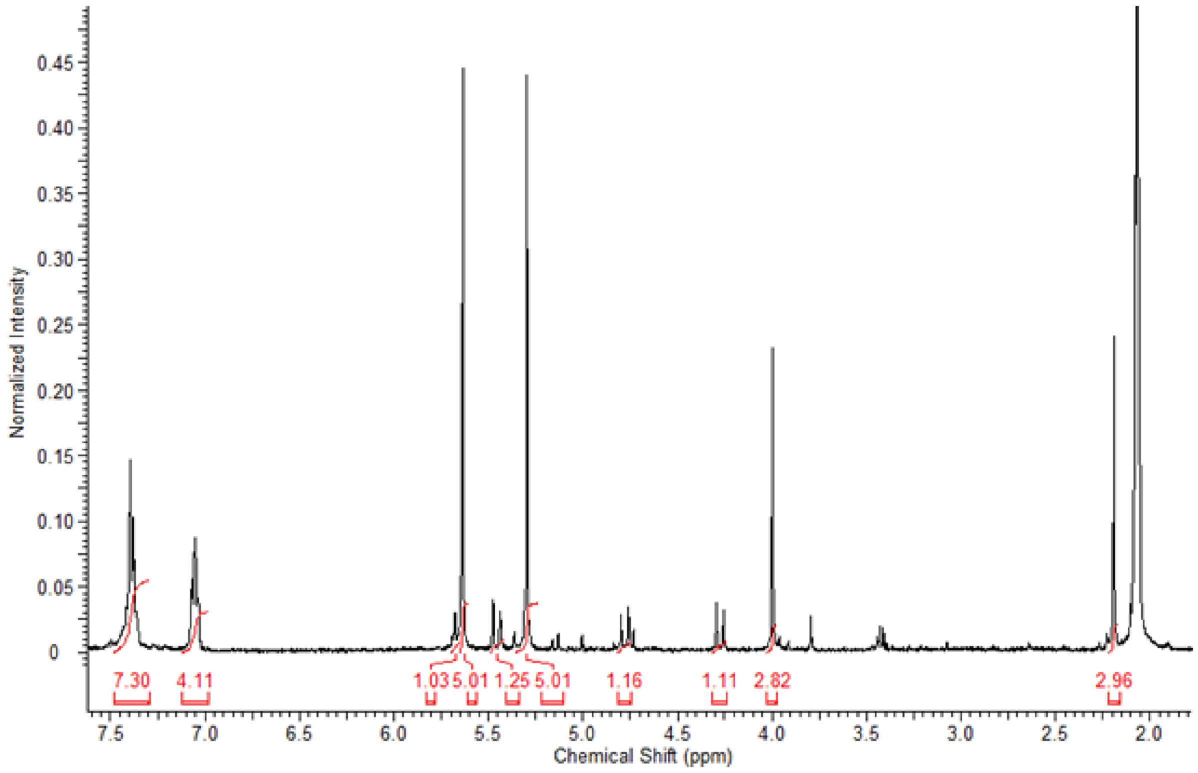

Figure S5. ^{1}H NMR spectrum (401 MHz, acetone-d_6) of **2c**.

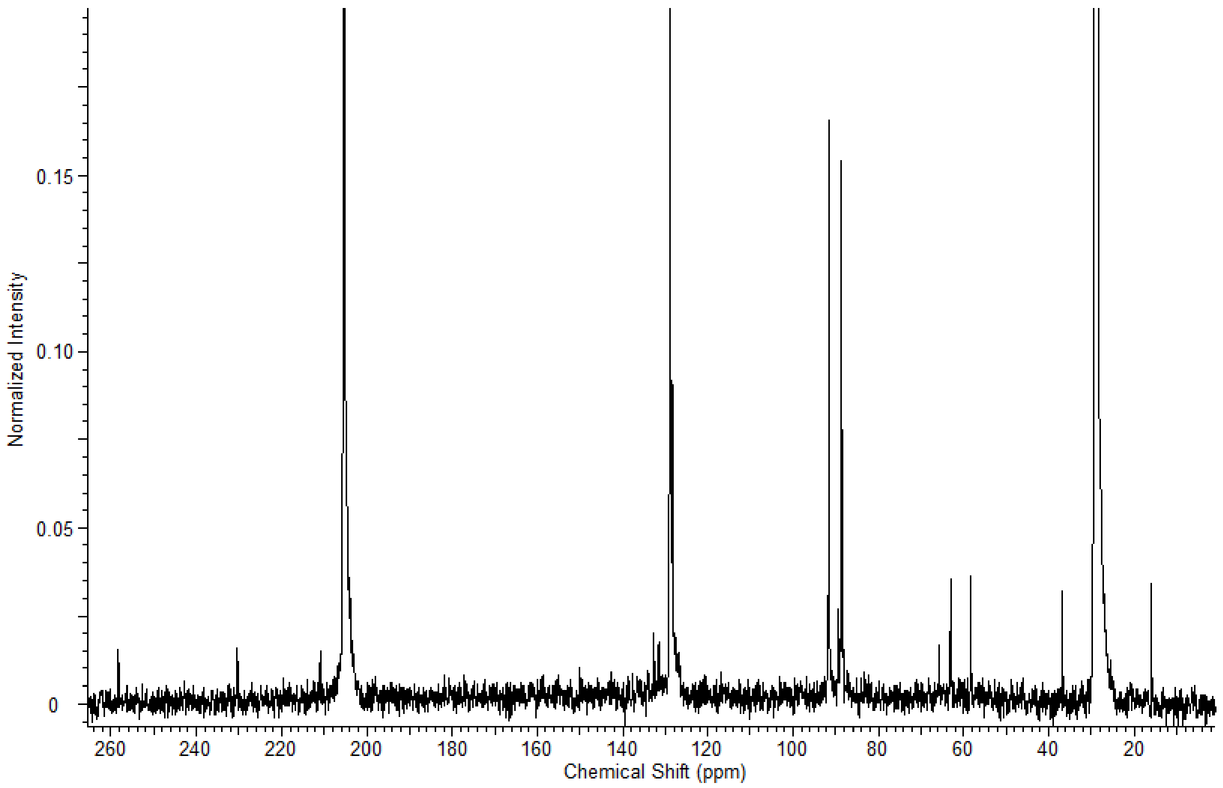

Figure S6. $^{13}C\{^1H\}$ NMR spectrum (101 MHz, acetone-d$_6$) of **2c**.

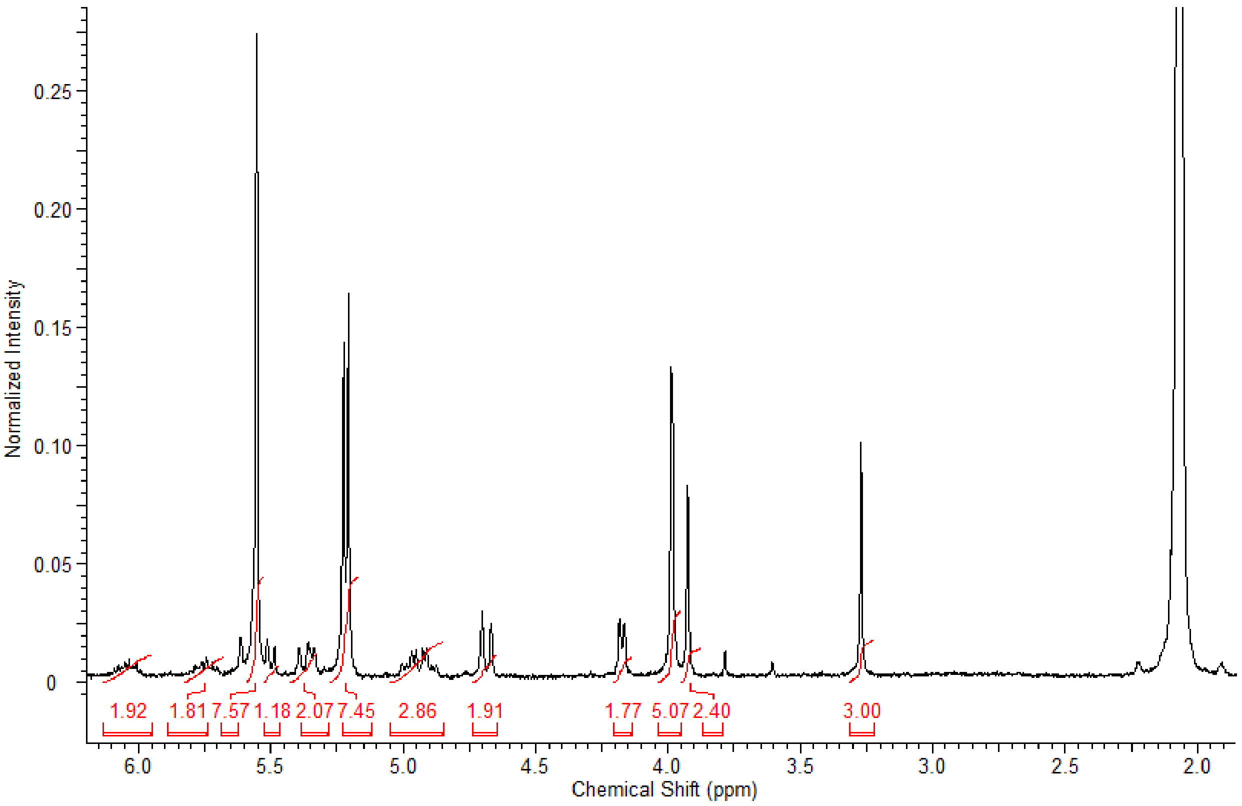

Figure S7. ^1H NMR spectrum (401 MHz, acetone-d$_6$) of **3a**.

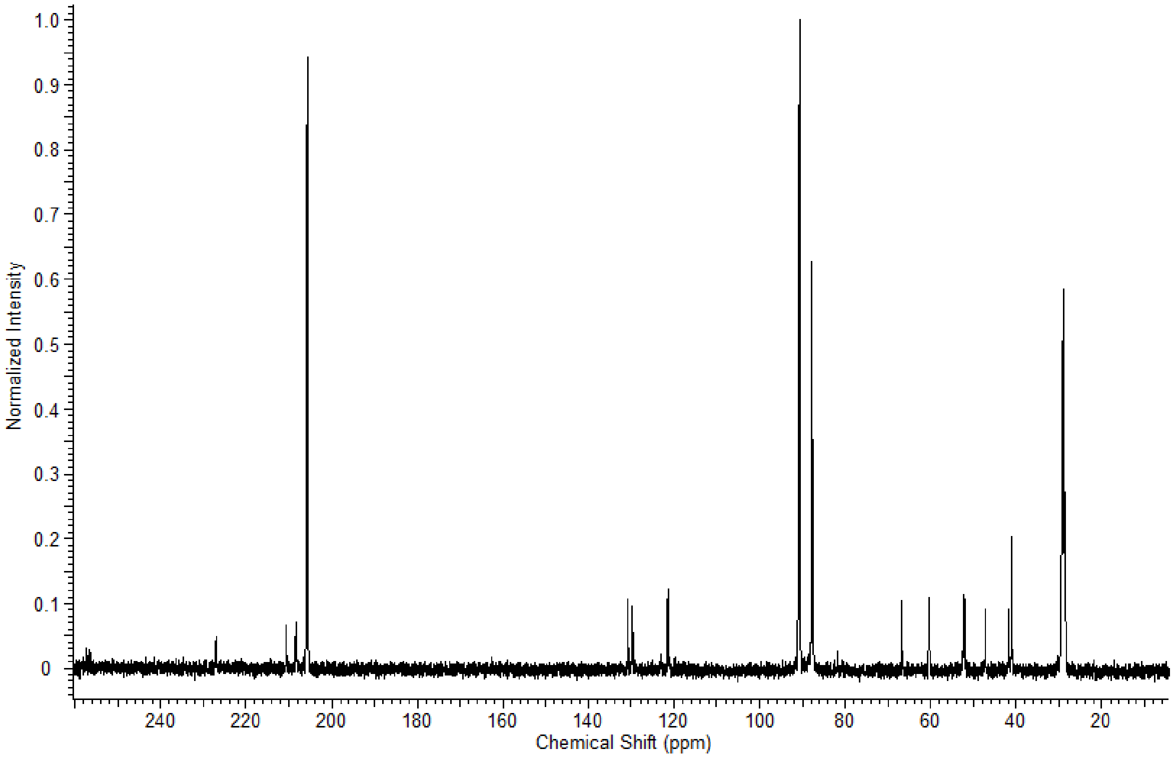

Figure S8. $^{13}C\{^1H\}$ NMR spectrum (101 MHz, acetone-d_6) of **3a**.

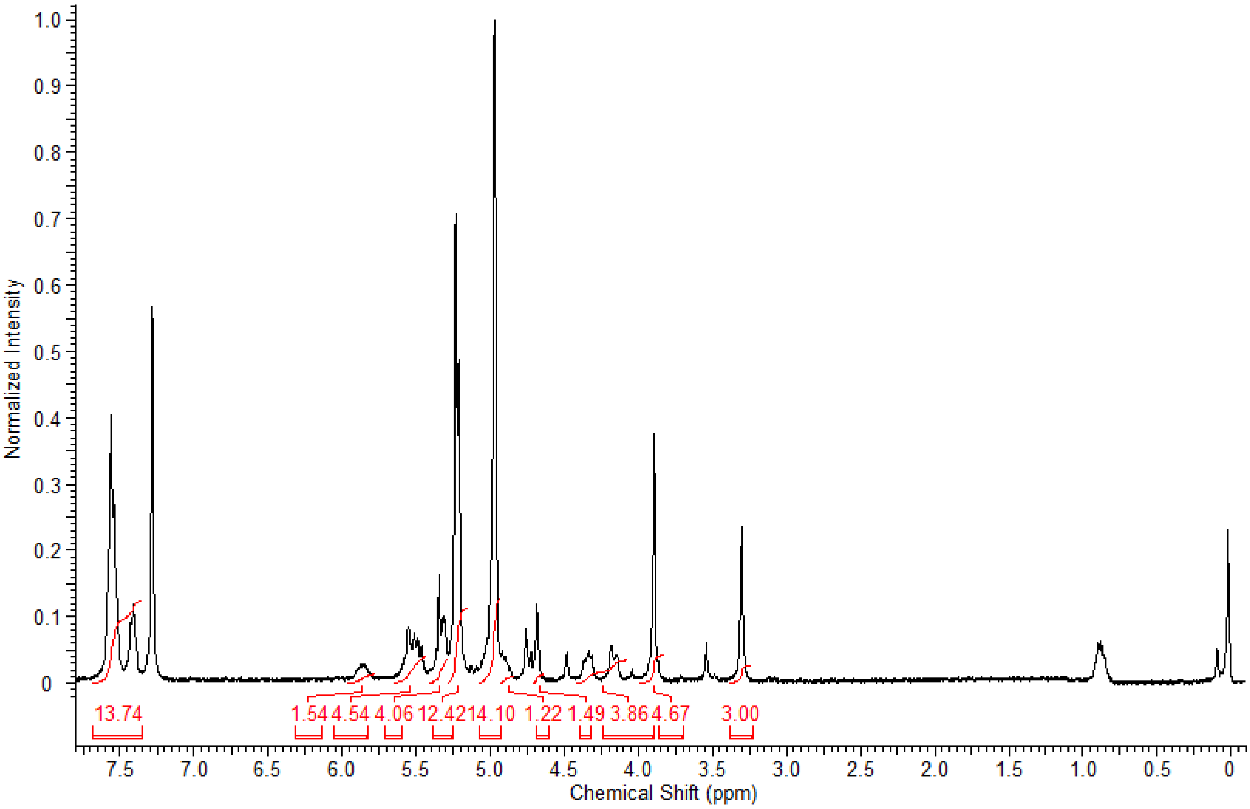

Figure S9. ^1H NMR spectrum (401 MHz, $CDCl_3$) of **3b**.

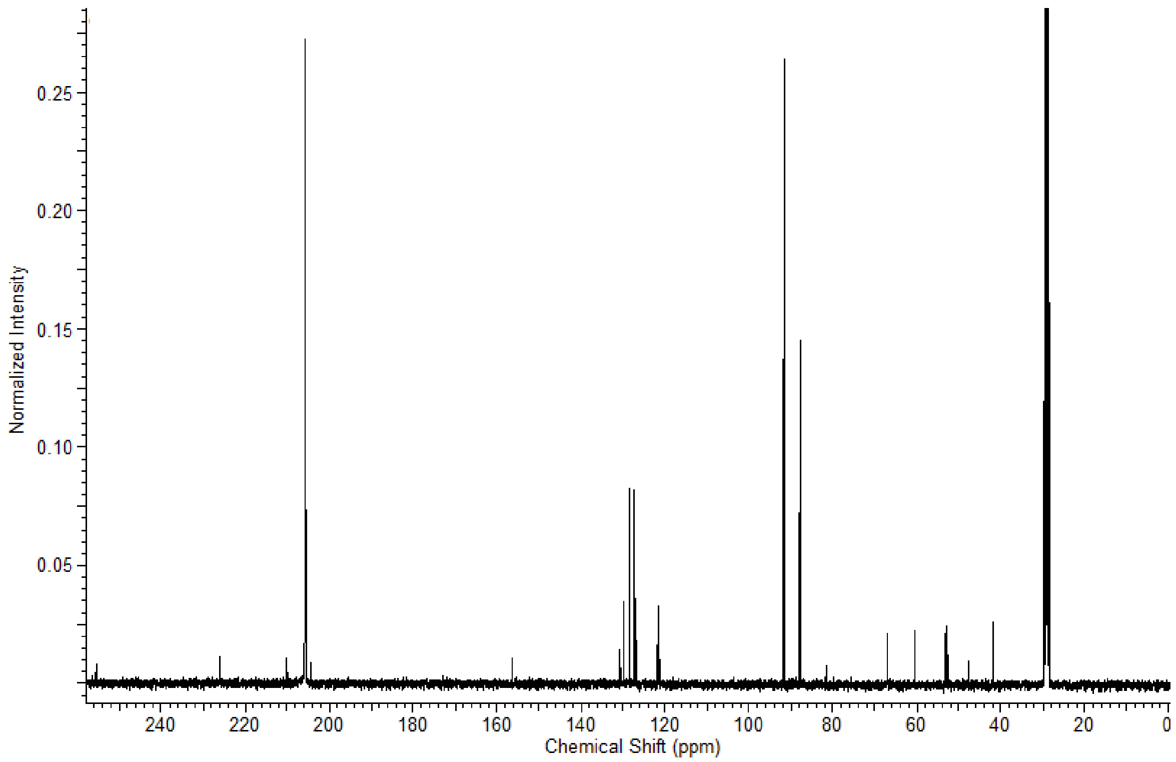

Figure S10. $^{13}C\{^1H\}$ NMR spectrum (101 MHz, acetone-d_6) of **3b**.

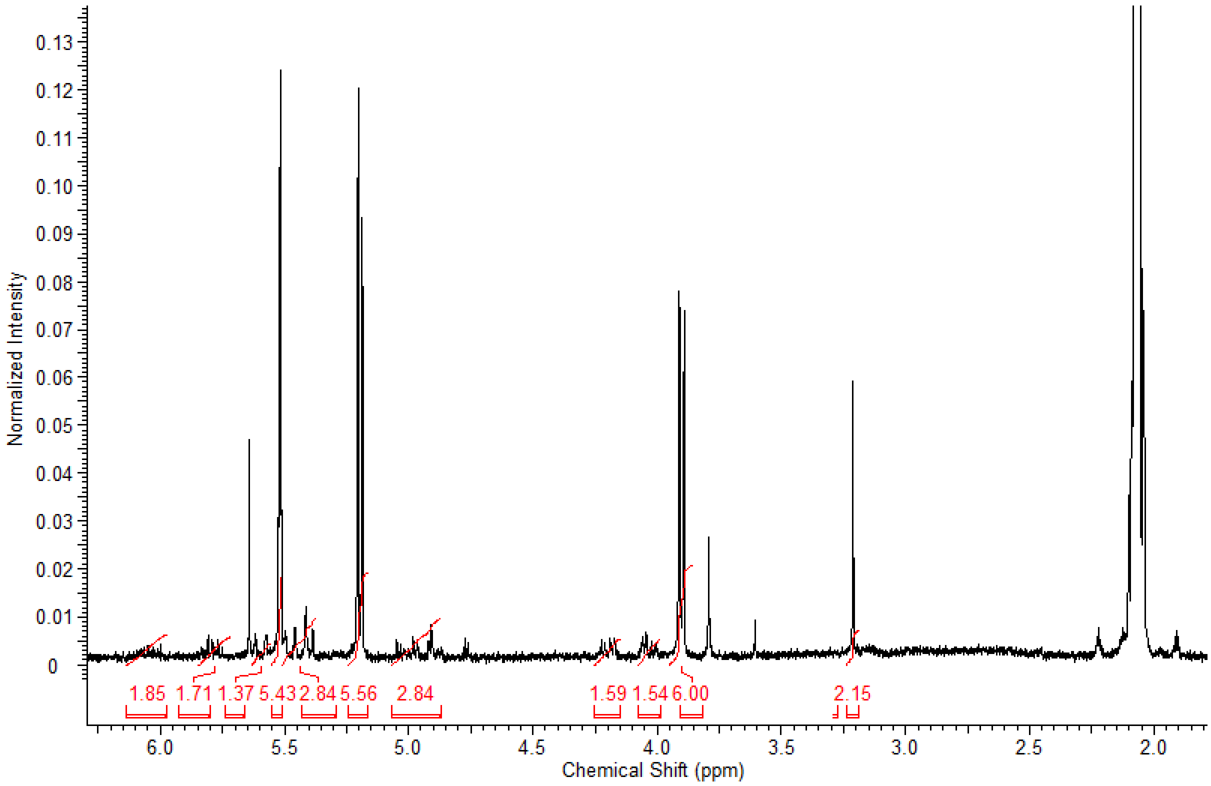

Figure S11. ^1H NMR spectrum (401 MHz, acetone-d_6) of **3c**.

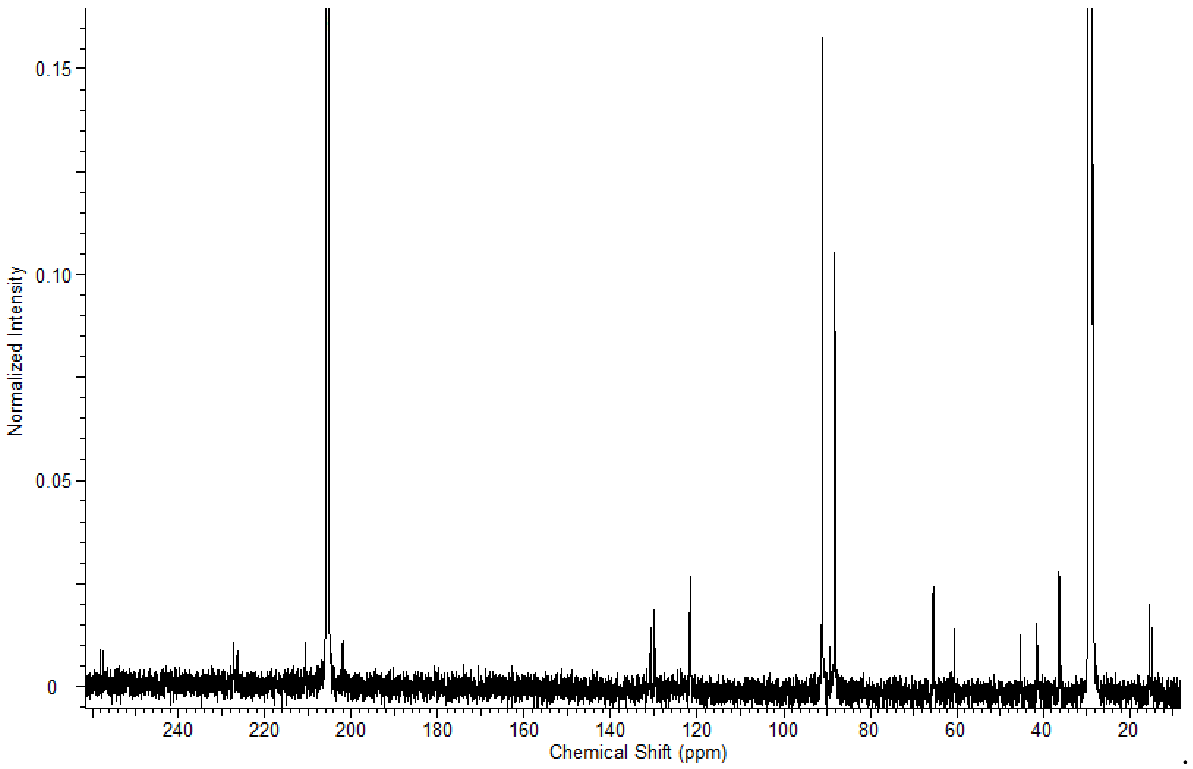

Figure S12. $^{13}C\{^{1}H\}$ NMR spectrum (101 MHz, acetone-d$_6$) of **3c**.

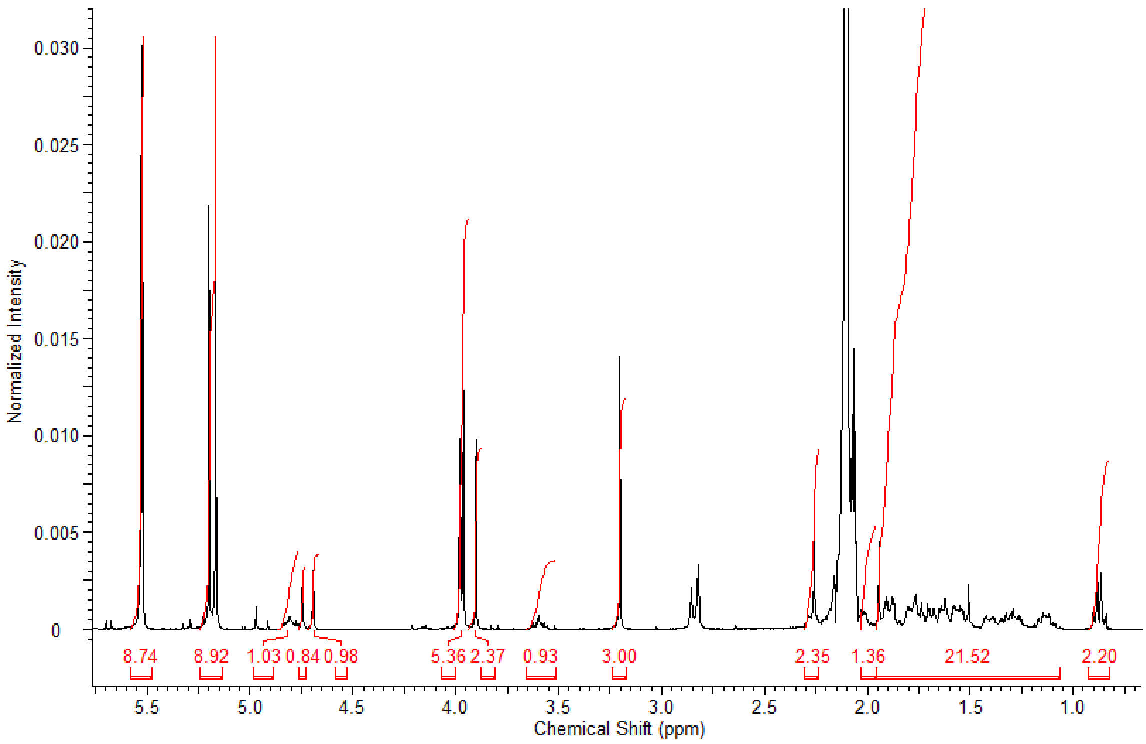

Figure S13. ^{1}H NMR spectrum (401 MHz, acetone-d$_6$) of **4a**.

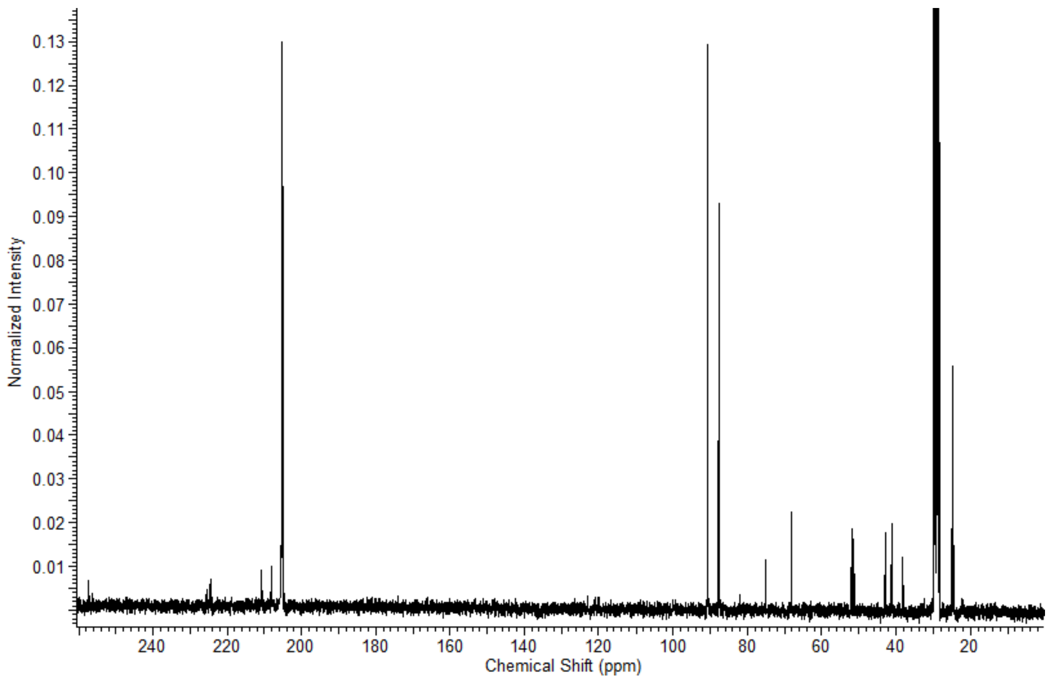

Figure S14. $^{13}C\{^{1}H\}$ NMR spectrum (101 MHz, acetone-d$_6$) of **4a**.

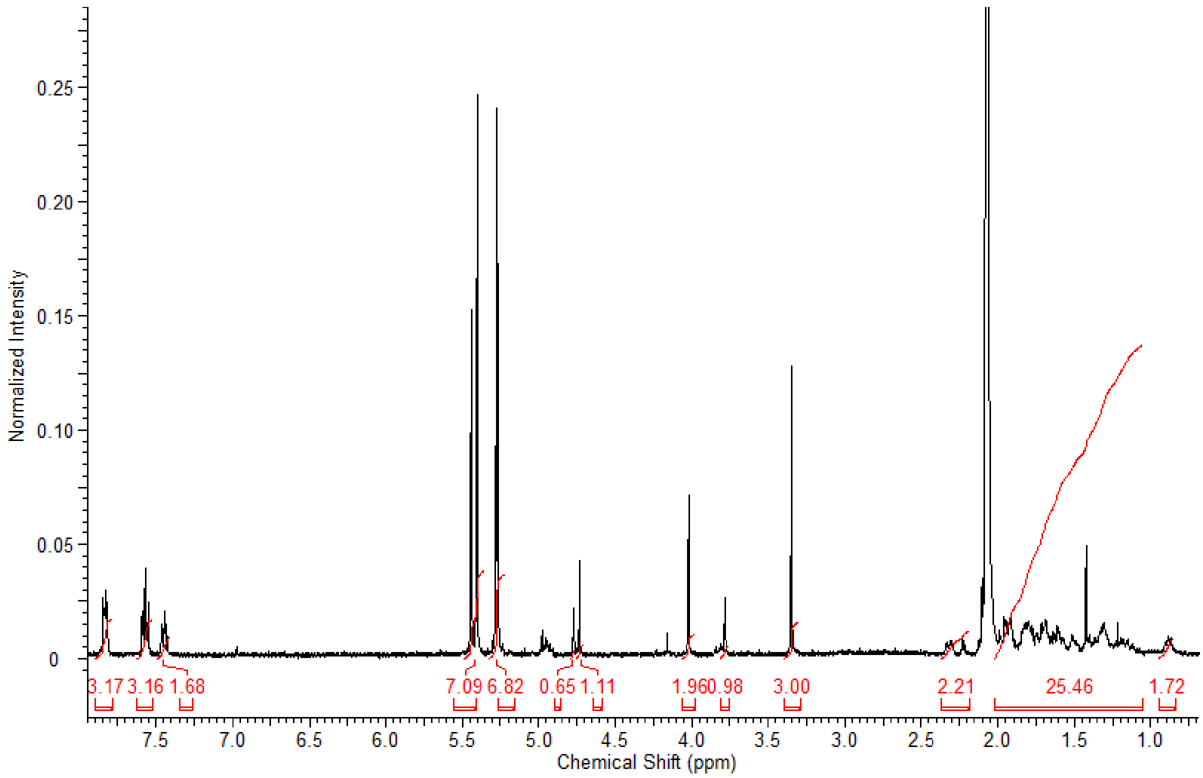

Figure S15. ^{1}H NMR spectrum (401 MHz, acetone-d$_6$) of **4b**.

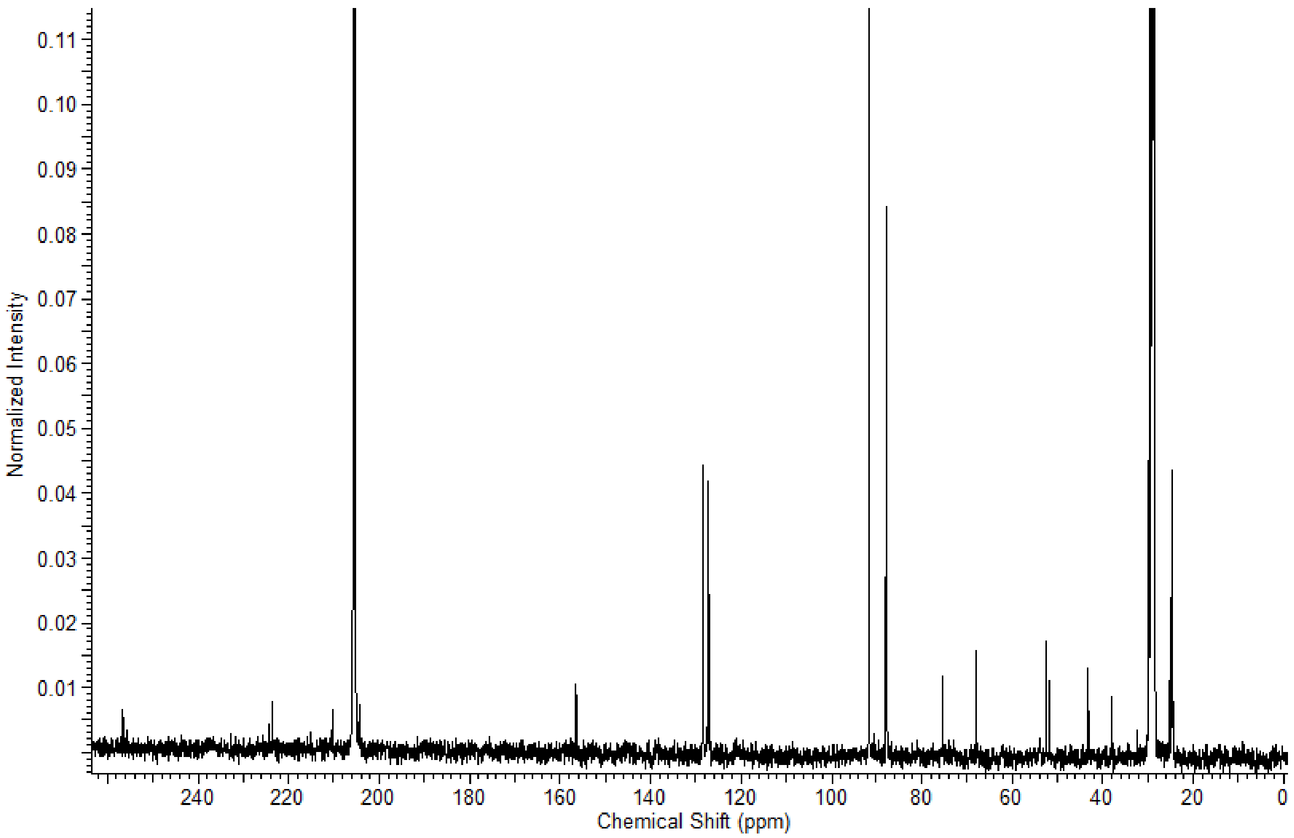

Figure S16. $^{13}C\{^{1}H\}$ NMR spectrum (101 MHz, acetone-d_6) of **4b**.

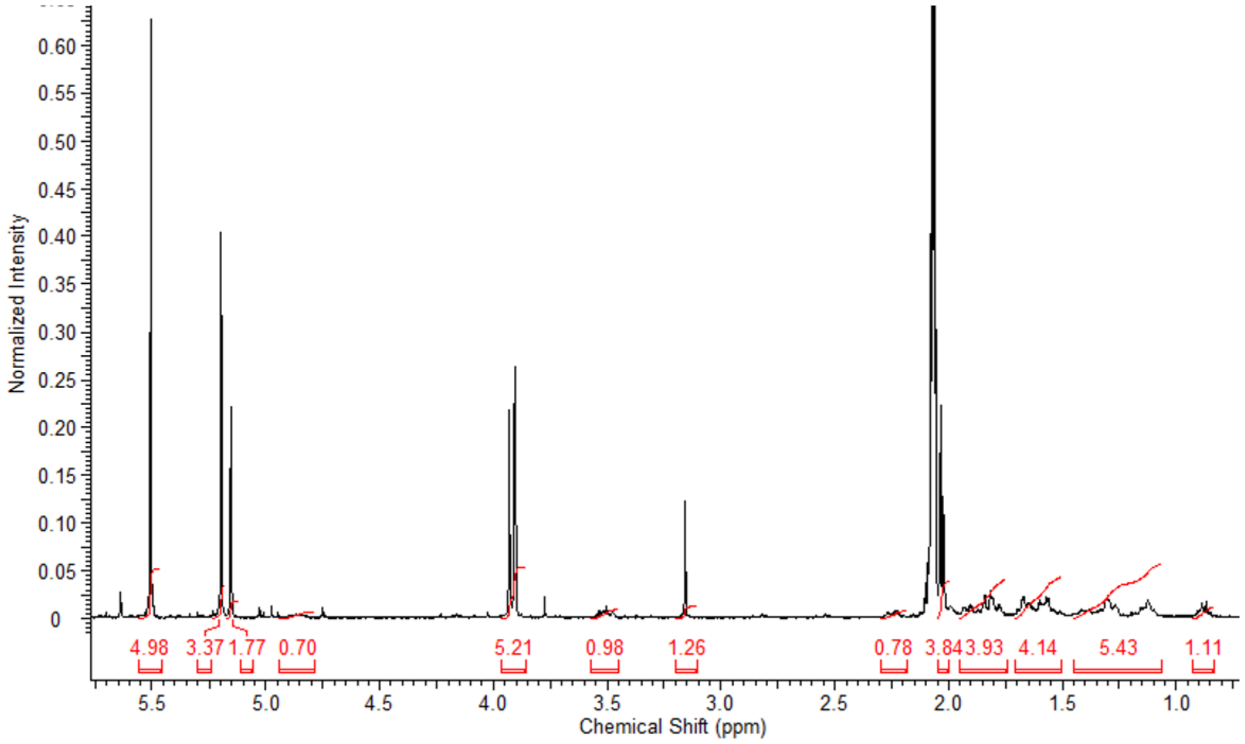

Figure S17. ^{1}H NMR spectrum (401 MHz, acetone-d_6) of **4c**.

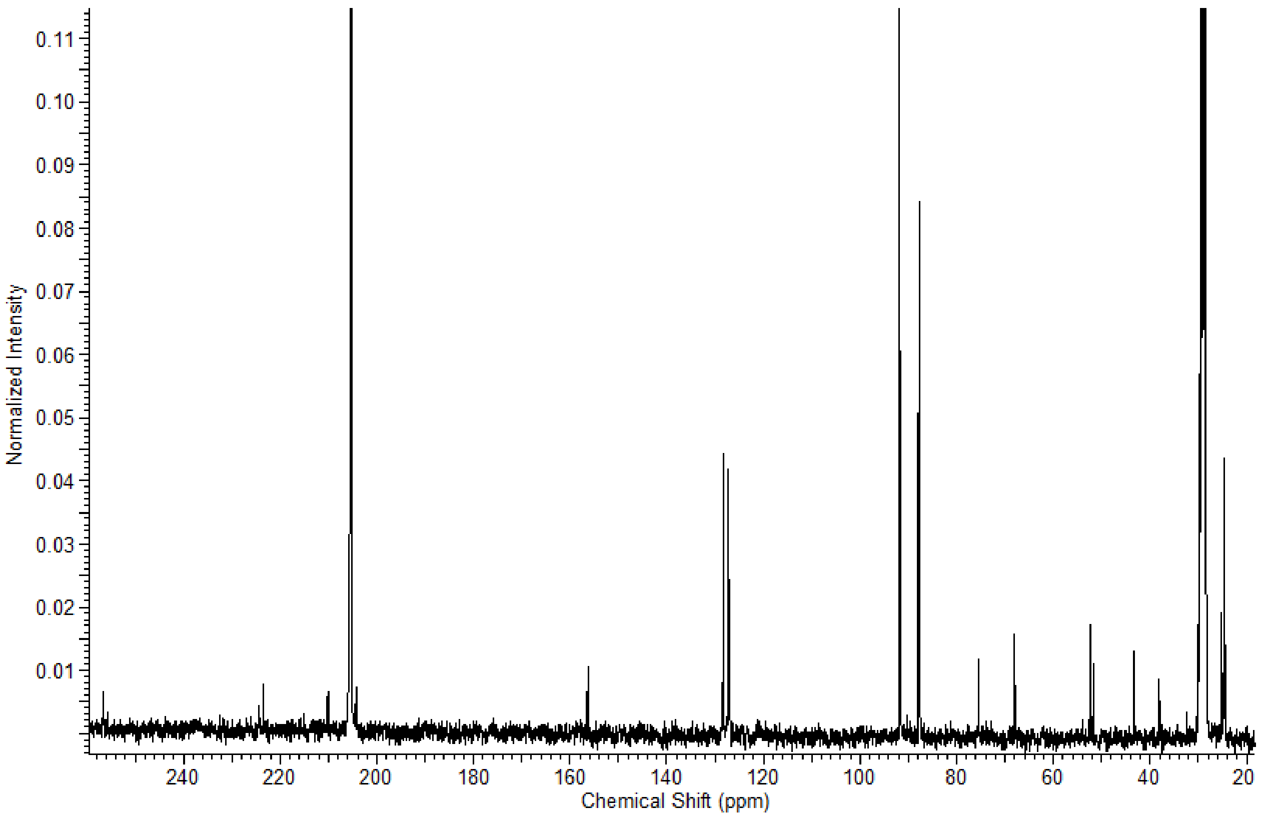

Figure S18. $^{13}C\{^{1}H\}$ NMR spectrum (101 MHz, acetone-d$_6$) of **4c**.

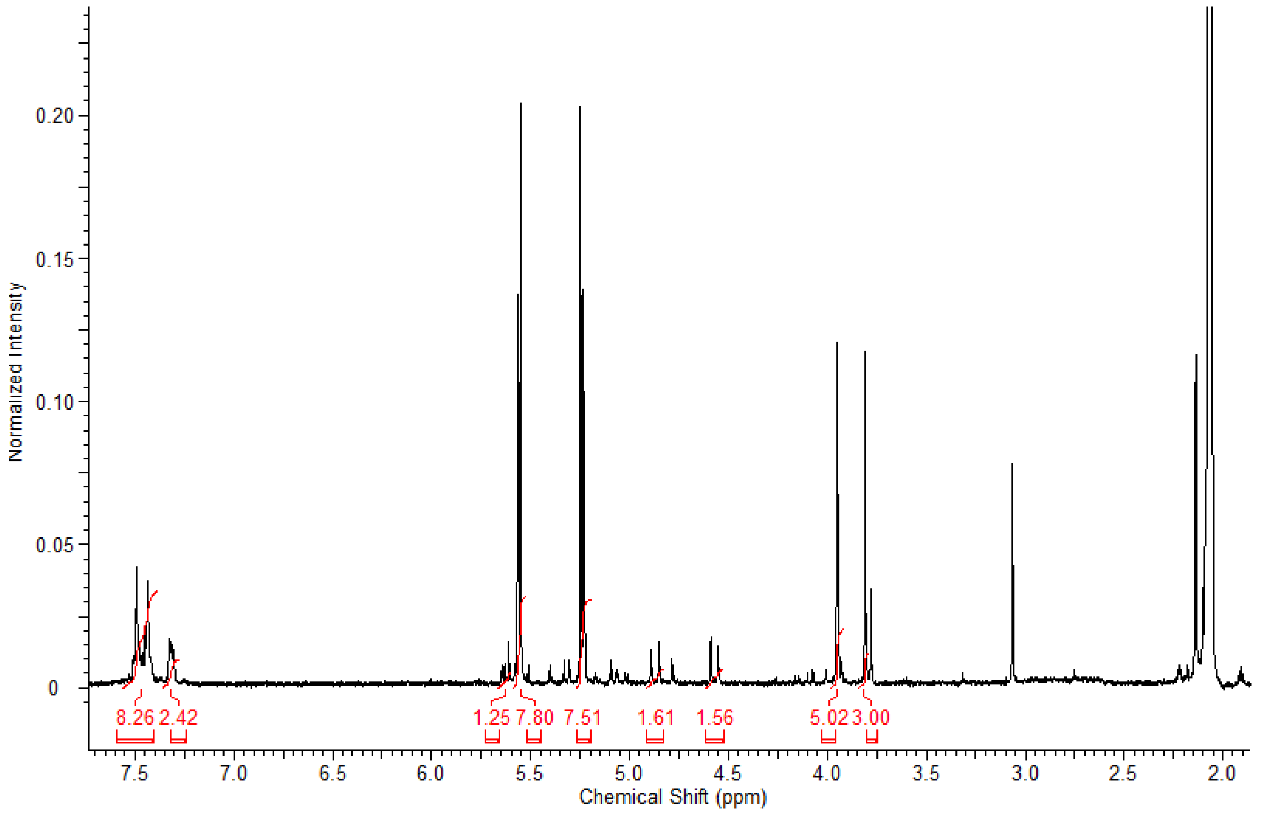

Figure S19. ^{1}H NMR spectrum (401 MHz, acetone-d$_6$) of **5a**.

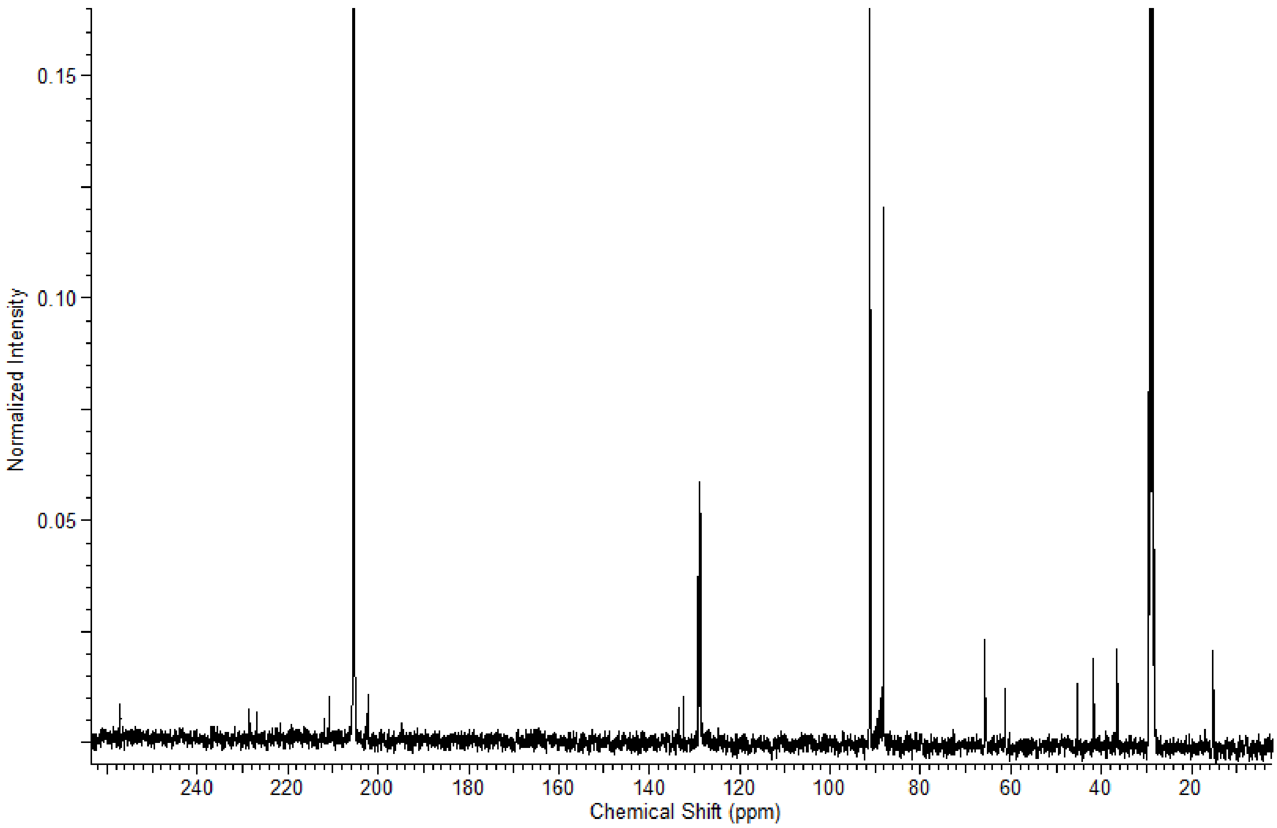

Figure S20. $^{13}C\{^1H\}$ NMR spectrum (101 MHz, acetone-d$_6$) of **5a**.

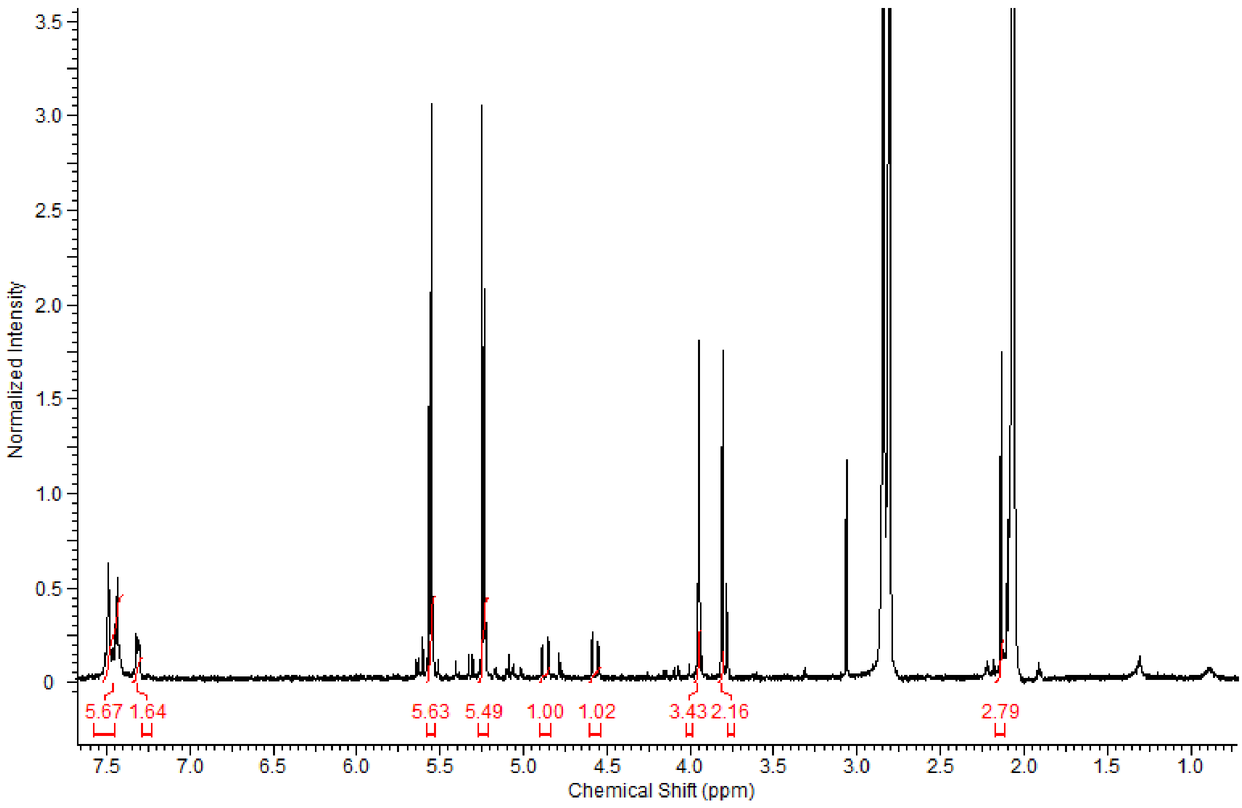

Figure S21. ^1H NMR spectrum (401 MHz, acetone-d$_6$) of **5b**.

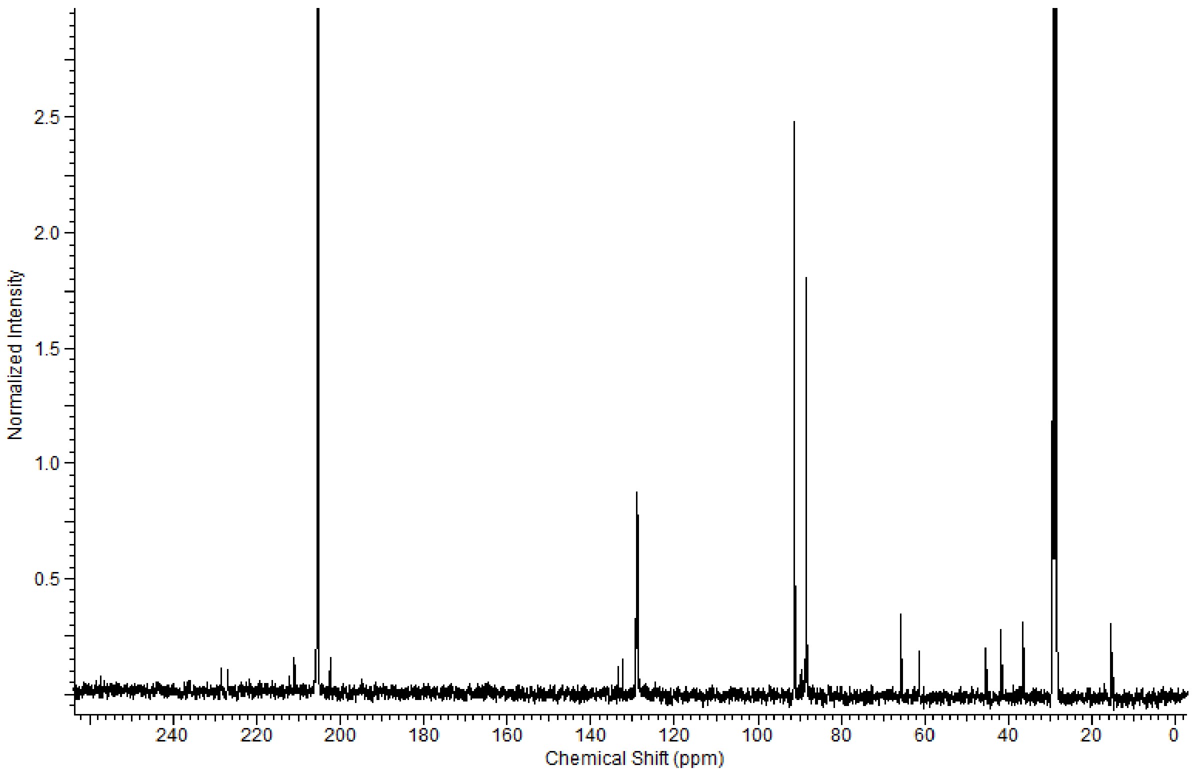

Figure S22. $^{13}C\{^{1}H\}$ NMR spectrum (101 MHz, acetone-d_6) of **5b**.

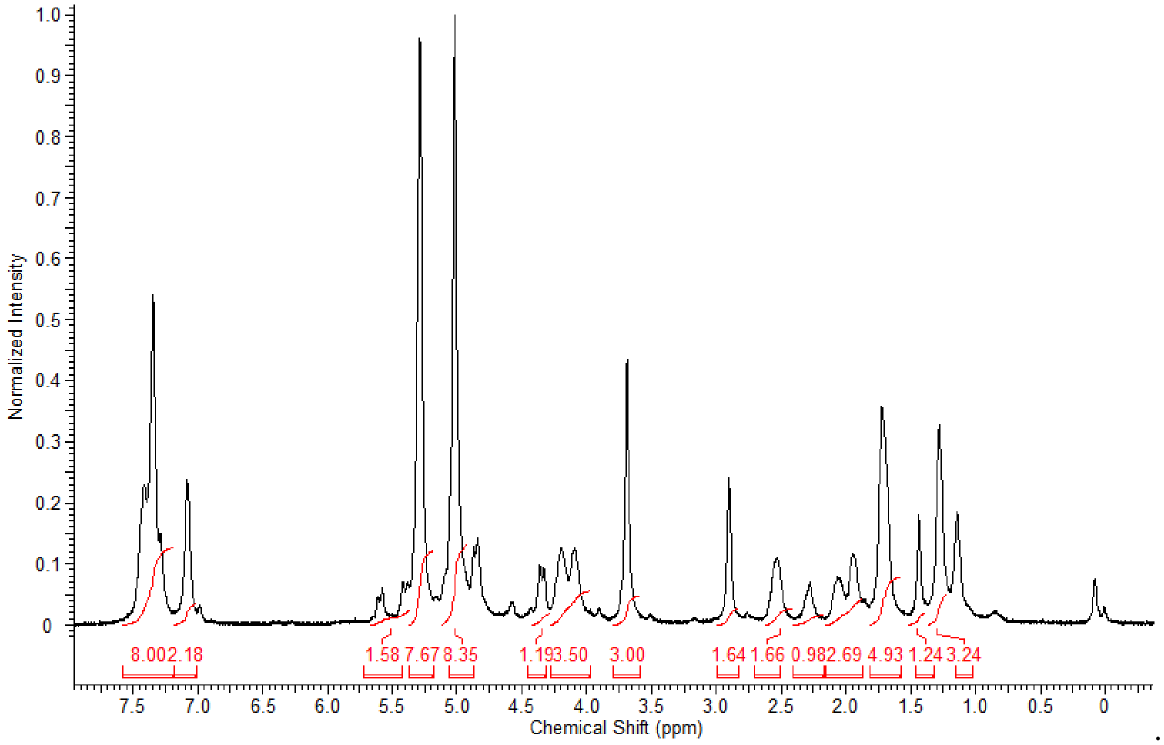

Figure S23. ^{1}H NMR spectrum (401 MHz, $CDCl_3$) of **5c**.

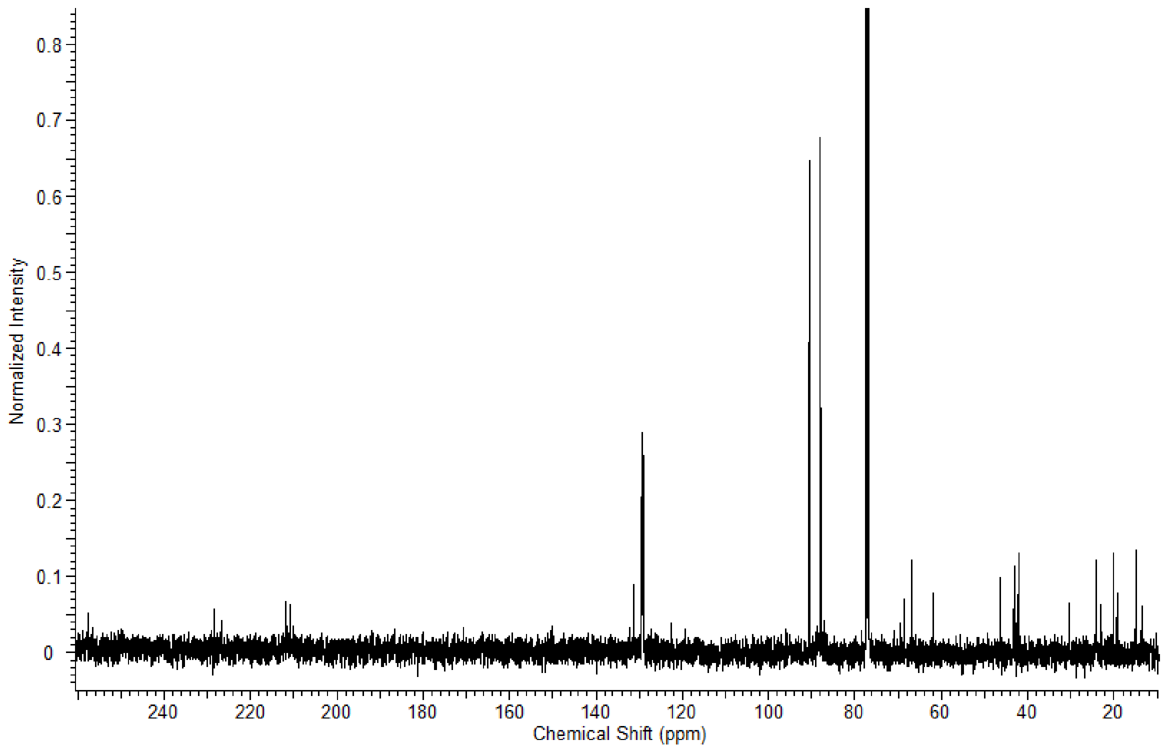

Figure S24. $^{13}C\{^1H\}$ NMR spectrum (101 MHz, $CDCl_3$) of **5c**.

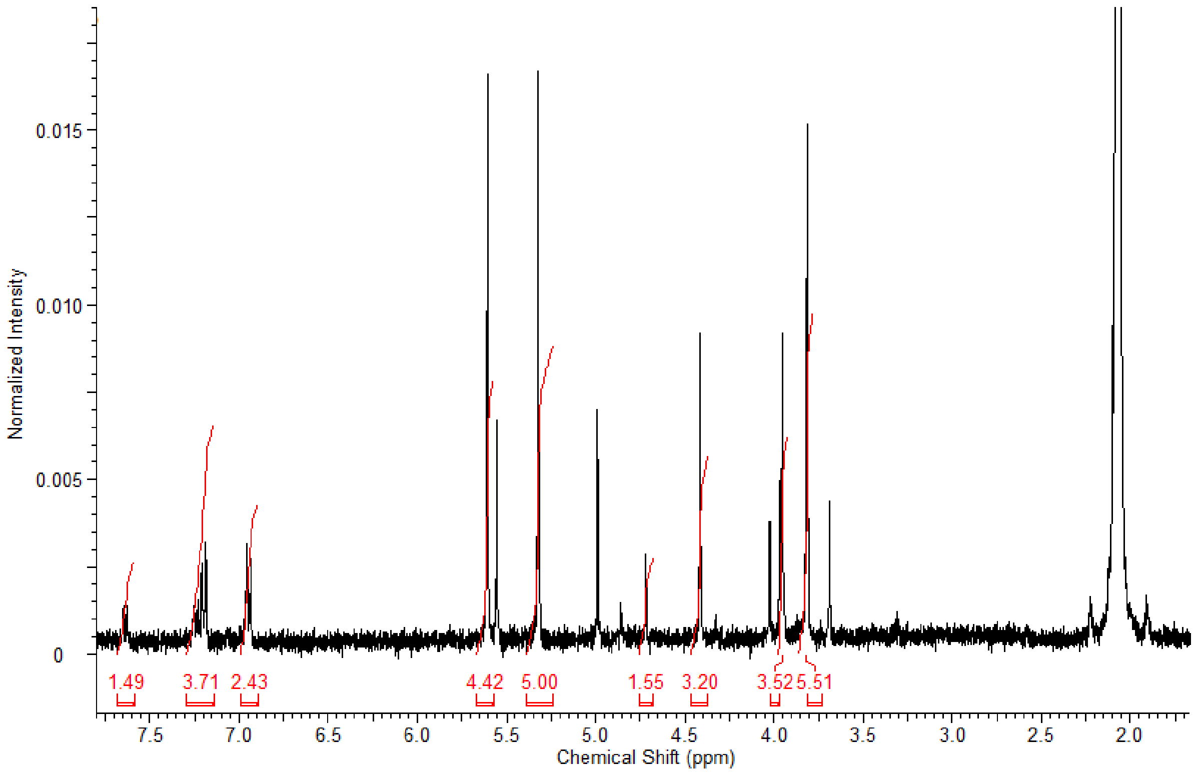

Figure S25. ^1H NMR spectrum (401 MHz, acetone-d_6) of **6a**.

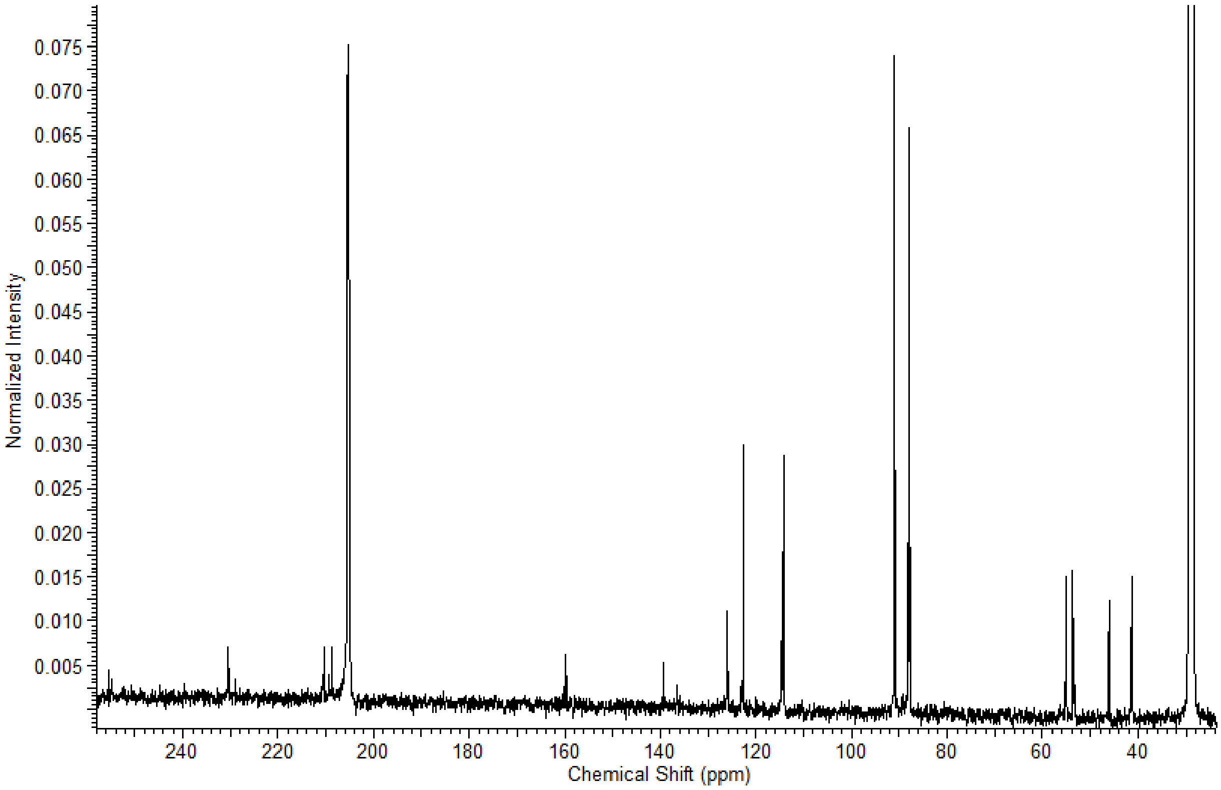

Figure S26. $^{13}C\{^{1}H\}$ NMR spectrum (101 MHz, acetone-d_6) of **6a**.

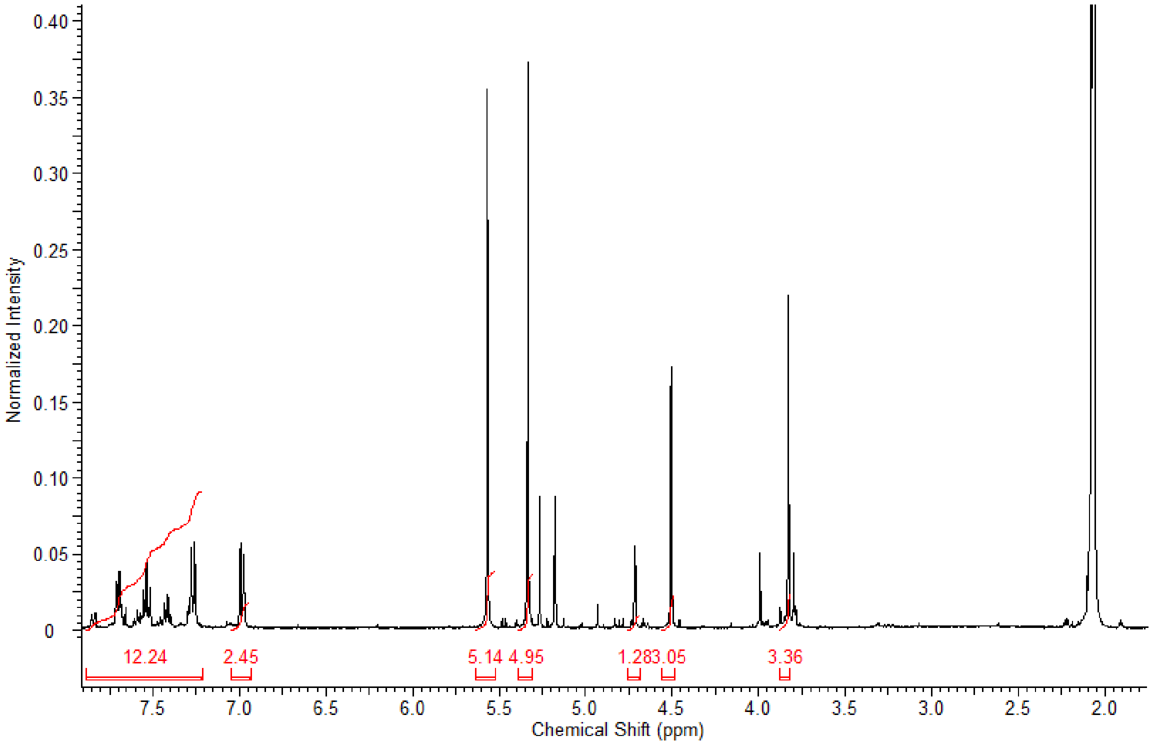

Figure S27. ^{1}H NMR spectrum (401 MHz, acetone-d_6) of **6b**.

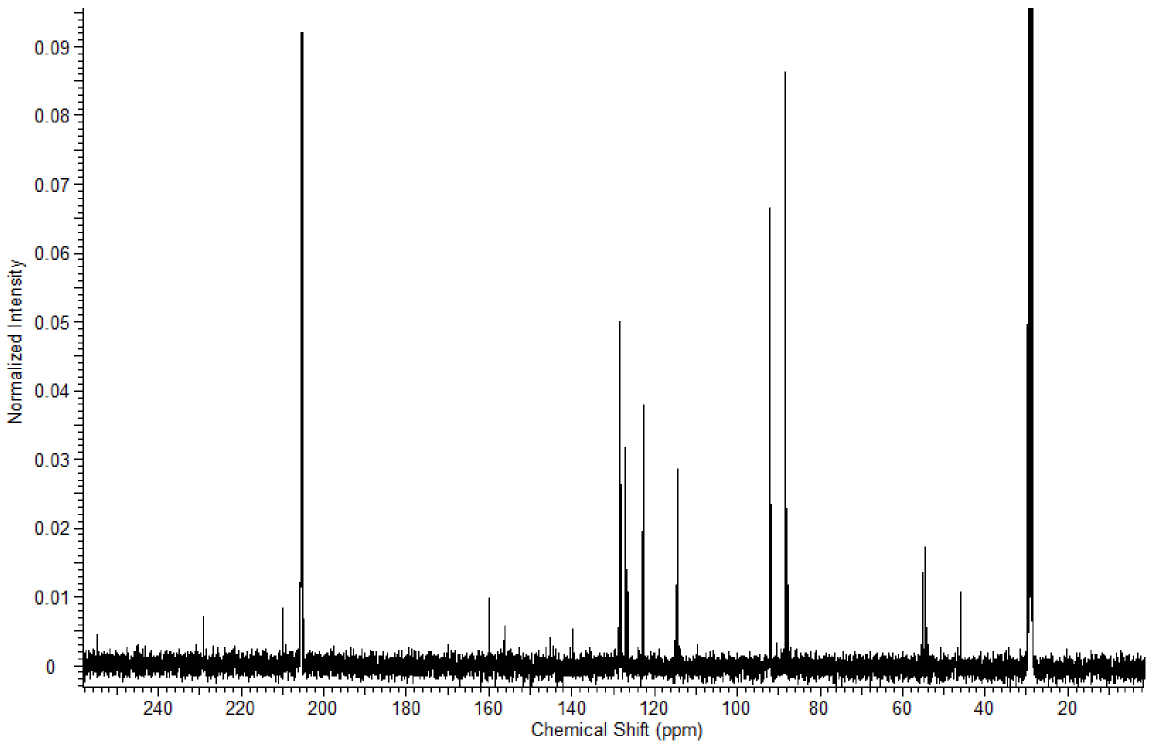

Figure S28. $^{13}C\{^{1}H\}$ NMR spectrum (101 MHz, acetone-d_6) of **6b**.

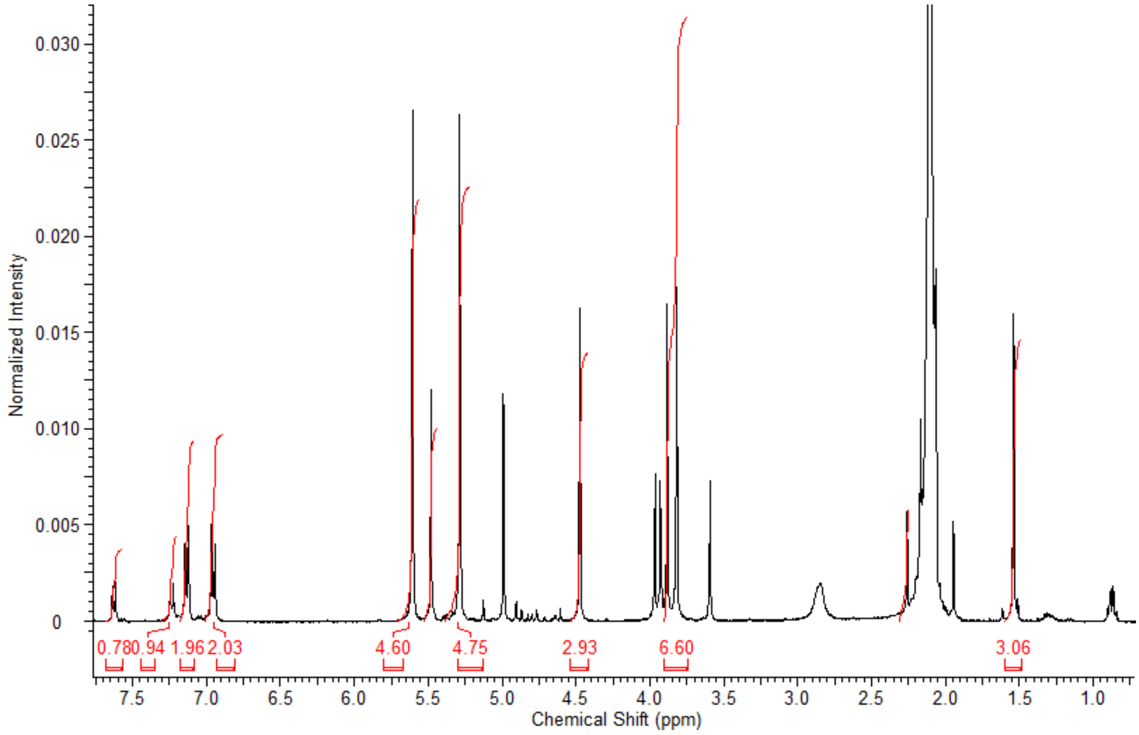

Figure S29. ^{1}H NMR spectrum (401 MHz, acetone-d_6) of **6c**.

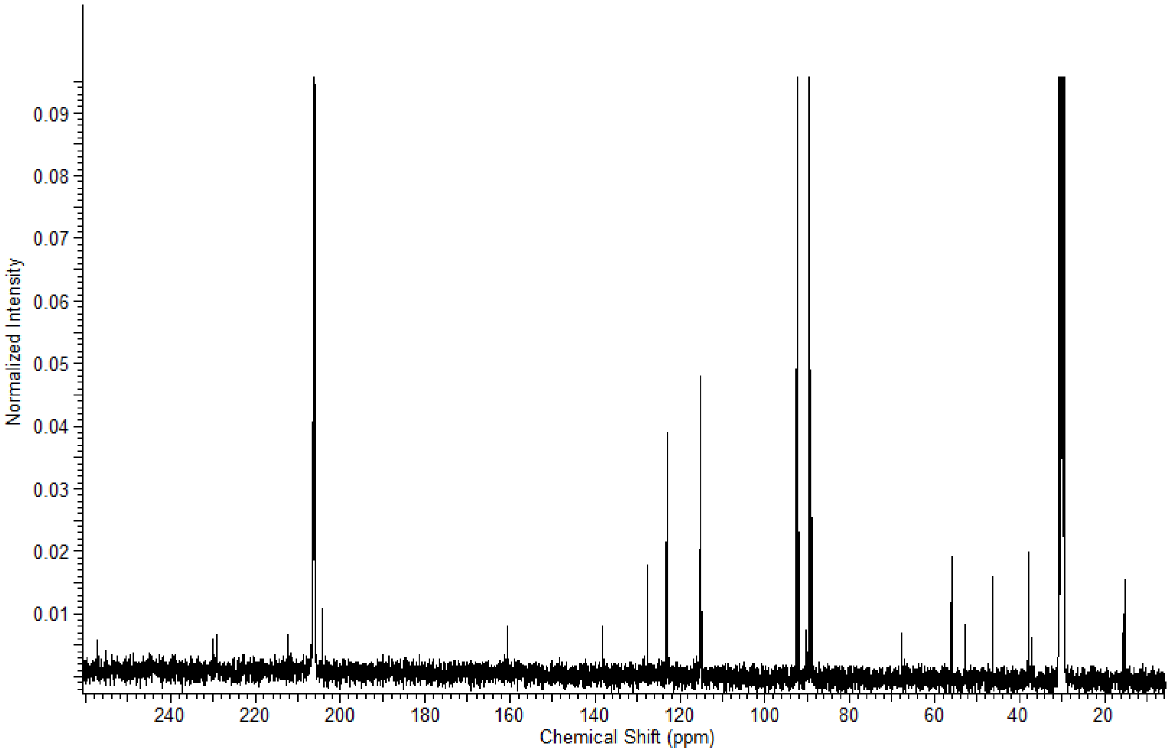

Figure S30. $^{13}C\{^{1}H\}$ NMR spectrum (101 MHz, acetone-d$_6$) of **6c**.

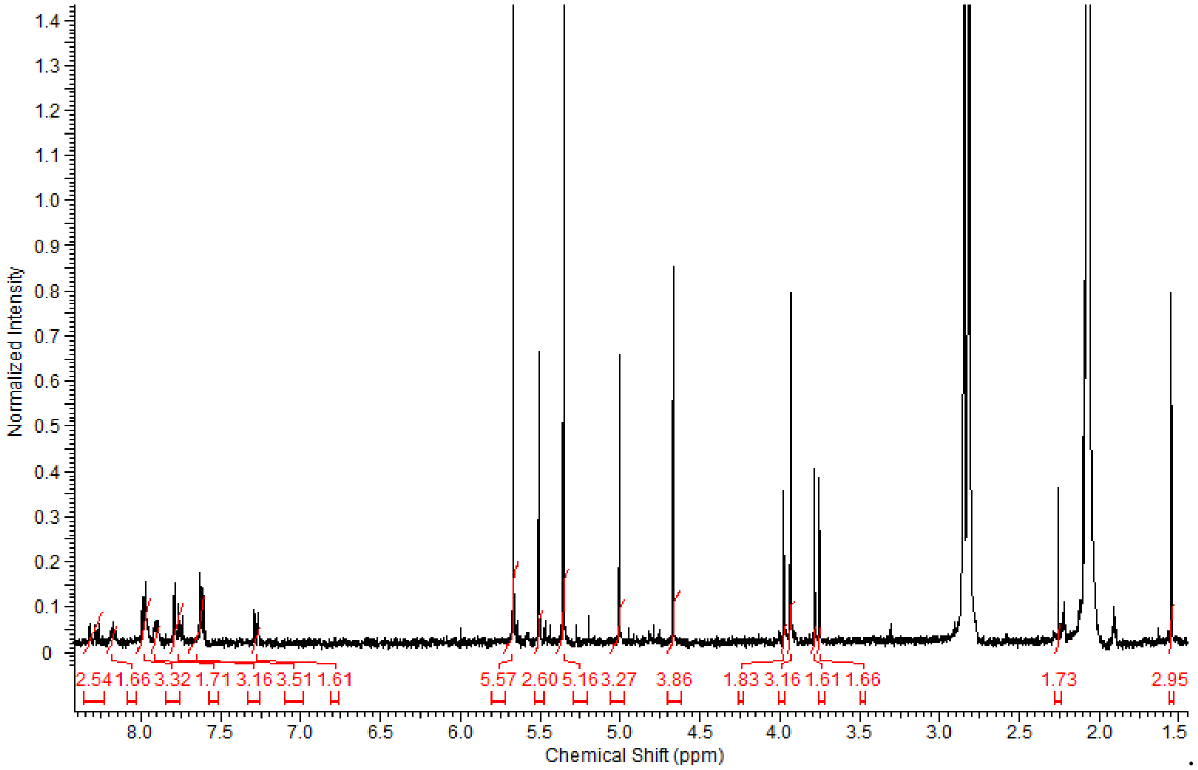

Figure S31. ^{1}H NMR spectrum (401 MHz, acetone-d$_6$) of **7**.

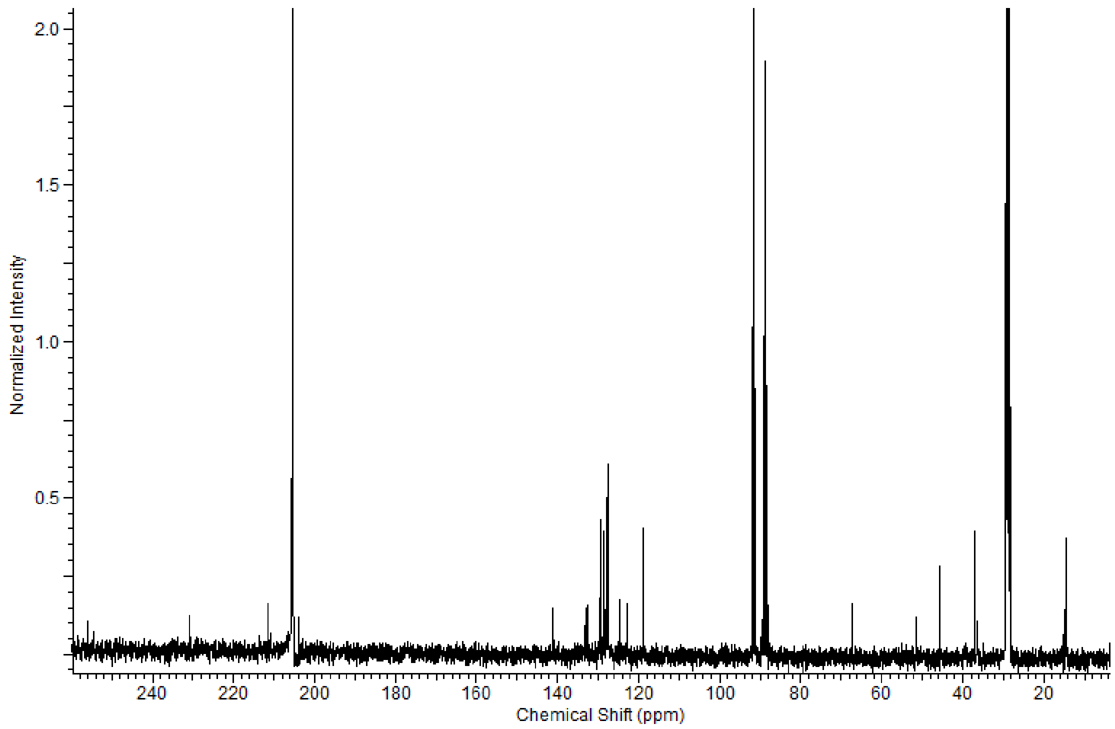

Figure S32. $^{13}C\{^{1}H\}$ NMR spectrum (101 MHz, acetone-d_6) of **7**.

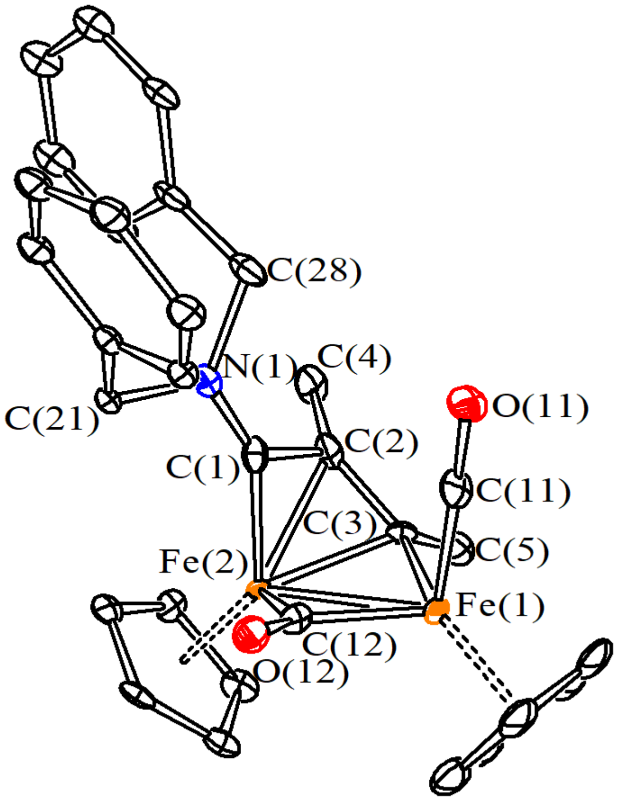

Figure S33. View of the cation of **2c**. H atoms have been omitted for clarity. Displacement ellipsoids are at the 50% probability level. Selected bond lengths (Å) and angles (°) for **3a**: Fe(1)-Fe(2) 2.540(3), Fe(1)-C(11) 1.732(17), Fe(1)-C(12) 1.898(13), Fe(2)-C(12) 1.942(12), Fe(2)-C(1) 1.816(15), Fe(2)-C(2) 2.080(14), Fe(2)-C(3) 2.052(17), Fe(1)-C(3) 1.948(15), C(1)-N(1) 1.291(14), N(1)-C(21) 1.501(13), N(1)-C(28) 1.490(14), C(11)-O(11) 1.162(19), C(12)-O(12) 1.161(16), Fe(1)-C(11)-O(11) 178.4(12), Fe(1)-C(12)-Fe(2) 82.8(5), Fe(1)-C(3)-Fe(2) 78.8(5), Fe(1)-C(3)-C(2) 121.4(11), C(3)-C(2)-C(1) 112.6(15), C(2)-C(1)-Fe(2) 78.4(9), C(1)-N(1)-C(21) 123.1(16), C(1)-N(1)-C(28) 123.2(16), C(21)-N(1)-C(28) 113.3(15).

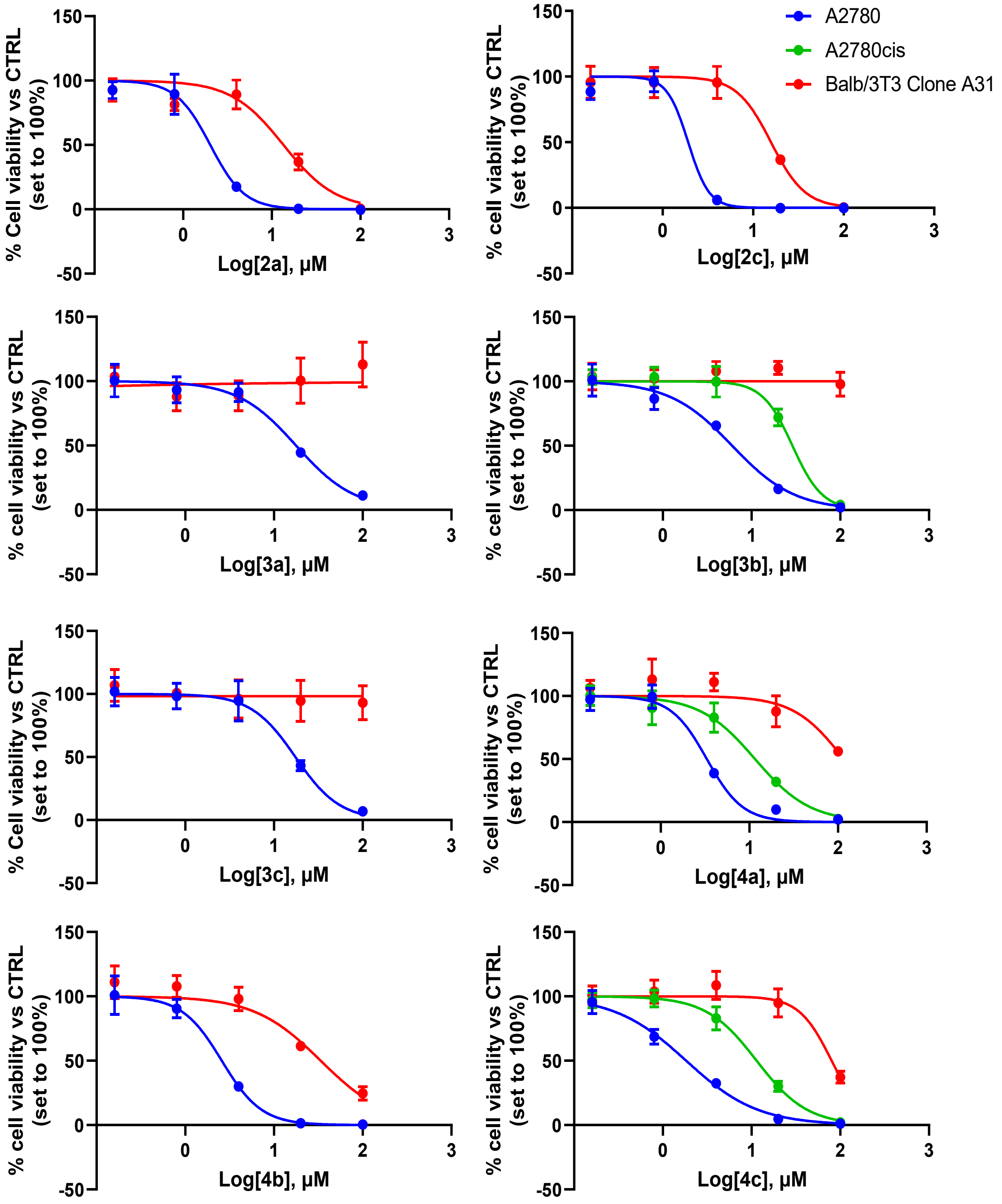

A2780
A2780cis
Balb/3T3 Clone A31
% Cell viability vs CTRL (set to 100%)
% cell viability vs CTRL (set to 100%)
Log[2a], μM
Log[2c], μM
Log[3a], μM
Log[3b], μM
Log[3c], μM
Log[4a], μM
Log[4b], μM
Log[4c], μM

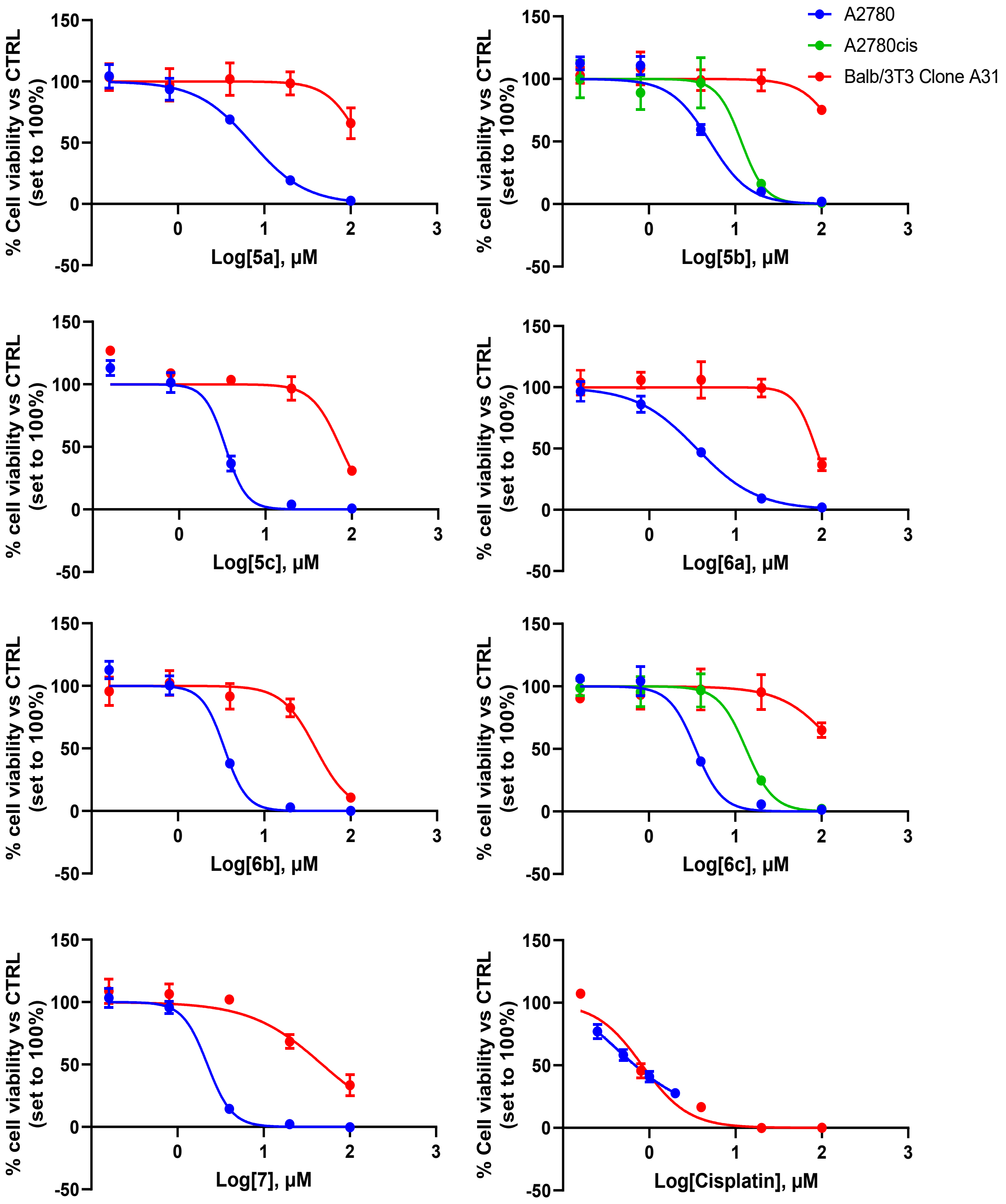

Figure S34. Dose-response curves reporting the cell viability as a function of the concentration of the complexes; cisplatin was tested as reference. The values of IC_{50} (reported in Table 3) were measured after 72 hours of incubation (see Experimental).

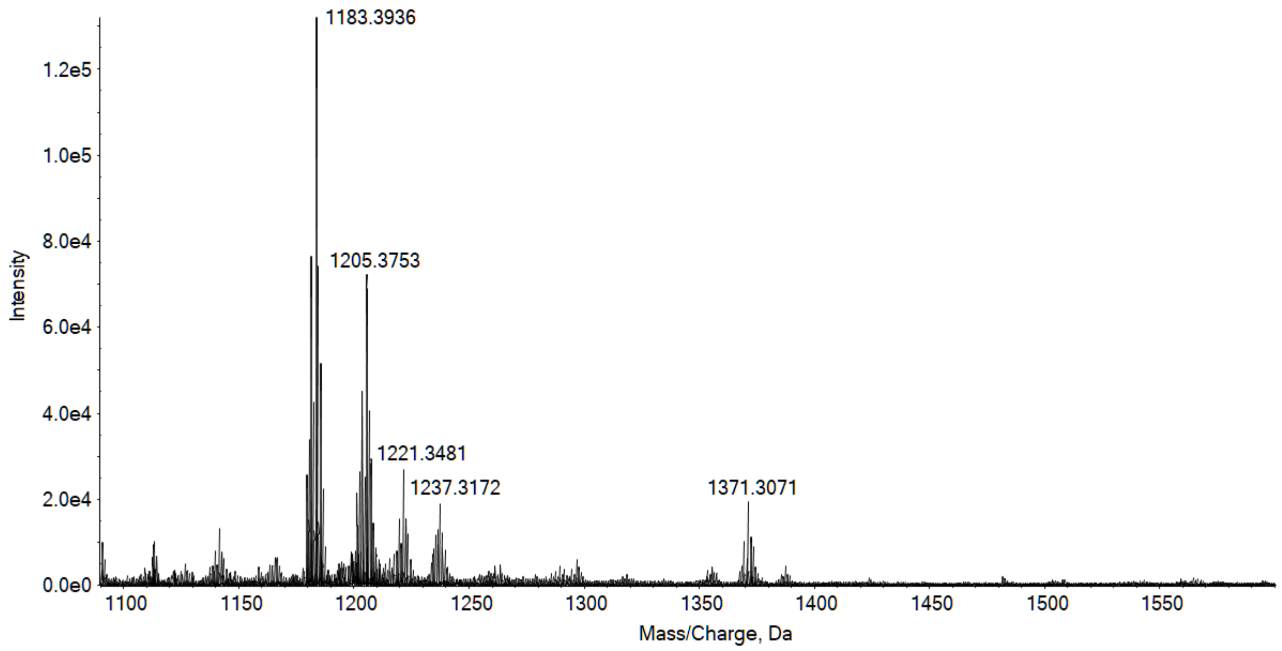

Figure S35. High-resolution ESI mass spectrum of 10^{-5} M TrxR dodecapeptide solution in water incubated with compound **3b** for 24 h at 37 °C; 1:1 peptide to complex ratio. 0.1% v/v of formic acid was added just before infusion.

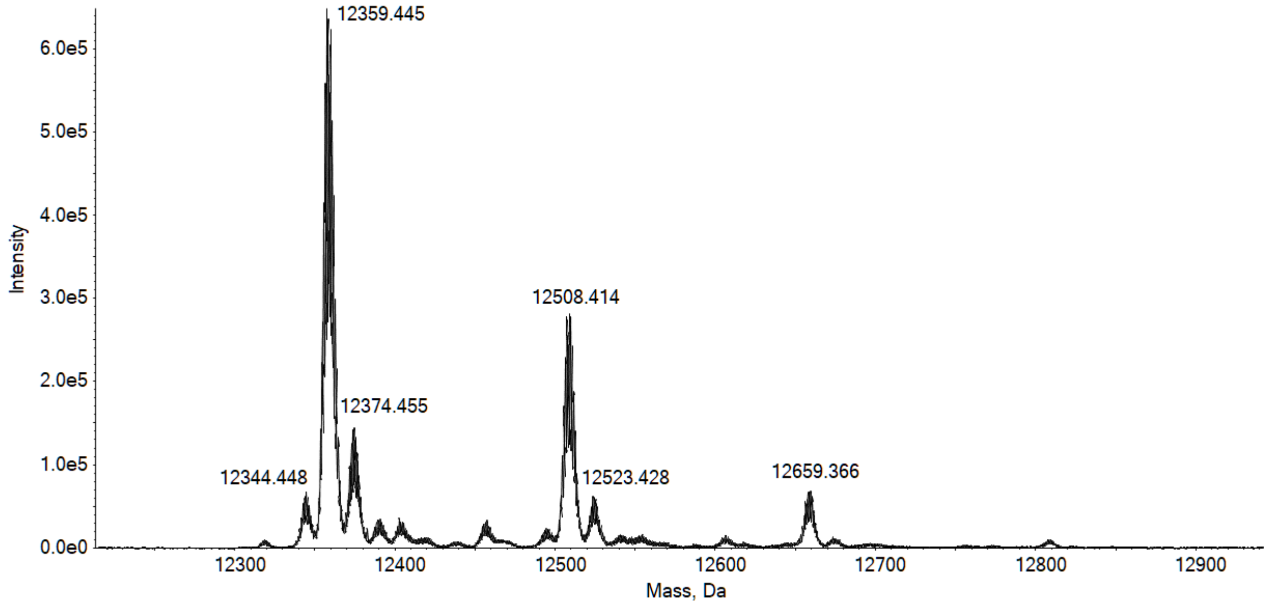

Figure S36. Deconvoluted ESI mass spectrum of Cyt c in 2 mM ammonium acetate solution, pH 6.8, incubated with compound **3b** for 24 h at 37 °C. The final protein concentration was 10^{-6} M with a complex to protein molar ratio of 2:1. 0.1% v/v of formic acid was added just before infusion.
